# Long-Term Coronary Microvascular and Cardiac Dysfunction After Severe COVID-19 Hospitalization

**DOI:** 10.1001/jamanetworkopen.2025.14411

**Published:** 2025-06-09

**Authors:** Rebecka Steffen Johansson, Daniel Loewenstein, Klara Lodin, Judith Bruchfeld, Michael Runold, Marcus Ståhlberg, Hui Xue, Peter Kellman, Kenneth Caidahl, Henrik Engblom, Jannike Nickander

**Affiliations:** 1 Department of Clinical Physiology, Karolinska University Hospital, Stockholm, Sweden; 2 Department of Molecular Medicine and Surgery, Karolinska Institutet, Stockholm, Sweden; 3 Division of Cardiology, Department of Medicine, Karolinska Institutet, Stockholm, Sweden; 4 Division of Infectious Diseases, Department of Medicine, Karolinska Institutet, Stockholm, Sweden; 5 Department of Infectious Diseases, Karolinska University Hospital, Stockholm, Sweden; 6 Department of Respiratory Medicine and Allergy, Karolinska University Hospital, Stockholm, Sweden; 7 Department of Medicine, Solna, Karolinska Institutet, Solna, Sweden; 8 Department of Cardiology, Heart and Vascular Center, Karolinska University Hospital, Solna, Sweden; 9 Health Futures, Microsoft Research, Redmond, Washington; 10 National Heart, Lung, and Blood Institute, National Institutes of Health, Bethesda, Maryland; 11 Department of Clinical Sciences Lund, Clinical Physiology, Skåne University Hospital, Lund University, Lund, Sweden

## Abstract

**Importance:**

COVID-19 can lead to long-term cardiopulmonary symptoms and is associated with coronary microvascular dysfunction (CMD). However, long-term data on CMD after severe COVID-19 are lacking.

**Objective:**

To investigate long-term left ventricular function and the presence of CMD after severe COVID-19.

**Design, Setting, and Participants:**

This case-control study was conducted among patients at a single center from the prospective Follow-Up of Patients With Severe COVID-19 (UppCov) study, with follow-up multiparametric perfusion cardiovascular magnetic resonance (CMR) at 10 months after discharge from November 2020 to February 2021. Patients previously hospitalized due to severe COVID-19 (ventilatory support, oxygen flow ≥5 L/min, or both) with or without cardiac involvement (troponin *t* > 14 ng/L, pulmonary artery pressure >34 mm Hg, or both) were compared with historical sex- and age-matched volunteers without symptomatic ischemic heart disease. Standard contraindications to adenosine CMR were applied. Data were analyzed between March 2023 and March 2025.

**Exposure:**

Hospitalization due to severe COVID-19.

**Main Measures and Outcomes:**

Comprehensive CMR included native T1, native T2, extracellular volume, adenosine stress and rest perfusion mapping, gadolinium enhancement, and cine imaging. Comorbidities, medications, symptoms at follow-up, and details regarding hospitalization were obtained from patient records.

**Results:**

The study included 37 patients with COVID-19 (mean age, 56 years [95% CI, 53 to 61 years]; 28 male [75.7%]) and 22 healthy volunteers (mean age, 51 years [95% CI, 45 to 57 years]; 12 male [54.4%]). Patients with COVID-19 compared with healthy patients demonstrated reduced mean stress perfusion (2.80 mL/min/g [95% CI, 2.53 to 3.07 mL/min/g] vs 3.43 mL/min/g [95% CI, 3.13 to 3.74 mL/min/g]; *P* = .003), impaired mean global longitudinal strain (−17% [95% CI, −18% to −16%] vs −19% [−20% to −18%]; *P* = .003), and impaired mean global circumferential strain (−16% [95% CI, −17% to −15%] vs −19% [−20% to −18%]; *P* = .001). There were no differences in stress perfusion or myocardial perfusion reserve in the COVID-19 group between patients with vs without cardiovascular risk factors or cardiac symptoms.

**Conclusions and Relevance:**

In this study, patients with COVID-19 exhibited long-term reduced stress perfusion indicating CMD, along with declined left ventricular function by global longitudinal strain and global circumferential strain. Lack of variation in stress perfusion between patients with and without cardiovascular risk factors may suggest CMD due to severe COVID-19, warranting further investigation to elucidate mechanisms and guide potential therapies.

## Key Points

**Question:**

Is there long-term left ventricular dysfunction and coronary microvascular dysfunction after severe COVID-19?

**Findings:**

This case-control study used multiparametric adenosine stress perfusion cardiovascular magnetic resonance imaging to evaluate 37 patients with COVID-19 in long-term follow-up compared with 22 healthy volunteers of similar age and sex. Patients with COVID-19 had statistically significantly impaired global longitudinal and circumferential strain and reduced stress perfusion compared with healthy volunteers.

**Meaning:**

These findings suggest the presence of left ventricular systolic dysfunction and coronary microvascular dysfunction among patients hospitalized with COVID-19.

## Teaser

This case-control study investigates long-term left ventricular dysfunction and coronary microvascular dysfunction outcomes after hospitalization for severe COVID-19.

## Introduction

COVID-19 primarily presents with respiratory symptoms of varying severity. However, cardiac complications are common and correlate with disease severity and risk of mortality.^1^ Nonhospitalized and hospitalized patients with COVID-19 have long-term risk of arrhythmias, ischemic and nonischemic heart disease, heart failure, perimyocarditis, and thromboembolic events, with the highest risk in patients treated in the intensive care unit during the first waves of the pandemic.^2^ Moreover, in long COVID^3^ or postacute sequelae of SARS-CoV-2 infection (PASC),^4^ patients commonly experience cardiopulmonary symptoms, including dyspnea, palpitations, chest pain, and fatigue, which impair quality of life and functional capacity.^5,6,7^ The underlying pathophysiological mechanisms are not fully understood but may stem from myocardial injury sustained during acute COVID-19 due to hypoxia, systemic hyperinflammation, hypercoagulability, and direct viral invasion of endothelial cells and cardiomyocytes.^8,9^ Notably, microvascular dysfunction across various vascular beds, including the coronary circulation, has been documented after COVID-19.^10,11,12,13^ Coronary microvascular dysfunction (CMD) may manifest as chest pain with or without obstructive coronary artery disease, and traditional cardiovascular risk factors are associated with CMD and risk of severe COVID-19.^14^ CMD can be detected using quantitative cardiovascular magnetic resonance (CMR) adenosine stress perfusion mapping.^15^ Moreover, comprehensive multiparametric CMR may characterize cardiac anatomy, function, and tissue properties.^16^ Despite the critical need for understanding long-term cardiovascular implications in PASC, long-term data on patients with critical illness are limited. Thus, the aim of this study was to evaluate the presence of CMD in patients hospitalized due to severe COVID-19 in long-term follow-up using multiparametric stress perfusion CMR.

## Methods

### Study Population

Patients for this case-control study were identified from the prospective study Follow-Up of Patients With Severe COVID-19 (UppCov), aiming to comprehensively assess long-term outcomes after hospitalization due to severe COVID-19, at Karolinska University Hospital in Stockholm, Sweden.^17^ Severe COVID-19 was defined as respiratory failure requiring ventilatory support, oxygen therapy (oxygen flow ≥5 L/min), or both. In total, 40 patients with COVID-19 with and without cardiac involvement during hospitalization were included from November 2020 to February 2021 for CMR at approximately 10 months’ follow-up. Cardiac involvement was defined as high-sensitivity troponin T (hsTnT) level greater than 14 ng/L, pulmonary artery pressure (PAP) greater than 34 mm Hg, or both. Exclusion criteria were claustrophobia, presence of a pacemaker or CMR-incompatible metal implants, severe asthma or chronic obstructive pulmonary disease (COPD), high-degree atrioventricular block, and kidney failure (estimated glomerular filtration rate <30 mL/min/1.73 m^2^). Patients with known angina pectoris, previous myocardial infarction, coronary artery bypass grafting or percutaneous coronary intervention, stroke, heart failure, aortic stenosis, or arrhythmias, including atrial fibrillation, were excluded. Historical volunteers^18,19^ without symptomatic ischemic heart disease were selected to match the patient group by sex and with an age range of 10 years; however, volunteers of the same sex and age were not available for all patients. Patients with COVID-19 were invited to clinical follow-up at a median (IQR) of 244 (214-288) days after discharge. Residual respiratory symptoms were evaluated using the COPD Assessment Test (CAT)^20^and Modified Medical Research Council Dyspnea Scale (mMRC).^21^ Quality of life was assessed using the EuroQol Visual Analogue Scale (EQ-VAS).^22^ Clinical data, including comorbidities, medications, and details regarding hospitalization and clinical follow-up, were obtained from medical records and by interviews. All procedures were granted ethical approval by the Swedish Ethical Review Authority, with the original institutional review board approval for UppCov amended to include the magnetic resonance imaging in this study; the original approval for the historical control group has existing amendments. Participants provided written informed consent. All results were reported according to the Strengthening the Reporting of Observational Studies in Epidemiology (STROBE) reporting guideline.

### Image Acquisition

Patients and volunteers underwent the same protocol, which is shown in its entirety in the eAppendix in Supplement 1. CMR was performed supine with a 1.5 T Aera scanner (Siemens Healthineers), which included a phased-array 18-channel body matrix coil and a spine matrix coil. Hematocrit and creatinine were determined prior to CMR. Full-coverage retrospective electrocardiographic–gated balanced steady-state free precession cine imaging was acquired in short-axis and 3 long-axis slices.

Quantitative perfusion maps were acquired in 3 short-axis slices using first-pass perfusion imaging during adenosine infusion (140 μg/kg/min or increased according to clinical routine to 210 μg/kg/min [Adenosin, Life Medical AB]) and in rest during injection of intravenous contrast (0.05 mmol/kg, gadobutrol; Gadovist, Bayer AB). Participants abstained from caffeine for 24 hours and nicotine for 12 hours prior to CMR. Adenosine response was assessed by symptoms and heart rate response. Contrast and adenosine were administered in separate cannulas. Perfusion maps were computed using the distributed tissue-exchange model^23^ and generated using Gadgetron inline perfusion mapping software.^24,25^

Native T2 maps were acquired in 3 or 5 short-axis slices using a T2-prepared sequence (Siemens MyoMaps product sequence). Native T1 maps were acquired in 3 or 5 short-axis slices using an electrocardiographic–gated modified Look-Locker inversion recovery^26^ 5s(3s)3s research sequence. Postcontrast T1 maps were acquired after a contrast bolus (0.2 mmol/kg; gadobutrol) with the same image positions as the native T1 maps. Extracellular volume (ECV) maps were generated from native and postcontrast T1 maps and calibrated by hematocrit.^27^ Furthermore, postcontrast late gadolinium enhancement (LGE) images were acquired in short-axis and 3 long-axis slices using a free-breathing phase-sensitive inversion recovery sequence with balanced steady-state free precession single-shot readout.

### Image Analysis

Images were anonymized and analyzed offline using Segment software version 2.7 (Medviso AB).^28,29^ Left ventricular (LV) volumes and mass were acquired using automatic segmentation of the cine short-axis stack in end diastole and end systole, with manual corrections. LV volumes and mass were indexed to body surface area calculated with the Mosteller formula.^30^ Global longitudinal strain (GLS) and global circumferential strain (GCS) were acquired using the feature-tracking module in Segment after delineation in end diastole of the LV endocardial and epicardial borders in the cine long-axis slices and short-axis stack. Native T1, native T2, ECV, and perfusion maps were analyzed by manually delineating epicardial and endocardial contours in respective short-axis stacks. To avoid contamination from the blood pool and adjacent tissues, a 10% erosion margin was set for endocardial and epicardial borders. Segmental values were acquired in a 16-segment LV model.^31^ Interobserver analysis was performed in all patients by 2 separate observers (R.S.J. and K.L.), and intraobserver analysis was performed in 10 patients by 1 observer (R.S.J.) by repeated analysis of the short-axis cine stack, native T1, native T2, ECV, and perfusion maps.

### Statistical Analysis

Normality was assessed with the Shapiro-Wilk test. Continuous data were presented as mean with 95% CI or median with IQR and categorical data as number with percentage. Global native T1, native T2, ECV, and perfusion values were acquired by finding the mean of segmental values. Myocardial perfusion reserve (MPR) was calculated as stress perfusion divided by rest perfusion. Rest perfusion was adjusted for rate pressure product, which was resting heart rate multiplied by resting systolic blood pressure. Patients and volunteers were compared with the independent *t* test, Mann-Whitney *U* test, or Fisher exact test as appropriate. Linear regression was assumed for stress perfusion, GLS, and GCS, and multivariable linear regression was used to investigate the association of COVID-19 disease with the respective measure when adjusting for age and sex. The significance level after Bonferroni correction for multiple testing was set to *P* < .017. Intraobserver and interobserver agreement for LV ejection fraction (LVEF), GLS, GCS, global native T1, native T2, ECV, rest and stress perfusion, and MPR were calculated as the intraclass correlation coefficient using 2-way random effects with absolute agreement. The intraclass correlation coefficient ranged from 0.72 to 1.00. Excel version 16.6 (Microsoft) and SPSS statistical software version 28 (IBM) were used for statistical analysis. Data were analyzed from March 2023 to March 2025

## Results

### Hospitalization Due to COVID-19

There were 37 patients with COVID-19 (mean age, 56 years [95% CI, 53-61 years]; 28 male [75.7%]) and 22 healthy volunteers (mean age, 51 years [95% CI, 45-57 years]; 12 male [54.4%]) in the study. Patients with COVID-19 were hospitalized a median (IQR) of 37 (20-54) days from March to September of 2020. Symptoms at presentation, presence and grade of acute respiratory distress syndrome, and details regarding hospitalization and major complications are presented in Table 1. During hospitalization among patients with COVID-19, 33 patients (89.2%) had hsTnT levels greater than 14 ng/L and 11 patients (29.7%) had PAP greater than 34 mm Hg; all but 1 patient with elevated PAP also had elevated hsTnT levels. During hospitalization, the peak median (IQR) hsTnT level was 51 (23-174) ng/L and the peak median (IQR) PAP was 50 (40-53) mmHg. Pulmonary embolism was present in 9 patients (81.8%) with elevated PAP.

**Table 1.** Symptom and Treatment Characteristics

Characteristic	Patients with COVID-19 (n = 37), No. (%)
Fever	35 (94.6)
Cough	34 (91.9)
Dyspnea	32 (86.5)
Myalgia	14 (37.8)
GI symptoms	13 (35.1)
Anosmia	2 (5.4)
ARDS	30 (81.1)
Severe	22 (73.3)
Moderate	6 (20.0)
Mild	2 (6.7)
Mechanical ventilation	28 (75.7)
ECMO	5 (13.5)
CRRT	12 (32.4)
PE	13 (35.1)
Arrhythmias	13 (35.1)
Bacterial pneumonia	11 (29.7)
Sepsis	13 (35.1)

Abbreviations: ARDS, acute respiratory distress syndrome; CRRT, continuous renal replacement therapy; ECMO, extracorporeal membrane oxygenation; GI, gastrointestinal; PE, pulmonary embolism.

### Clinical Characteristics and Follow-Up

Comorbidities and medications are presented in Table 2. There were no differences in cardiovascular risk factors or medications between patients with COVID-19 and healthy volunteers. Clinical characteristics at the time of CMR, including sex, age, and body composition, are presented in Table 2. Age and sex did not differ between study groups; however, patients with COVID-19 had a greater mean weight (88 kg [95% CI, 82-95 kg] vs 73 kg [95% CI, 68-79 kg]; *P* = .001) and body mass index (calculated as weight in kilograms divided by height in meters squared; 29 [95% CI, 27-30] vs 24 [95% CI, 22-25]; *P* < .001) compared with volunteers. Clinical follow-up was performed at a median (IQR) 244 (214-288) days after discharge. At follow-up among patients with COVID-19, persistent cough was reported in 10 patients (27.0%), dyspnea in 16 patients (43.2%), chest pressure in 7 patients (18.9%), and fatigue in 13 patients (35.1%). The mean CAT score was 10 (95% CI, 7-13), and the median mMRC score was 1 (95% CI, 1-1.75) (data were missing for 6 patients). The mean EQ-VAS score was 72 (95% CI, 65-79) (data were missing for 7 patients). At clinical follow-up, 11 of 25 patients (44.0%) who worked prior to hospitalization were still on sick leave; 12 of all 37 patients (32.4%) were retirees.

**Table 2.** Study Population Clinical Characteristics

Clinical characteristic	Participants, No. (%)		*P* value^a^
	Patients with COVID-19 (n = 37)	Volunteers (n = 22)	
Sex			
Male	28 (75.7)	12 (54.5)	.15
Female	9 (24.3)	10 (45.5)	
Age, mean (95% CI), y	56 (53-61)	51 (45-57)	.19^b^
Body composition, mean (95% CI)			
Height, cm	175 (172-178)	175 (170-179)	.96
Weight, kg	88 (82-95)	73 (68-79)	.001
BSA, m^2^	2.1 (2.0-2.2)	1.9 (1.8-2.0)	.006
BMI	29 (27-30)	24 (22-25)	<.001^b^
Laboratory values, mean (95% CI)			
Creatinine, mg/dL	0.97 (0.88-1.05)	0.87 (0.80-0.93)	.19^b^
Hematocrit, %	43 (41-44)	43 (41-44)	.68^b^
Heart rate rest, bpm	67 (64-71)	72 (68-76)	.11
SBP rest, mmHg	130 (124-135)	123 (116-129)	.11
RPP rest	8747 (8125-9370)	8815 (8116-9514)	.89
Heart rate stress, bpm	90 (86-95)	91 (86-96)	.81
Comorbidities			
Hypertension	6 (16.2)	3 (13.6)	>.99
Diabetes	3 (8.1)	1 (4.5)	>.99
Hyperlipidemia	4 (10.8)	2 (9.1)	>.99
Asthma	4 (10.8)	0 (0.0)	.29
COPD	1 (2.7)	0 (0.0)	>.99
Cigarette smoking	17 (45.9)	7 (31.8)	.41
Medications			
Statin	6 (16.2)	1 (4.5)	.24
β-Blocker	9 (24.3)	2 (9.1)	.18
ACE-I or ARB	8 (21.6)	2 (9.1)	.29
CCB	7 (18.9)	1 (4.5)	.24

Abbreviations: ACE-I, angiotensin-converting-enzyme inhibitor; ARB, angiotensin receptor blockers; BMI, body mass index (calculated as weight in kilograms divided by height in meters squared); BSA, body surface area; CCBs, calcium channel blockers; COPD, chronic obstructive pulmonary disease; RPP, rate pressure product; SBP, systolic blood pressure.

SI conversion factors: To convert creatinine to micromoles per liter, multiply by 88.4; hematocrit to proportion of 1.0, multiply by 0.01.

^a^ *P* values denote Fisher exact test, independent *t* test, and Mann-Whitney *U* test. Comorbidities were assessed at baseline among patients with COVID-19 and at the time of cardiovascular magnetic resonance among volunteers; all other clinical characteristics, including medications, were recorded at the time of cardiovascular magnetic resonance.

^b^ Mann-Whitney *U* test.

### CMR at Follow-Up

CMR was performed at a median (IQR) 292 (203-367) days after discharge, or approximately 10 months. Due to poor image quality or contraindications to stress CMR, 3 patients with COVID-19 were excluded, rendering 37 patients included. CMR findings of patients with COVID-19 and volunteers are presented in Table 3. Native T2 maps were not obtained for 2 patients with COVID-19 due to operator dependency. There were no differences in LV mass or volumes, global native T1, native T2, or ECV between COVID-19 and volunteer groups. Patients with COVID-19 had worse mean GLS (−17% [95% CI, −18% to −16%] vs −19 [95% CI, −20% to −18 %]; *P* = .003) and GCS (−16% [95% CI, −17% to −15%] vs −19 [95% CI, −20% to −18%]; *P* = .001) compared with volunteers. Multivariable linear regression showed that a history of COVID-19 had an independent association with GLS (COVID-19 β = 1.58%; *P* = .016) and GCS (COVID-19 β = 2.64%; *P* = .003) when adjusting for sex and age. Minimal LGE was found in 4 patients with COVID-19, of whom 2 individuals had LGE consistent with a prior myocardial infarction and 2 individuals had LGE consistent with prior myocarditis. Due to limited scarring (<1 segment), the patients were included in the analysis.

**Table 3.** CMR Findings

CMR finding^a^	Mean (95% CI)		*P* value^b^
	Patients with COVID-19 (n = 37)	Volunteers (n = 22)	
LVM, g	110 (100 to 121)	100 (89 to 112)	.21
LVM index, g/m^2^	53 (49 to 57)	53 (49 to 57)	.70^c^
LVEDV, ml	172 (160 to 185)	173 (152 to 193)	.97
LVEDV index, ml/m^2^	83 (78 to 88)	91 (83 to 99)	.11^c^
LVESV, ml	74 (66 to 83)	73 (62 to 84)	.87
LVESV index, ml/m^2^	36 (32 to 40)	39 (34 to 43)	.35^c^
LVSV, ml	98 (91 to 105)	99 (87 to 112)	.81
LVSV index, ml/m^2^	48 (44 to 51)	52 (47 to 57)	.09
LVEF, %	57 (55 to 60)	58 (55 to 61)	.87
Cardiac output, l/min	7.1 (6.5 to 7.6)	6.7 (5.9 to 7.5)	.47^c^
GLS, %	−17 (−18 to −16)	−19 (−20 to −18)	.003
GCS, %	−16 (−17 to −15)	−19 (−20 to −18)	.001
Native T1, ms	995 (986 to 1005)	984 (970 to 997)	.15
Native T2, ms	48 (47 to 49)^d^	49 (48 to 50)	.26
ECV, %	27 (26 to 28)	27 (26 to 28)	.66
Perfusion, mL/min/g			
Rest^e^	1.10 (0.97 to 1.23)	1.16 (0.97 to 1.35)^d^	.41^b^
Stress	2.80 (2.53 to 3.07)	3.43 (3.13 to 3.74)	.003
MPR	2.7 (2.4 to 3.0)	3.2 (2.7 to 3.6)^d^	.07

Abbreviations: CMR, cardiac magnetic resonance imaging; ECV, extracellular volume; GCS, global circumferential strain; GLS, global longitudinal strain; LVEDV, left ventricular end-diastolic volume; LVEF, left ventricular ejection fraction; LVESV, left ventricular end-systolic volume; LVM, left ventricular mass; LVSV, left ventricular stroke volume; MPR, myocardial perfusion reserve.

^a^ Volumes were indexed to body surface area according to the Mosteller formula.

^b^ *P* values denotes the independent *t* test except where indicated.

^c^ Mann-Whitney *U* test.

^d^ T2 maps were not acquired in 2 patients with COVID-19. Rest perfusion maps were excluded in 1 volunteer due to residual hyperemia. MPR was not calculated for 1 volunteer.

^e^ Rest perfusion is corrected for rate pressure product.

### Quantitative Myocardial Perfusion

**Figure 1.**
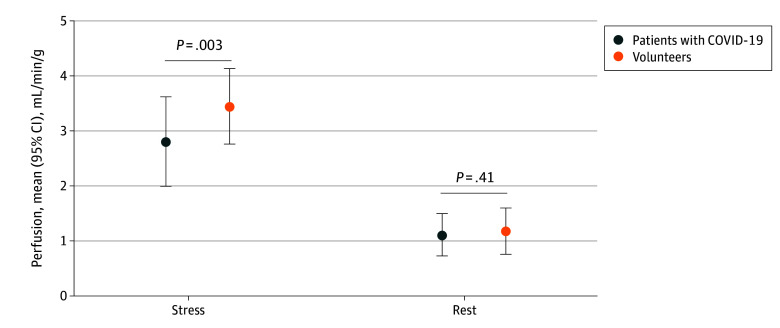
Stress and Rest Perfusion Values The figure shows the mean and 95% CI together with *P* values. Patients with COVID-19 had lower stress perfusion.

**Figure 2.**
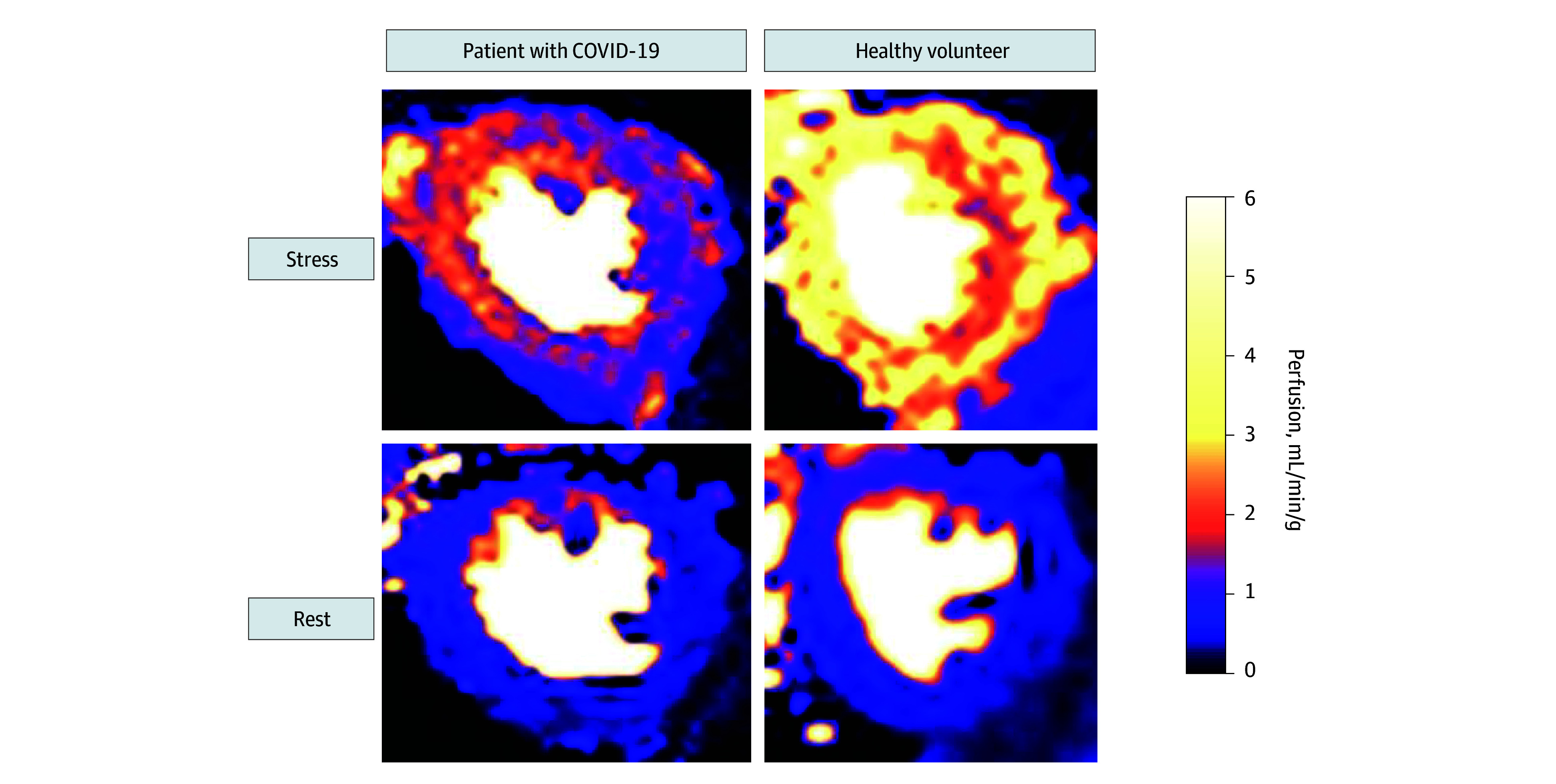
Stress and Rest Perfusion Imaging Midventricular perfusion maps are shown of a patient with COVID-19 with suspected microvascular dysfunction and a volunteer in stress and rest. Rest perfusion was comparable, while stress perfusion was globally reduced in the patient with COVID-19 compared with the volunteer.

Mean stress perfusion was lower in patients with COVID-19 compared with healthy volunteers (2.80 mL/min/g [95% CI, 2.53-3.07 mL/min/g] vs 3.43 mL/min/g [95% CI, 3.13-3.74 mL/min/g]; *P* = .003), but there was no difference in rest perfusion (Table 3; Figure 1). Mean MPR was lower in patients with COVID-19, but this difference was not statistically significant (2.7 [95% CI, 2.4-3.0] vs 3.2 [95% CI, 2.7-3.6]; *P* = .07). Rest perfusion maps were excluded in 1 volunteer due to residual hyperemia. Representative examples of perfusion maps of a patient with COVID-19 with suspected CMD and a volunteer are presented in Figure 2, showing lower stress perfusion in the patient with COVID-19. Multivariable linear regression showed that a history of COVID-19 had an independent association with stress perfusion when adjusting for sex and age (COVID-19 β = −0.63 mL/min/g; *P* = .007).

Stress and rest perfusion and MPR did not differ between female and male patients with COVID-19 or between patients with COVID-19 with or without a history of hypertension, hyperlipidemia, diabetes, or cigarette smoking (eTable in Supplement 1). Patients with COVID-19 reporting chest pressure at follow-up had a higher mean MPR compared with patients without chest pressure (3.3 [95% CI, 2.3-4.4] vs 2.5 [95% CI, 2.3-2.8]; *P* = .03), driven by nonsignificantly higher mean stress perfusion (3.1 mL/min/g [95% CI, 2.5-3.7 mL/min/g] vs 2.7 mL/min/g [95% CI, 2.4-3.0 mL/min/g]; *P* = .26) and lower rest perfusion (1.0 mL/min/g [95% CI, 0.8-1.2 mL/min/g] vs 1.1 mL/min/g [95% CI, 1.0-1.3 ml/min/g]; *P* = .76) levels. Otherwise, there were no differences in perfusion or MPR between patients with COVID-19 with or without chest pressure, dyspnea, or fatigue at follow-up (eTable in Supplement 1).

## Discussion

In this case-control study, we found that patients who were critically ill with COVID-19 from the first wave of the pandemic, with initially increased hsTnT levels, PAP, or both, exhibited lower stress perfusion 10 months later compared with volunteers without symptomatic ischemic heart disease; MPR was also lower for patients with COVID-19, but this difference was not statistically significant. Additionally, patients with COVID-19 had declined myocardial deformation, as shown by worse GLS and GCS, but volumes and LVEF did not differ. Minimal LGE was noted in 4 patients, without focal perfusion defects. Moreover, perfusion and MPR did not differ among patients with COVID-19 for those with vs without cardiovascular risk factors or symptoms at follow-up.

### Conventional CMR in Follow-Up of COVID-19

Perfusion defects and predominantly nonischemic LGE have been shown during acute COVID-19 or in early convalescence, with varying degrees of elevated native T1, native T2, and ECV, indicating inflammation, edema, or diffuse fibrosis.^32^ In particular, markers of systemic inflammation, native T1, native T2, and LGE were higher in early convalescence after severe COVID-19 compared with mild or moderate cases, potentially due to inflammation-mediated cardiac injury.^33^ Increased native T1 and native T2 in hospitalized patients with COVID-19 may normalize within 6 months despite persistent cardiopulmonary symptoms,^34,35^ while ECV may normalize within 1 year.^36^ Similarly, we found no differences in LV mass or volumes, native T1 or T2, or ECV at 10 months’ follow-up.

Despite long-term PASC symptoms, previous cardiopulmonary studies showed relatively minor objective findings.^37^ Similarly, despite persistent chest pressure and dyspnea as possible indicators of heart failure, cardiac volumes and LVEF did not differ between patients and volunteers in our study. In patients with COVID-19 with initial LV or right ventricular functional or structural alterations, CMR at 1 year showed improvements in strain and symptoms.^38^ However, impaired GLS with normal LVEF has been shown 6 months after intensive care for patients with COVID-19.^39^ Importantly, GLS may be used to evaluate cardiac improvement during follow-up of COVID-19 and to predict early mortality risk.^40^ We showed impaired GLS and GCS at 10 months after intensive care associated with COVID-19, likewise indicating some degree of persistent cardiac dysfunction.

### CMD and Perfusion Imaging in PASC

CMD may cause chest pain and dyspnea and may be identified with perfusion imaging. CMD has been shown in early convalescence after mild COVID-19, together with persistent cardiovascular symptoms, as indicated by increased rest perfusion or reduced stress perfusion and MPR.^41,42^ Long-term follow-up with adenosine stress perfusion CMR in mild to moderate initial COVID-19 with persistent symptoms^43,44^ and severe COVID-19 even requiring intensive care^45,46^ have shown varying results. Alternative findings include focal inducible perfusion deficits or LGE with myocardial infarction or nonischemic patterns^45^ and mild myocarditis-like injury and signs of ischemic heart disease in many patients with no prior history of ischemic heart disease.^46^ Our study showed reduced stress perfusion and a nonsignificantly lower MPR 10 months after severe COVID-19.

Quantitative CMR perfusion mapping is validated against positron emission tomography (PET),^47^ which is the reference standard for noninvasive quantitative perfusion imaging.^48^ However, PET lacks the broader differential diagnostic capabilities of multiparametric CMR, which we used. Previous PET studies have indicated CMD in PASC by reduced stress perfusion and MPR at 5 months in hospitalized and nonhospitalized patients with symptoms.^49^ At 11 months’ follow-up, combining PET with computed tomography to exclude coronary artery disease, MPR was decreased due to elevated rest perfusion, while stress perfusion was unchanged.^50^ Importantly, reduced stress perfusion and MPR, identified via PET, were shown in middle-aged, symptomatic males with cardiovascular risk factors at 6 months.^51^ This was associated with increased risk of major adverse cardiovascular events, including death, at 10 months.^51^ MPR was particularly impaired after severe COVID-19, especially in patients treated in the intensive care unit.^52^ Therefore, it is noteworthy that our study similarly found reduced stress perfusion and persistent cardiac symptoms in a predominantly male cohort of patients who were initially critically ill.

### CMD and COVID-19, Comorbidities, and Critical Illness

Variability in previous findings may be attributed to differences in initial disease severity, the presence of cardiopulmonary symptoms at follow-up, and the timing and methodology of imaging.^53^ Surprisingly, most earlier studies reported CMD predominantly in younger female patients with initial mild or moderate COVID-19. Our mostly male middle-aged patients with COVID-19 had a relatively low prevalence of known hypertension, type 2 diabetes, or hyperlipidemia despite greater weight and body mass index compared with volunteers. COVID-19 contributed to the differences in stress perfusion, GLS, and GCS. CMD is associated with obesity as determined by CMR^54^ and metabolic syndrome, diabetes, and hypertension as determined by PET.^55^ Cardiovascular risk factors associated with CMD are also associated with severe COVID-19,^14^ complicating efforts to ascertain whether CMD results from severe COVID-19 or represent a preexisting condition captured at follow-up.^56^ Importantly, CMD is also associated with sepsis,^57^ pneumonia, and acute respiratory distress syndrome.^14^ Microvascular dysfunction across vascular beds in patients with critical illness in intensive care units with various underlying conditions has also been shown acutely; however, long-term follow-up is largely lacking.^58^ Therefore, the underlying pathogenesis of the impaired stress perfusion as an indication of CMD in this study may be complex and multifactorial. Although we did not study treatment outcomes in this study, our findings may inspire therapeutic aspects to be included in future studies.

### Limitations

This study has several limitations, including its small sample size and single-center design. Our study used multiparametric CMR, including quantitative stress perfusion mapping, in a unique cohort of the first wave of patients who were critically ill with COVID-19 exhibiting long-term persistent cardiac symptoms. Patients were compared with volunteers of similar age and sex. Little long-term data on CMD in this patient category exist. Although generalizability to PASC in general may be limited, our results may be applicable for patients newly critically ill with COVID-19 who are unvaccinated or not optimally treated. Symptoms such as dyspnea, chest pressure, and fatigue may arise from respiratory or cardiovascular impairment,^35^ but our study does not allow assessment of pulmonary contribution. Patients were recruited prospectively, and given that prior studies indicate normalization of CMR parameters at 6 to 12 months of follow-up, the reduced stress perfusion and impaired strain we found may represent residual outcomes associated with initially more severe cardiac involvement. Because baseline CMR was not performed, such improvement over time could not be evaluated in this patient cohort.

## Conclusions

In this case-control study of patients with COVID-19 and healthy volunteers, patients exhibited long-term reduced stress perfusion indicating CMD and declined LV function by GLS and GCS. Lack of variation in stress perfusion between patients with and without cardiovascular risk factors may suggest CMD associated with severe COVID-19, warranting further investigation to elucidate mechanisms and guide potential therapeutic interventions.

## Notes

Supplement 1.
eAppendix.
**eTable.** Perfusion and MPR subgroup analysis

Supplement 2.
Data Sharing Statement
