# Errors in Results

**DOI:** 10.1001/jamanetworkopen.2025.27962

**Published:** 2025-07-22

In the Original Investigation titled “Long-Term Coronary Microvascular and Cardiac Dysfunction After Severe COVID-19 Hospitalization,”^1^ published June 9, 2025, incorrect β values were given in the Results section for the association between history of COVID-19 and global longitudinal strain (β reported as −0.32% should have been 1.58%), global circumferential strain (value reported as −0.40% should have been 2.64%), and stress perfusion (value reported as 0.37 mL/min/g should have been −0.63 mL/min/g). The article has been corrected.^1^

## References

[1] Steffen Johansson R, Loewenstein D, Lodin K, . Long-term coronary microvascular and cardiac dysfunction after severe COVID-19 hospitalization. JAMA Netw Open. 2025;8(6):e2514411. doi:10.1001/jamanetworkopen.2025.14411 40489109 PMC12150193

