# Supplementary material for: Long-Term Coronary Microvascular and Cardiac Dysfunction After Severe COVID-19 Hospitalization
Source: JAMA Netw Open. 2025 Jun 9;8(6):e2514411. doi: 10.1001/jamanetworkopen.2025.14411 (PMC12150193; doi:10.1001/jamanetworkopen.2025.14411)
Supplement: Supplement 1. — eAppendix. eTable. Perfusion and MPR subgroup analysis [file jamanetwopen-e2514411-s001.pdf]

Supplemental Online Content

Steffen Johansson R, Loewenstein D, Lodin K, et al. Long-term coronary microvascular and cardiac dysfunction after severe COVID-19 hospitalization. *JAMA Netw Open*. 2025;8(6):e2514411. doi:10.1001/jamanetworkopen.2025.14411

eAppendix.
eTable. Perfusion and MPR subgroup analysis

This supplemental material has been provided by the authors to give readers additional information about their work.

© 2025 Steffen Johansson R et al. JAMA Network Open.

\\USER\Kliniska hjartan\Hjärtprotokollet\COVID 6m\Thorax cor sag tra
TA: 0:42 PM: REF Voxel size: 1.8×1.8×6.0 mmPAT: 2 Rel. SNR: 1.00 : tfi

Properties

Prio recon	Off
Load images to viewer	On
Inline movie	Off
Auto store images	On
Load images to stamp segments On Load images to graphic segments On Auto open inline display	On
Auto close inline display	On
Start measurement without further preparation	Off
Wait for user to start	Off
Start measurements	Single measurement

Routine

Slice group	1
Slices	50
Dist. factor	0 %
Position	L22.3 P45.2 H13.6 mm
Orientation	Transversal
Phase enc. dir.	A >> P
Slice group	2
Slices	40
Dist. factor	0 %
Position	L30.0 P22.3 H0.0 mm
Orientation	Sagittal
Phase enc. dir.	A >> P
Slice group	3
Slices	35
Dist. factor	0 %
Position	R4.8 P34.2 H4.8 mm
Orientation	Coronal
Phase enc. dir.	R >> L
AutoAlign	---
Phase oversampling	0 %
FoV read	450 mm
FoV phase	100.0 %
Slice thickness	6.0 mm
TR	333.09 ms
TE	1.1 ms
Averages	1
Concatenations	125
Filter	Distortion Corr.(2D), Prescan Normalize
Coil elements	BO1-3;SP1-3

Contrast - Common

TR	333.09 ms
TE	1.1 ms
TD	0 ms
Magn. preparation	None
Flip angle	69 deg
Fat suppr.	None
Wrap-up Magn.	Restore

Contrast - Dynamic

Averages	1
Averaging mode	Short term
Reconstruction	Magnitude
Measurements	1
Multiple series	Each measurement

Resolution - Common

FoV read	450 mm
FoV phase	100.0 %
Slice thickness	6.0 mm
Base resolution	256
Phase resolution	66 %
Phase partial Fourier	Off
Trajectory	Cartesian
Interpolation	Off

Resolution - iPAT

PAT mode	GRAPPA
Accel. factor PE	2
Ref. lines PE	24
Reference scan mode	Integrated

Resolution - Filter Image

Image Filter	Off
Distortion Corr.	On
Mode	2D
Unfiltered images	Off
Prescan Normalize	On
Unfiltered images	Off
Normalize	Off
B1 filter	Off

Resolution - Filter Rawdata

Raw filter	Off
Elliptical filter	Off
POCS	Off

Geometry - Common

Slice group	1
Slices	50
Dist. factor	0 %
Position	L22.3 P45.2 H13.6 mm
Orientation	Transversal
Phase enc. dir.	A >> P
Slice group	2
Slices	40
Dist. factor	0 %
Position	L30.0 P22.3 H0.0 mm
Orientation	Sagittal
Phase enc. dir.	A >> P
Slice group	3
Slices	35
Dist. factor	0 %
Position	R4.8 P34.2 H4.8 mm
Orientation	Coronal
Phase enc. dir.	R >> L
FoV read	450 mm
FoV phase	100.0 %
Slice thickness	6.0 mm
TR	333.09 ms
Multi-slice mode	Sequential
Series	Descending
Concatenations	125

Geometry - AutoAlign

Slice group	1
Position	L22.3 P45.2 H13.6 mm

© 2025 Steffen Johansson R et al. JAMA Network Open.

Geometry - AutoAlign

Orientation	Transversal
Phase enc. dir.	A >> P
Slice group	2
Position	L30.0 P22.3 H0.0 mm
Orientation	Sagittal
Phase enc. dir.	A >> P
Slice group	3
Position	R4.8 P34.2 H4.8 mm
Orientation	Coronal
Phase enc. dir.	R >> L
AutoAlign	---
Initial Position	Isocenter
Phase	0.0 mm
Read	0.0 mm
Shift	0.0 mm
Initial Rotation	0.00 deg
Initial Orientation	Transversal

Geometry - Saturation

Fat suppr.	None
Wrap-up Magn.	Restore
Special sat.	None

Geometry - Navigator

Geometry - Tim Planning Suite

Set-n-Go Protocol	Off
Table position	H
Table position	0 mm
Inline Composing	Off

System - Miscellaneous

Positioning mode	REF
Table position	H
Table position	0 mm
MSMA	S - C - T
Sagittal	R >> L
Coronal	A >> P
Transversal	F >> H
Coil Combine Mode	Adaptive Combine
Save uncombined	Off
Matrix Optimization	Off
Coil Focus	Flat
AutoAlign	---
Coil Select Mode	Default

System - Adjustments

B0 Shim mode	Tune up
Adjust with body coil	Off
Confirm freq. adjustment	Off
Assume Dominant Fat	Off
Assume Silicone	Off
Adjustment Tolerance	Auto

System - Adjust Volume

Position	Isocenter
Orientation	Transversal
Rotation	0.00 deg
A >> P	263 mm
R >> L	350 mm
F >> H	350 mm
Reset	Off

System - Tx/Rx

Frequency 1H	63.672141 MHz
Correction factor	1
Gain	High
Img. Scale Cor.	1.000
Reset	Off
? Ref. amplitude 1H	0.000 V

Physio - Signal1

1st Signal/Mode	None
TR	333.09 ms
Concatenations	125
Segments	96

Physio - Cardiac

Tagging	None
Magn. preparation	None
Fat suppr.	None
Dark blood	Off
FoV read	450 mm
FoV phase	100.0 %
Phase resolution	66 %
Cine	Off
Trajectory	Cartesian
Dummy heartbeats	0

Physio - PACE

Resp. control	Off
Concatenations	125

Inline - Common

Subtract	Off
Measurements	1
StdDev	Off
Save original images	On

Inline - Cardiac

Inline Evaluation	Off
Magn. preparation	None
Contrasts	1
TE	1.1 ms
TR	333.09 ms
Save original images	On

Inline - MIP

MIP-Sag	Off
MIP-Cor	Off
MIP-Tra	Off
MIP-Time	Off
Save original images	On

Inline - Composing

Inline Composing	Off
Distortion Corr.	On
Mode	2D
Unfiltered images	Off

Sequence - Part 1

Introduction	Off
Dimension	2D
Reordering	Linear
Asymmetric echo	Weak
Contrasts	1
Optimization	Min. TE

© 2025 Steffen Johansson R et al. JAMA Network Open.

Sequence - Part 1

Multi-slice mode	Sequential
Echo spacing	2.6 ms
Sequence type	Trufi
Bandwidth	1149 Hz/Px

Sequence - Part 2

Define	Shots
Shots per slice	1
Segments	96
Trufi delta freq.	0 Hz
RF pulse type	Fast
Gradient mode	Fast
Excitation	Slice-sel.
Flip angle mode	Constant
Cine	Off

Sequence - Assistant

Mode	Min flip angle
Min flip angle	50 deg
Allowed delay	0 s

© 2025 Steffen Johansson R et al. JAMA Network Open.

\\USER\Kliniska hjartan\Hjärtprotokollet\COVID 6m\PREFUL_FLASH_cor_dx
TA: 1:38 PM: ISO Voxel size: 2.3×2.3×15.0 mmPAT: 2 Rel. SNR: 1.00 : tfl_r

Properties

Prio recon	Off
Load images to viewer	On
Inline movie	Off
Auto store images	On
Load images to stamp segments	On Load
images to graphic segments	On Auto
open inline display	Off
Auto close inline display	Off
Start measurement without further preparation	Off
Wait for user to start	Off
Start measurements	Single measurement

Routine

Slice group	1
Slices	1
Dist. factor	0 %
Position	R57.0 P60.4 H0.0 mm
Orientation	Coronal
Phase enc. dir.	R >> L
AutoAlign	---
Phase oversampling	0 %
FoV read	450 mm
FoV phase	100.0 %
Slice thickness	15.0 mm
TR	289.44 ms
TE	1.73 ms
Averages	1
Concatenations	1
Filter	Distortion Corr.(2D), Prescan Normalize
Coil elements	BO1-3;SP1-4

Contrast - Common

TR	289.44 ms
TE	1.73 ms
Magn. preparation	None
Flip angle	5 deg
Fat suppr.	None
Wrap-up Magn.	None

Contrast - Dynamic

Averages	1
Averaging mode	Short term
Reconstruction	Magnitude
Measurements	200
Pause after meas.	0.2 s
Multiple series	Off

Resolution - Common

FoV read	450 mm
FoV phase	100.0 %
Slice thickness	15.0 mm
Base resolution	192
Phase resolution	100 %
Phase partial Fourier	6/8
Trajectory	Cartesian
Interpolation	Off

Resolution - iPAT

PAT mode	GRAPPA
Accel. factor PE	2
Ref. lines PE	32
Reference scan mode	GRE/separate

Resolution - Filter Image

Image Filter	Off
Distortion Corr.	On
Mode	2D
Unfiltered images	Off
Prescan Normalize	On
Unfiltered images	Off
Normalize	Off
B1 filter	Off

Resolution - Filter Rawdata

Raw filter	Off
Elliptical filter	Off
POCS	Off

Geometry - Common

Slice group	1
Slices	1
Dist. factor	0 %
Position	R57.0 P60.4 H0.0 mm
Orientation	Coronal
Phase enc. dir.	R >> L
FoV read	450 mm
FoV phase	100.0 %
Slice thickness	15.0 mm
TR	289.44 ms
Multi-slice mode	Sequential
Series	Descending
Concatenations	1

Geometry - AutoAlign

Slice group	1
Position	R57.0 P60.4 H0.0 mm
Orientation	Coronal
Phase enc. dir.	R >> L
AutoAlign	---
Initial Position	R57.0 P60.4 H0.0
Phase	-57.0 mm
Read	0.0 mm
Shift	60.4 mm
Initial Rotation	0.00 deg
Initial Orientation	Coronal

Geometry - Saturation

Fat suppr.	None
Wrap-up Magn.	None
Special sat.	None

Geometry - Navigator

Geometry - Tim Planning Suite

Set-n-Go Protocol	Off
Table position	H
Table position	0 mm
Inline Composing	Off

© 2025 Steffen Johansson R et al. JAMA Network Open.

System - Miscellaneous

Positioning mode	ISO
Table position	H
Table position	0 mm
MSMA	S - C - T
Sagittal	R >> L
Coronal	A >> P
Transversal	H >> F
Coil Combine Mode	Adaptive Combine
Save uncombined	Off
Matrix Optimization	Off
Coil Focus	Flat
AutoAlign	---
Coil Select Mode	Off - All

System - Adjustments

B0 Shim mode	Standard
Adjust with body coil	Off
Confirm freq. adjustment	Off
Assume Dominant Fat	Off
Assume Silicone	Off
Adjustment Tolerance	Auto

System - Adjust Volume

Position	R57.0 P60.4 H0.0 mm
Orientation	Coronal
Rotation	0.00 deg
R >> L	450 mm
F >> H	450 mm
A >> P	15 mm
Reset	Off

System - Tx/Rx

Frequency 1H	63.672141 MHz
Correction factor	1
Gain	High
Img. Scale Cor.	25.000
Reset	Off
? Ref. amplitude 1H	0.000 V

Physio - Signal1

1st Signal/Mode	None
TR	289.44 ms
Concatenations	1
Segments	96

Physio - Cardiac

Tagging	None
Magn. preparation	None
Fat suppr.	None
Dark blood	Off
FoV read	450 mm
FoV phase	100.0 %
Phase resolution	100 %
Cine	Off
Trajectory	Cartesian
Dummy heartbeats	0

Physio - PACE

Resp. control	Off
Concatenations	1

Inline - Common

Subtract	Off
Measurements	200
StdDev	Off
Save original images	On

Inline - Cardiac

Inline Evaluation	Off
Magn. preparation	None
Contrasts	1
TE	1.73 ms
TR	289.44 ms
Save original images	On

Inline - MIP

MIP-Sag	Off
MIP-Cor	Off
MIP-Tra	Off
MIP-Time	Off
Save original images	On

Inline - Composing

Inline Composing	Off
Distortion Corr.	On
Mode	2D
Unfiltered images	Off

Sequence - Part 1

Introduction	Off
Dimension	2D
Reordering	Centric
Asymmetric echo	Strong
Contrasts	1
Flow comp.	Slice/Read
Optimization	Min. TE TR
Multi-slice mode	Sequential
Echo spacing	4 ms
Sequence type	Gre
Bandwidth	501 Hz/Px

Sequence - Part 2

Define	Shots
Shots per slice	1
Segments	96
RF pulse type	Fast
Gradient mode	Fast
Excitation	Slice-sel.
Flip angle mode	Constant
RF spoiling	On
Phase Enc. Rewinder	On
Cine	Off

Sequence - Assistant

Mode	Off
Allowed delay	5 s

© 2025 Steffen Johansson R et al. JAMA Network Open.

\\USER\Kliniska hjartan\Hjärtprotokollet\COVID 6m\PREFUL_FLASH_sagL
TA: 1:38 PM: ISO Voxel size: 2.3×2.3×15.0 mmPAT: 2 Rel. SNR: 1.00 : tfl_r

Properties

Prio recon	Off
Load images to viewer	On
Inline movie	Off
Auto store images	On
Load images to stamp segments	On
Load images to graphic segments	On
Auto open inline display	Off
Auto close inline display	Off
Start measurement without further preparation	Off
Wait for user to start	Off
Start measurements	Single measurement

Routine

Slice group	1
Slices	1
Dist. factor	0 %
Position	L89.5 P59.3 H0.0 mm
Orientation	Sagittal
Phase enc. dir.	A >> P
AutoAlign	---
Phase oversampling	0 %
FoV read	450 mm
FoV phase	100.0 %
Slice thickness	15.0 mm
TR	289.44 ms
TE	1.73 ms
Averages	1
Concatenations	1
Filter	Distortion Corr.(2D), Prescan Normalize
Coil elements	BO1-3;SP1-4

Contrast - Common

TR	289.44 ms
TE	1.73 ms
Magn. preparation	None
Flip angle	5 deg
Fat suppr.	None
Wrap-up Magn.	None

Contrast - Dynamic

Averages	1
Averaging mode	Short term
Reconstruction	Magnitude
Measurements	200
Pause after meas.	0.2 s
Multiple series	Off

Resolution - Common

FoV read	450 mm
FoV phase	100.0 %
Slice thickness	15.0 mm
Base resolution	192
Phase resolution	100 %
Phase partial Fourier	6/8
Trajectory	Cartesian
Interpolation	Off

Resolution - iPAT

PAT mode	GRAPPA
Accel. factor PE	2
Ref. lines PE	32
Reference scan mode	GRE/separate

Resolution - Filter Image

Image Filter	Off
Distortion Corr.	On
Mode	2D
Unfiltered images	Off
Prescan Normalize	On
Unfiltered images	Off
Normalize	Off
B1 filter	Off

Resolution - Filter Rawdata

Raw filter	Off
Elliptical filter	Off
POCS	Off

Geometry - Common

Slice group	1
Slices	1
Dist. factor	0 %
Position	L89.5 P59.3 H0.0 mm
Orientation	Sagittal
Phase enc. dir.	A >> P
FoV read	450 mm
FoV phase	100.0 %
Slice thickness	15.0 mm
TR	289.44 ms
Multi-slice mode	Sequential
Series	Descending
Concatenations	1

Geometry - AutoAlign

Slice group	1
Position	L89.5 P59.3 H0.0 mm
Orientation	Sagittal
Phase enc. dir.	A >> P
AutoAlign	---
Initial Position	L89.5 P59.3 H0.0
Phase	59.3 mm
Read	0.0 mm
Shift	89.5 mm
Initial Rotation	0.00 deg
Initial Orientation	Sagittal

Geometry - Saturation

Fat suppr.	None
Wrap-up Magn.	None
Special sat.	None

Geometry - Navigator

Geometry - Tim Planning Suite

Set-n-Go Protocol	Off
Table position	H
Table position	0 mm
Inline Composing	Off

© 2025 Steffen Johansson R et al. JAMA Network Open.

System - Miscellaneous

Positioning mode	ISO
Table position	H
Table position	0 mm
MSMA	S - C - T
Sagittal	R >> L
Coronal	A >> P
Transversal	H >> F
Coil Combine Mode	Adaptive Combine
Save uncombined	Off
Matrix Optimization	Off
Coil Focus	Flat
AutoAlign	---
Coil Select Mode	Off - All

System - Adjustments

B0 Shim mode	Standard
Adjust with body coil	Off
Confirm freq. adjustment	Off
Assume Dominant Fat	Off
Assume Silicone	Off
Adjustment Tolerance	Auto

System - Adjust Volume

Position	L89.5 P59.3 H0.0 mm
Orientation	Sagittal
Rotation	0.00 deg
A >> P	450 mm
F >> H	450 mm
R >> L	15 mm
Reset	Off

System - Tx/Rx

Frequency 1H	63.672141 MHz
Correction factor	1
Gain	High
Img. Scale Cor.	25.000
Reset	Off
? Ref. amplitude 1H	0.000 V

Physio - Signal1

1st Signal/Mode	None
TR	289.44 ms
Concatenations	1
Segments	96

Physio - Cardiac

Tagging	None
Magn. preparation	None
Fat suppr.	None
Dark blood	Off
FoV read	450 mm
FoV phase	100.0 %
Phase resolution	100 %
Cine	Off
Trajectory	Cartesian
Dummy heartbeats	0

Physio - PACE

Resp. control	Off
Concatenations	1

Inline - Common

Subtract	Off
Measurements	200
StdDev	Off
Save original images	On

Inline - Cardiac

Inline Evaluation	Off
Magn. preparation	None
Contrasts	1
TE	1.73 ms
TR	289.44 ms
Save original images	On

Inline - MIP

MIP-Sag	Off
MIP-Cor	Off
MIP-Tra	Off
MIP-Time	Off
Save original images	On

Inline - Composing

Inline Composing	Off
Distortion Corr.	On
Mode	2D
Unfiltered images	Off

Sequence - Part 1

Introduction	Off
Dimension	2D
Reordering	Centric
Asymmetric echo	Strong
Contrasts	1
Flow comp.	Slice/Read
Optimization	Min. TE TR
Multi-slice mode	Sequential
Echo spacing	4 ms
Sequence type	Gre
Bandwidth	501 Hz/Px

Sequence - Part 2

Define	Shots
Shots per slice	1
Segments	96
RF pulse type	Fast
Gradient mode	Fast
Excitation	Slice-sel.
Flip angle mode	Constant
RF spoiling	On
Phase Enc. Rewinder	On
Cine	Off

Sequence - Assistant

Mode	Off
Allowed delay	5 s

© 2025 Steffen Johansson R et al. JAMA Network Open.

\\USER\Kliniska hjartan\Hjärtprotokollet\COVID 6m\PREFUL_FLASH_sagR
TA: 1:38 PM: ISO Voxel size: 2.3×2.3×15.0 mmPAT: 2 Rel. SNR: 1.00 : tfl_r

Properties

Prio recon	Off
Load images to viewer	On
Inline movie	Off
Auto store images	On
Load images to stamp segments	On Load
images to graphic segments	On Auto
open inline display	Off
Auto close inline display	Off
Start measurement without further preparation	Off
Wait for user to start	Off
Start measurements	Single measurement

Routine

Slice group	1
Slices	1
Dist. factor	0 %
Position	R57.0 P60.4 H0.0 mm
Orientation	Sagittal
Phase enc. dir.	A >> P
AutoAlign	---
Phase oversampling	0 %
FoV read	450 mm
FoV phase	100.0 %
Slice thickness	15.0 mm
TR	289.44 ms
TE	1.73 ms
Averages	1
Concatenations	1
Filter	Distortion Corr.(2D), Prescan Normalize
Coil elements	BO1-3;SP1-4

Contrast - Common

TR	289.44 ms
TE	1.73 ms
Magn. preparation	None
Flip angle	5 deg
Fat suppr.	None
Wrap-up Magn.	None

Contrast - Dynamic

Averages	1
Averaging mode	Short term
Reconstruction	Magnitude
Measurements	200
Pause after meas.	0.2 s
Multiple series	Off

Resolution - Common

FoV read	450 mm
FoV phase	100.0 %
Slice thickness	15.0 mm
Base resolution	192
Phase resolution	100 %
Phase partial Fourier	6/8
Trajectory	Cartesian
Interpolation	Off

Resolution - iPAT

PAT mode	GRAPPA
Accel. factor PE	2
Ref. lines PE	32
Reference scan mode	GRE/separate

Resolution - Filter Image

Image Filter	Off
Distortion Corr.	On
Mode	2D
Unfiltered images	Off
Prescan Normalize	On
Unfiltered images	Off
Normalize	Off
B1 filter	Off

Resolution - Filter Rawdata

Raw filter	Off
Elliptical filter	Off
POCS	Off

Geometry - Common

Slice group	1
Slices	1
Dist. factor	0 %
Position	R57.0 P60.4 H0.0 mm
Orientation	Sagittal
Phase enc. dir.	A >> P
FoV read	450 mm
FoV phase	100.0 %
Slice thickness	15.0 mm
TR	289.44 ms
Multi-slice mode	Sequential
Series	Descending
Concatenations	1

Geometry - AutoAlign

Slice group	1
Position	R57.0 P60.4 H0.0 mm
Orientation	Sagittal
Phase enc. dir.	A >> P
AutoAlign	---
Initial Position	R57.0 P60.4 H0.0
Phase	60.4 mm
Read	0.0 mm
Shift	-57.0 mm
Initial Rotation	0.00 deg
Initial Orientation	Sagittal

Geometry - Saturation

Fat suppr.	None
Wrap-up Magn.	None
Special sat.	None

Geometry - Navigator

Geometry - Tim Planning Suite

Set-n-Go Protocol	Off
Table position	H
Table position	0 mm
Inline Composing	Off

© 2025 Steffen Johansson R et al. JAMA Network Open.

System - Miscellaneous

Positioning mode	ISO
Table position	H
Table position	0 mm
MSMA	S - C - T
Sagittal	R >> L
Coronal	A >> P
Transversal	H >> F
Coil Combine Mode	Adaptive Combine
Save uncombined	Off
Matrix Optimization	Off
Coil Focus	Flat
AutoAlign	---
Coil Select Mode	Off - All

System - Adjustments

B0 Shim mode	Standard
Adjust with body coil	Off
Confirm freq. adjustment	Off
Assume Dominant Fat	Off
Assume Silicone	Off
Adjustment Tolerance	Auto

System - Adjust Volume

Position	R57.0 P60.4 H0.0 mm
Orientation	Sagittal
Rotation	0.00 deg
A >> P	450 mm
F >> H	450 mm
R >> L	15 mm
Reset	Off

System - Tx/Rx

Frequency 1H	63.672141 MHz
Correction factor	1
Gain	High
Img. Scale Cor.	25.000
Reset	Off
? Ref. amplitude 1H	0.000 V

Physio - Signal1

1st Signal/Mode	None
TR	289.44 ms
Concatenations	1
Segments	96

Physio - Cardiac

Tagging	None
Magn. preparation	None
Fat suppr.	None
Dark blood	Off
FoV read	450 mm
FoV phase	100.0 %
Phase resolution	100 %
Cine	Off
Trajectory	Cartesian
Dummy heartbeats	0

Physio - PACE

Resp. control	Off
Concatenations	1

Inline - Common

Subtract	Off

© 2025 Steffen Johansson R et al. JAMA Network Open.

Inline - Common

Measurements	200
StdDev	Off
Save original images	On

Inline - Cardiac

Inline Evaluation	Off
Magn. preparation	None
Contrasts	1
TE	1.73 ms
TR	289.44 ms
Save original images	On

Inline - MIP

MIP-Sag	Off
MIP-Cor	Off
MIP-Tra	Off
MIP-Time	Off
Save original images	On

Inline - Composing

Inline Composing	Off
Distortion Corr.	On
Mode	2D
Unfiltered images	Off

Sequence - Part 1

Introduction	Off
Dimension	2D
Reordering	Centric
Asymmetric echo	Strong
Contrasts	1
Flow comp.	Slice/Read
Optimization	Min. TE TR
Multi-slice mode	Sequential
Echo spacing	4 ms
Sequence type	Gre
Bandwidth	501 Hz/Px

Sequence - Part 2

Define	Shots
Shots per slice	1
Segments	96
RF pulse type	Fast
Gradient mode	Fast
Excitation	Slice-sel.
Flip angle mode	Constant
RF spoiling	On
Phase Enc. Rewinder	On
Cine	Off

Sequence - Assistant

Mode	Off
Allowed delay	5 s

\\USER\Kliniska hjartan\Hjärtprotokollet\COVID 6m\VLA NBH
TA: 1.0 s PM: REF Voxel size: 1.8×1.8×6.0 mmPAT: 2 Rel. SNR: 1.00 : tfi

Properties

Prio recon	Off
Load images to viewer	On
Inline movie	Off
Auto store images	On
Load images to stamp segments	On Load
images to graphic segments	On Auto
open inline display	Off
Auto close inline display	Off
Start measurement without further preparation	Off
Wait for user to start	On
Start measurements	Single measurement

Routine

Slice group	1
Slices	3
Dist. factor	0 %
Position	L23.4 P49.7 F22.4 mm
Orientation	C > S-33.4
Phase enc. dir.	L >> R
AutoAlign	---
Phase oversampling	0 %
FoV read	340 mm
FoV phase	81.3 %
Slice thickness	6.0 mm
TR	35.49 ms
TE	1.16 ms
Averages	1
Concatenations	1
Filter	Distortion Corr.(2D), Prescan Normalize
Coil elements	BO1-3;SP1-3

Contrast - Common

TR	35.49 ms
TE	1.16 ms
Magn. preparation	None
Flip angle	70 deg
Fat suppr.	None
Wrap-up Magn.	Restore

Contrast - Dynamic

Averages	1
Averaging mode	Short term
Reconstruction	Magnitude
Measurements	1
Multiple series	Off

Resolution - Common

FoV read	340 mm
FoV phase	81.3 %
Slice thickness	6.0 mm
Base resolution	192
Phase resolution	100 %
Phase partial Fourier	Off
Trajectory	Cartesian
View sharing	Off
Interpolation	Off

Resolution - iPAT

PAT mode	GRAPPA
Accel. factor PE	2
Ref. lines PE	44
Reference scan mode	Integrated

Resolution - Filter Image

Image Filter	Off
Distortion Corr.	On
Mode	2D
Unfiltered images	Off
Prescan Normalize	On
Unfiltered images	Off
Normalize	Off
B1 filter	Off

Resolution - Filter Rawdata

Raw filter	Off
Elliptical filter	Off
POCS	Off

Geometry - Common

Slice group	1
Slices	3
Dist. factor	0 %
Position	L23.4 P49.7 F22.4 mm
Orientation	C > S-33.4
Phase enc. dir.	L >> R
FoV read	340 mm
FoV phase	81.3 %
Slice thickness	6.0 mm
TR	35.49 ms
Multi-slice mode	Sequential
Series	Interl. in B.-h.
Concatenations	1

Geometry - AutoAlign

Slice group	1
Position	L23.4 P49.7 F22.4 mm
Orientation	C > S-33.4
Phase enc. dir.	L >> R
AutoAlign	---
Initial Position	L23.4 P49.7 F22.4
Phase	7.9 mm
Read	-22.4 mm
Shift	54.4 mm
Initial Rotation	-180.00 deg
Initial Orientation	C > S
C > S	-33.4
> T	0.0

Geometry - Saturation

Fat suppr.	None
Wrap-up Magn.	Restore
Special sat.	None

Geometry - Navigator

Geometry - Tim Planning Suite

Set-n-Go Protocol	Off
Table position	H

© 2025 Steffen Johansson R et al. JAMA Network Open.

Geometry - Tim Planning Suite

Table position	0 mm
Inline Composing	Off

System - Miscellaneous

Positioning mode	REF
Table position	H
Table position	0 mm
MSMA	S - C - T
Sagittal	R >> L
Coronal	A >> P
Transversal	F >> H
Coil Combine Mode	Sum of Squares
Save uncombined	Off
Matrix Optimization	Off
Coil Focus	Flat
AutoAlign	---
Coil Select Mode	Default

System - Adjustments

B0 Shim mode	Tune up
Adjust with body coil	Off
Confirm freq. adjustment	Off
Assume Dominant Fat	Off
Assume Silicone	Off
Adjustment Tolerance	Auto

System - Adjust Volume

Position	Isocenter
Orientation	Transversal
Rotation	0.00 deg
A >> P	263 mm
R >> L	350 mm
F >> H	350 mm
Reset	Off

System - Tx/Rx

Frequency 1H	63.672141 MHz
Correction factor	1
Gain	High
Img. Scale Cor.	1.000
Reset	Off
? Ref. amplitude 1H	0.000 V

Physio - Signal1

1st Signal/Mode	None
TR	35.49 ms
Concatenations	1
Segments	13

Physio - Cardiac

Tagging	None
Magn. preparation	None
Fat suppr.	None
Dark blood	Off
FoV read	340 mm
FoV phase	81.3 %
Phase resolution	100 %
Cine	On
Trajectory	Cartesian
View sharing	Off
Dummy heartbeats	0

Physio - PACE

Resp. control	Breath-hold
Concatenations	1

Inline - Common

Subtract	Off
Measurements	1
StdDev	Off
Save original images	On

Inline - Cardiac

Inline Evaluation	Off
Magn. preparation	None
Contrasts	1
TE	1.16 ms
TR	35.49 ms
Save original images	On

Inline - MIP

MIP-Sag	Off
MIP-Cor	Off
MIP-Tra	Off
MIP-Time	Off
Save original images	On

Inline - Composing

Inline Composing	Off
Distortion Corr.	On
Mode	2D
Unfiltered images	Off

Sequence - Part 1

Introduction	Off
Dimension	2D
Reordering	Linear
Asymmetric echo	Weak
Contrasts	1
Optimization	Min. TE TR
Multi-slice mode	Sequential
Echo spacing	2.7 ms
Sequence type	Trufi
Bandwidth	930 Hz/Px

Sequence - Part 2

Define	Segments
Segments	13
Trufi delta freq.	0 Hz
RF pulse type	Fast
Gradient mode	Fast
Excitation	Slice-sel.
Flip angle mode	Constant
Cine	On

Sequence - Assistant

Mode	Min flip angle
Min flip angle	50 deg
Allowed delay	0 s

© 2025 Steffen Johansson R et al. JAMA Network Open.

\\USER\Kliniska hjartan\Hjärtprotokollet\COVID 6m\SA scout
TA: 3.4 s PM: FIX Voxel size: 4.2×4.2×8.0 mmPAT: 3 Rel. SNR: 1.00 : tfi

Properties

Prio recon	Off
Load images to viewer	On
Inline movie	On
Auto store images	On
Load images to stamp segments	On Load
images to graphic segments	On Auto
open inline display	Off
Auto close inline display	Off
Start measurement without further preparation	Off
Wait for user to start	Off
Start measurements	Single measurement

Routine

Slice group	1
Slices	2
Dist. factor	250 %
Position	L84.6 P26.8 F43.8 mm
Orientation	T > S40.1 > C-28.8
Phase enc. dir.	A >> P
AutoAlign	---
Phase oversampling	0 %
FoV read	400 mm
FoV phase	89.6 %
Slice thickness	8.0 mm
TR	47.60 ms
TE	1.07 ms
Averages	1
Concatenations	1
Filter	Distortion Corr.(2D), Prescan Normalize
Coil elements	BO1-3;SP1-3

Contrast - Common

TR	47.60 ms
TE	1.07 ms
Magn. preparation	None
Flip angle	67 deg
Fat suppr.	None
Wrap-up Magn.	Restore

Contrast - Dynamic

Averages	1
Averaging mode	Short term
Reconstruction	Magnitude
Measurements	1
Multiple series	Off

Resolution - Common

FoV read	400 mm
FoV phase	89.6 %
Slice thickness	8.0 mm
Base resolution	96
Phase resolution	100 %
Phase partial Fourier	Off
Trajectory	Cartesian
View sharing	Off
Interpolation	Off

Resolution - iPAT

PAT mode	GRAPPA
Accel. factor PE	3
Ref. lines PE	36
Reference scan mode	Integrated

Resolution - Filter Image

Image Filter	Off
Distortion Corr.	On
Mode	2D
Unfiltered images	Off
Prescan Normalize	On
Unfiltered images	Off
Normalize	Off
B1 filter	Off

Resolution - Filter Rawdata

Raw filter	Off
Elliptical filter	Off
POCS	Off

Geometry - Common

Slice group	1
Slices	2
Dist. factor	250 %
Position	L84.6 P26.8 F43.8 mm
Orientation	T > S40.1 > C-28.8
Phase enc. dir.	A >> P
FoV read	400 mm
FoV phase	89.6 %
Slice thickness	8.0 mm
TR	47.60 ms
Multi-slice mode	Sequential
Series	Base To Apex
Concatenations	1

Geometry - AutoAlign

Slice group	1
Position	L84.6 P26.8 F43.8 mm
Orientation	T > S40.1 > C-28.8
Phase enc. dir.	A >> P
AutoAlign	---
Initial Position	L84.6 P26.8 F43.8
Phase	65.9 mm
Read	-36.5 mm
Shift	-64.3 mm
Initial Rotation	22.08 deg
Initial Orientation	T > S
T > S	40.1
>C	-28.8

Geometry - Saturation

Fat suppr.	None
Wrap-up Magn.	Restore
Special sat.	None

Geometry - Navigator

Geometry - Tim Planning Suite

Set-n-Go Protocol	Off
Table position	H

© 2025 Steffen Johansson R et al. JAMA Network Open.

Geometry - Tim Planning Suite

Table position	0 mm
Inline Composing	Off

System - Miscellaneous

Positioning mode	FIX
Table position	H
Table position	0 mm
MSMA	S - C - T
Sagittal	R >> L
Coronal	A >> P
Transversal	F >> H
Coil Combine Mode	Sum of Squares
Save uncombined	Off
Matrix Optimization	Off
Coil Focus	Flat
AutoAlign	---
Coil Select Mode	Default

System - Adjustments

B0 Shim mode	Tune up
Adjust with body coil	Off
Confirm freq. adjustment	Off
Assume Dominant Fat	Off
Assume Silicone	Off
Adjustment Tolerance	Auto

System - Adjust Volume

Position	Isocenter
Orientation	Transversal
Rotation	0.00 deg
A >> P	263 mm
R >> L	350 mm
F >> H	350 mm
Reset	Off

System - Tx/Rx

Frequency 1H	63.672141 MHz
Correction factor	1
Gain	High
Img. Scale Cor.	1.000
Reset	Off
? Ref. amplitude 1H	0.000 V

Physio - Signal1

1st Signal/Mode	ECG/Retro
Average cycle	430 ± 159 ms
Average cycle	No Signal ms
Calculated phases	25
TR	47.60 ms
Concatenations	1
Segments	20
Arrhythmia detection	None

Physio - Cardiac

Tagging	None
Magn. preparation	None
Fat suppr.	None
Dark blood	Off
FoV read	400 mm
FoV phase	89.6 %
Phase resolution	100 %
Cine	On
Trajectory	Cartesian
View sharing	Off

Physio - Cardiac

Dummy heartbeats	1

Physio - PACE

Resp. control	Breath-hold
Concatenations	1

Inline - Common

Subtract	Off
Measurements	1
StdDev	Off
Save original images	On

Inline - Cardiac

Inline Evaluation	Off
Magn. preparation	None
Contrasts	1
TE	1.07 ms
TR	47.60 ms
Save original images	On

Inline - MIP

MIP-Sag	Off
MIP-Cor	Off
MIP-Tra	Off
MIP-Time	Off
Save original images	On

Inline - Composing

Inline Composing	Off
Distortion Corr.	On
Mode	2D
Unfiltered images	Off

Sequence - Part 1

Introduction	Off
Dimension	2D
Reordering	Linear
Asymmetric echo	Weak
Contrasts	1
Optimization	Min. TE TR
Multi-slice mode	Sequential
Echo spacing	2.4 ms
Sequence type	Trufi
Bandwidth	914 Hz/Px

Sequence - Part 2

Define	Segments
Segments	20
Trufi delta freq.	0 Hz
RF pulse type	Fast
Gradient mode	Fast
Excitation	Slice-sel.
Flip angle mode	Constant
Cine	On

Sequence - Assistant

Mode	Min flip angle
Min flip angle	50 deg
Allowed delay	0 s

© 2025 Steffen Johansson R et al. JAMA Network Open.

\\USER\Kliniska hjartan\Hjärtprotokollet\COVID 6m\cine_-4ch_no scout
TA: 3.9 s PM: FIX Voxel size: 1.5×1.5×8.0 mmPAT: 2 Rel. SNR: 1.00 : tfi

Properties

Prio recon	Off
Load images to viewer	On
Inline movie	On
Auto store images	On
Load images to stamp segments	On Load
images to graphic segments	On Auto
open inline display	Off
Auto close inline display	Off
Start measurement without further preparation	Off
Wait for user to start	Off
Start measurements	Single measurement

Routine

Slice group	1
Slices	1
Dist. factor	20 %
Position	Isocenter
Orientation	Transversal
Phase enc. dir.	A >> P
AutoAlign	---
Phase oversampling	0 %
FoV read	380 mm
FoV phase	84.4 %
Slice thickness	8.0 mm
TR	36.14 ms
TE	1.16 ms
Averages	1
Concatenations	1
Filter	Distortion Corr.(2D), Prescan Normalize
Coil elements	BO1-3;SP1-4

Contrast - Common

TR	36.14 ms
TE	1.16 ms
Magn. preparation	None
Flip angle	68 deg
Fat suppr.	None
Wrap-up Magn.	Restore

Contrast - Dynamic

Averages	1
Averaging mode	Short term
Reconstruction	Magnitude
Measurements	1
Multiple series	Off

Resolution - Common

FoV read	380 mm
FoV phase	84.4 %
Slice thickness	8.0 mm
Base resolution	256
Phase resolution	66 %
Phase partial Fourier	Off
Trajectory	Cartesian
View sharing	Off
Interpolation	Off

Resolution - iPAT

PAT mode	GRAPPA
Accel. factor PE	2
Ref. lines PE	44
Reference scan mode	Integrated

Resolution - Filter Image

Image Filter	Off
Distortion Corr.	On
Mode	2D
Unfiltered images	Off
Prescan Normalize	On
Unfiltered images	Off
Normalize	Off
B1 filter	Off

Resolution - Filter Rawdata

Raw filter	Off
Elliptical filter	Off
POCS	Off

Geometry - Common

Slice group	1
Slices	1
Dist. factor	20 %
Position	Isocenter
Orientation	Transversal
Phase enc. dir.	A >> P
FoV read	380 mm
FoV phase	84.4 %
Slice thickness	8.0 mm
TR	36.14 ms
Multi-slice mode	Sequential
Series	Interleaved
Concatenations	1

Geometry - AutoAlign

Slice group	1
Position	Isocenter
Orientation	Transversal
Phase enc. dir.	A >> P
AutoAlign	---
Initial Position	Isocenter
Phase	0.0 mm
Read	0.0 mm
Shift	0.0 mm
Initial Rotation	0.00 deg
Initial Orientation	Transversal

Geometry - Saturation

Fat suppr.	None
Wrap-up Magn.	Restore
Special sat.	None

Geometry - Navigator

Geometry - Tim Planning Suite

Set-n-Go Protocol	Off
Table position	H
Table position	0 mm
Inline Composing	Off

© 2025 Steffen Johansson R et al. JAMA Network Open.

System - Miscellaneous

Positioning mode	FIX
Table position	H
Table position	0 mm
MSMA	S - C - T
Sagittal	R >> L
Coronal	A >> P
Transversal	F >> H
Coil Combine Mode	Sum of Squares
Save uncombined	Off
Matrix Optimization	Off
Coil Focus	Flat
AutoAlign	---
Coil Select Mode	Default

System - Adjustments

B0 Shim mode	Tune up
Adjust with body coil	Off
Confirm freq. adjustment	Off
Assume Dominant Fat	Off
Assume Silicone	Off
Adjustment Tolerance	Auto

System - Adjust Volume

Position	Isocenter
Orientation	Transversal
Rotation	0.00 deg
A >> P	263 mm
R >> L	350 mm
F >> H	350 mm
Reset	Off

System - Tx/Rx

Frequency 1H	63.672141 MHz
Correction factor	1
Gain	High
Img. Scale Cor.	1.000
Reset	Off
? Ref. amplitude 1H	0.000 V

Physio - Signal1

1st Signal/Mode	ECG/Retro
Average cycle	430 ± 159 ms
Average cycle	No Signal ms
Calculated phases	25
TR	36.14 ms
Concatenations	1
Segments	13
Arrhythmia detection	None

Physio - Cardiac

Tagging	None
Magn. preparation	None
Fat suppr.	None
Dark blood	Off
FoV read	380 mm
FoV phase	84.4 %
Phase resolution	66 %
Cine	On
Trajectory	Cartesian
View sharing	Off
Dummy heartbeats	1

Physio - PACE

Concatenations	1

Inline - Common

Subtract	Off
Measurements	1
StdDev	Off
Save original images	On

Inline - Cardiac

Inline Evaluation	Off
Magn. preparation	None
Contrasts	1
TE	1.16 ms
TR	36.14 ms
Save original images	On

Inline - MIP

MIP-Sag	Off
MIP-Cor	Off
MIP-Tra	Off
MIP-Time	Off
Save original images	On

Inline - Composing

Inline Composing	Off
Distortion Corr.	On
Mode	2D
Unfiltered images	Off

Sequence - Part 1

Introduction	Off
Dimension	2D
Reordering	Linear
Asymmetric echo	Weak
Contrasts	1
Optimization	Min. TE TR
Multi-slice mode	Sequential
Echo spacing	2.8 ms
Sequence type	Trufi
Bandwidth	930 Hz/Px

Sequence - Part 2

Define	Segments
Segments	13
Trufi delta freq.	0 Hz
RF pulse type	Fast
Gradient mode	Fast
Excitation	Slice-sel.
Flip angle mode	Constant
Cine	On

Sequence - Assistant

Mode	Min flip angle
Min flip angle	50 deg
Allowed delay	0 s

© 2025 Steffen Johansson R et al. JAMA Network Open.

Resp. control	Off

© 2025 Steffen Johansson R et al. JAMA Network Open.

\\USER\Kliniska hjartan\Hjärtprotokollet\COVID 6m\SSFP_Perf_MBF_AI TEST
TA: 0:10 PM: REF Voxel size: 1.9×1.9×8.0 mmPAT: 3 Rel. SNR: 1.00 : tfi

Properties

Prio recon	Off
Load images to viewer	Off
Inline movie	Off
Auto store images	On
Load images to stamp segments	Off Load
images to graphic segments	On Auto
open inline display	Off
Auto close inline display	Off
Start measurement without further preparation	Off
Wait for user to start	On
Start measurements	Single measurement

Routine

Slice group	1
Slices	3
Dist. factor	100 %
Position	Isocenter
Orientation	Transversal
Phase enc. dir.	A >> P
AutoAlign	---
Phase oversampling	0 %
FoV read	360 mm
FoV phase	75.0 %
Slice thickness	8.0 mm
TR	142.00 ms
TE	1.04 ms
Averages	1
Concatenations	1
Filter	Distortion Corr.(2D)
Coil elements	BO1-3;SP3,4

Contrast - Common

TR	142.00 ms
TE	1.04 ms
Magn. preparation	Non-sel. SR perf
TI	105 ms
Flip angle	50 deg
Fat suppr.	Fat sat.
Wrap-up Magn.	None

Contrast - Dynamic

Averages	1
Averaging mode	Long term
Reconstruction	Magnitude
Measurements	12
Pause after meas. 1	0.0 s
Pause after meas. 2	0.0 s
Pause after meas. 3	0.0 s
Pause after meas. 4	0.0 s
Pause after meas. 5	0.0 s
Pause after meas. 6	0.0 s
Pause after meas. 7	0.0 s
Pause after meas. 8	0.0 s
Pause after meas. 9	0.0 s
Pause after meas. 10	0.0 s
Pause after meas. 11	0.0 s
Proton Dens. Maps	3
Multiple series	Off

Resolution - Common

FoV read	360 mm
FoV phase	75.0 %
Slice thickness	8.0 mm
Base resolution	192
Phase resolution	77 %
Phase partial Fourier	6/8
Trajectory	Cartesian
Interpolation	Off

Resolution - iPAT

PAT mode	GRAPPA
Accel. factor PE	3
Reference scan mode	T-PAT

Resolution - Filter Image

Image Filter	Off
Distortion Corr.	On
Mode	2D
Unfiltered images	Off
Prescan Normalize	Off
Normalize	Off
B1 filter	Off

Resolution - Filter Rawdata

Raw filter	Off
Elliptical filter	Off
POCS	Off

Geometry - Common

Slice group	1
Slices	3
Dist. factor	100 %
Position	Isocenter
Orientation	Transversal
Phase enc. dir.	A >> P
FoV read	360 mm
FoV phase	75.0 %
Slice thickness	8.0 mm
TR	142.00 ms
Multi-slice mode	Single shot
Series	Ascending
Concatenations	1

Geometry - AutoAlign

Slice group	1
Position	Isocenter
Orientation	Transversal
Phase enc. dir.	A >> P
AutoAlign	---
Initial Position	Isocenter
Phase	0.0 mm
Read	0.0 mm
Shift	0.0 mm
Initial Rotation	0.00 deg
Initial Orientation	Transversal

Geometry - Saturation

Fat suppr.	Fat sat.
Wrap-up Magn.	None
Special sat.	None

© 2025 Steffen Johansson R et al. JAMA Network Open.

Geometry - Navigator

Geometry - Tim Planning Suite

Set-n-Go Protocol	Off
Table position	H
Table position	0 mm
Inline Composing	Off

System - Miscellaneous

Positioning mode	REF
Table position	H
Table position	0 mm
MSMA	S - C - T
Sagittal	R >> L
Coronal	A >> P
Transversal	F >> H
Coil Combine Mode	Sum of Squares
Save uncombined	Off
Matrix Optimization	Off
Coil Focus	Flat
AutoAlign	---
Coil Select Mode	Default

System - Adjustments

B0 Shim mode	Cardiac
Adjust with body coil	On
Confirm freq. adjustment	Off
Assume Dominant Fat	Off
Assume Silicone	Off
Adjustment Tolerance	Auto

System - Adjust Volume

Position	Isocenter
Orientation	Transversal
Rotation	0.00 deg
A >> P	270 mm
R >> L	360 mm
F >> H	40 mm
Reset	Off

System - Tx/Rx

Frequency 1H	63.672141 MHz
Correction factor	1
Gain	High
Img. Scale Cor.	1.000
Reset	Off
? Ref. amplitude 1H	0.000 V

Physio - Signal1

1st Signal/Mode	ECG/Trigger
Average cycle	430 ± 159 ms
Average cycle	No Signal ms
Captured cycle	430 ± 159 ms
Acquisition window	491 ms
Trigger pulse	1
Trigger delay	0 ms
TR	142.00 ms
Concatenations	1
Segments	37
Phases	1
Adaptive Triggering	Off

Physio - Cardiac

Tagging	None

Physio - Cardiac

Magn. preparation	Non-sel. SR perf
TI	105 ms
Fat suppr.	Fat sat.
Dark blood	Off
FoV read	360 mm
FoV phase	75.0 %
Phase resolution	77 %
Cine	Off
Trajectory	Cartesian
Dummy heartbeats	0
Motion Correction	None

Physio - PACE

Resp. control	Off
Concatenations	1

Inline - Common

Subtract	Off
Measurements	12
StdDev	Off
Motion Correction	None
Save original images	On

Inline - Cardiac

Inline Evaluation	Off
Magn. preparation	Non-sel. SR perf
TE	1.04 ms
TR	142.00 ms
Motion Correction	None
Save original images	On

Inline - MIP

MIP-Sag	Off
MIP-Cor	Off
MIP-Tra	Off
MIP-Time	Off
Save original images	On

Inline - Composing

Inline Composing	Off
Distortion Corr.	On
Mode	2D
Unfiltered images	Off

Sequence - Part 1

Introduction	Off
Dimension	2D
Reordering	Linear
Asymmetric echo	Allowed
Optimization	Min. TE
Multi-slice mode	Single shot
Echo spacing	2.5 ms
Sequence type	Trufi
Bandwidth	1085 Hz/Px

Sequence - Part 2

Define	Shots
Shots per slice	1
EPI factor	1
Segments	37
Trufi delta freq.	0 Hz
RF pulse type	Fast
Gradient mode	Fast

© 2025 Steffen Johansson R et al. JAMA Network Open.

Sequence - Part 2

Excitation	Slice-sel.
Flip angle mode	Constant
Cine	Off

Sequence - Special

AIF Images	On
AIF SR Preparation	SR_PERF
Scan	Test
Gadgetron IPR	PROTO2
Temporal Filter	On
Filter Method	Gaussian
Filter Strength	Medium

Sequence - Assistant

Mode	Min flip angle
Min flip angle	45 deg
Allowed delay	100 s

© 2025 Steffen Johansson R et al. JAMA Network Open.

\\USER\Kliniska hjartan\Hjärtprotokollet\COVID 6m\pre_MOLLI_SA NoScout
TA: 0:52 PM: REF Voxel size: 1.4×1.4×8.0 mmPAT: 2 Rel. SNR: 1.00 : wip_tfi

Properties

Prio recon	Off
Load images to viewer	On
Inline movie	Off
Auto store images	On
Load images to stamp segments	Off
Load images to graphic segments	On
Auto open inline display	Off
Auto close inline display	Off
Start measurement without further preparation	Off
Wait for user to start	Off
Start measurements	Single measurement

Routine

Slice group	1
Slices	5
Dist. factor	80 %
Position	Isocenter
Orientation	Transversal
Phase enc. dir.	A >> P
AutoAlign	---
Phase oversampling	0 %
FoV read	360 mm
FoV phase	75.0 %
Slice thickness	8.0 mm
TR	293.56 ms
TE	1.12 ms
Averages	1
Concatenations	5
Filter	Distortion Corr.(2D)
Coil elements	BO1-3;SP2,3

Contrast - Common

TR	293.56 ms
TE	1.12 ms
Magn. preparation	Non-sel. IR T1map
TI	193 ms
Flip angle	35 deg
Fat suppr.	Fat sat.
Wrap-up Magn.	None

Contrast - Dynamic

Averages	1
Averaging mode	Short term
Reconstruction	Magnitude
Measurements	1
Multiple series	Off

Resolution - Common

FoV read	360 mm
FoV phase	75.0 %
Slice thickness	8.0 mm
Base resolution	256
Phase resolution	75 %
Phase partial Fourier	7/8
Trajectory	Cartesian
Interpolation	Off

Resolution - iPAT

PAT mode	GRAPPA

© 2025 Steffen Johansson R et al. JAMA Network Open.

Resolution - iPAT

Accel. factor PE	2
Ref. lines PE	36
Reference scan mode	GRE/separate

Resolution - Filter Image

Image Filter	Off
Distortion Corr.	On
Mode	2D
Unfiltered images	Off
Prescan Normalize	Off
Normalize	Off
B1 filter	Off

Resolution - Filter Rawdata

Raw filter	Off
Elliptical filter	Off
POCS	Off

Geometry - Common

Slice group	1
Slices	5
Dist. factor	80 %
Position	Isocenter
Orientation	Transversal
Phase enc. dir.	A >> P
FoV read	360 mm
FoV phase	75.0 %
Slice thickness	8.0 mm
TR	293.56 ms
Multi-slice mode	Sequential
Series	Interl. in B.-h.
Concatenations	5

Geometry - AutoAlign

Slice group	1
Position	Isocenter
Orientation	Transversal
Phase enc. dir.	A >> P
AutoAlign	---
Initial Position	Isocenter
Phase	0.0 mm
Read	0.0 mm
Shift	0.0 mm
Initial Rotation	0.00 deg
Initial Orientation	Transversal

Geometry - Saturation

Fat suppr.	Fat sat.
Wrap-up Magn.	None
Special sat.	None

Geometry - Navigator

Geometry - Tim Planning Suite

Set-n-Go Protocol	Off
Table position	H
Table position	0 mm
Inline Composing	Off

System - Miscellaneous

Positioning mode	REF
Table position	H
Table position	0 mm
MSMA	S - C - T
Sagittal	R >> L
Coronal	A >> P
Transversal	F >> H
Coil Combine Mode	Adaptive Combine
Save uncombined	Off
Matrix Optimization	Off
Coil Focus	Flat
AutoAlign	---
Coil Select Mode	Default

System - Adjustments

B0 Shim mode	Cardiac
Adjust with body coil	On
Confirm freq. adjustment	Off
Assume Dominant Fat	Off
Assume Silicone	Off
Adjustment Tolerance	Auto

System - Adjust Volume

Position	Isocenter
Orientation	Transversal
Rotation	0.00 deg
A >> P	270 mm
R >> L	360 mm
F >> H	66 mm
Reset	Off

System - Tx/Rx

Frequency 1H	63.672141 MHz
Correction factor	1
Gain	High
Img. Scale Cor.	1.000
Reset	Off
? Ref. amplitude 1H	0.000 V

Physio - Signal1

1st Signal/Mode	ECG/Trigger
Average cycle	430 ± 159 ms
Average cycle	No Signal ms
Captured cycle	430 ± 159 ms
Acquisition window	712 ms
Trigger pulse	1
Trigger delay	418 ms
TR	293.56 ms
Concatenations	5
Segments	72
Phases	1
Adaptive Triggering	Off

Physio - Cardiac

Tagging	None
Magn. preparation	Non-sel. IR T1map
TI	193 ms
Fat suppr.	Fat sat.
Dark blood	Off
FoV read	360 mm
FoV phase	75.0 %
Phase resolution	75 %
Cine	Off
Trajectory	Cartesian

Physio - Cardiac

Dummy heartbeats	0
Motion Correction	Standard

Physio - PACE

Resp. control	Breath-hold
Concatenations	5

Inline - Common

Subtract	Off
Measurements	1
StdDev	Off
Motion Correction	Standard
Save original images	On

Inline - Cardiac

Inline Evaluation	T1 map
Magn. preparation	Non-sel. IR T1map
Num. of preps	2
Sampling duration 1	5 sec.
Sampling duration 2	3 sec.
Contrasts	1
TE	1.12 ms
TR	293.56 ms
Recovery duration 1	3 sec.
Recovery duration 2	0 sec.
Motion Correction	Standard
Save original images	On

Inline - MIP

MIP-Sag	Off
MIP-Cor	Off
MIP-Tra	Off
MIP-Time	Off
Save original images	On

Inline - Composing

Inline Composing	Off
Distortion Corr.	On
Mode	2D
Unfiltered images	Off

Sequence - Part 1

Introduction	Off
Dimension	2D
Reordering	Linear
Asymmetric echo	Weak
Contrasts	1
Optimization	Min. TE TR
Multi-slice mode	Sequential
Echo spacing	2.7 ms
Sequence type	Trufi
Bandwidth	1085 Hz/Px

Sequence - Part 2

Define	Shots
Shots per slice	1
Segments	72
Trufi delta freq.	0 Hz
RF pulse type	Fast
Gradient mode	Fast
Excitation	Slice-sel.
Flip angle mode	Constant
Cine	Off

© 2025 Steffen Johansson R et al. JAMA Network Open.

Sequence - Special

Partition coeff map	Off
ECV map	Off
Synth ECV map	Off
TI scout mode	Off
Error map	On
Synth PSIR	Off
Periods in seconds	On
16 bit images	Off
T1 sampling scheme	Native

Sequence - Assistant

Mode	Off
Allowed delay	0 s

© 2025 Steffen Johansson R et al. JAMA Network Open.

\\USER\Kliniska hjartan\Hjärtprotokollet\COVID 6m\pre_MOLLI_4ch_NoScout
TA: 0:13 PM: REF Voxel size: 1.4×1.4×8.0 mmPAT: 2 Rel. SNR: 1.00 : wip_tfi

Properties

Prio recon	Off
Load images to viewer	On
Inline movie	Off
Auto store images	On
Load images to stamp segments	Off
Load images to graphic segments	On
Auto open inline display	Off
Auto close inline display	Off
Start measurement without further preparation	Off
Wait for user to start	Off
Start measurements	Single measurement

Routine

Slice group	1
Slices	1
Dist. factor	20 %
Position	Isocenter
Orientation	Transversal
Phase enc. dir.	A >> P
AutoAlign	---
Phase oversampling	0 %
FoV read	360 mm
FoV phase	75.0 %
Slice thickness	8.0 mm
TR	293.56 ms
TE	1.12 ms
Averages	1
Concatenations	1
Filter	Distortion Corr.(2D)
Coil elements	BO1-3;SP2,3

Contrast - Common

TR	293.56 ms
TE	1.12 ms
Magn. preparation	Non-sel. IR T1map
TI	193 ms
Flip angle	35 deg
Fat suppr.	Fat sat.
Wrap-up Magn.	None

Contrast - Dynamic

Averages	1
Averaging mode	Short term
Reconstruction	Magnitude
Measurements	1
Multiple series	Off

Resolution - Common

FoV read	360 mm
FoV phase	75.0 %
Slice thickness	8.0 mm
Base resolution	256
Phase resolution	75 %
Phase partial Fourier	7/8
Trajectory	Cartesian
Interpolation	Off

Resolution - iPAT

PAT mode	GRAPPA

Resolution - iPAT

Accel. factor PE	2
Ref. lines PE	36
Reference scan mode	GRE/separate

Resolution - Filter Image

Image Filter	Off
Distortion Corr.	On
Mode	2D
Unfiltered images	Off
Prescan Normalize	Off
Normalize	Off
B1 filter	Off

Resolution - Filter Rawdata

Raw filter	Off
Elliptical filter	Off
POCS	Off

Geometry - Common

Slice group	1
Slices	1
Dist. factor	20 %
Position	Isocenter
Orientation	Transversal
Phase enc. dir.	A >> P
FoV read	360 mm
FoV phase	75.0 %
Slice thickness	8.0 mm
TR	293.56 ms
Multi-slice mode	Sequential
Series	Interleaved
Concatenations	1

Geometry - AutoAlign

Slice group	1
Position	Isocenter
Orientation	Transversal
Phase enc. dir.	A >> P
AutoAlign	---
Initial Position	Isocenter
Phase	0.0 mm
Read	0.0 mm
Shift	0.0 mm
Initial Rotation	0.00 deg
Initial Orientation	Transversal

Geometry - Saturation

Fat suppr.	Fat sat.
Wrap-up Magn.	None
Special sat.	None

Geometry - Navigator

Geometry - Tim Planning Suite

Set-n-Go Protocol	Off
Table position	H
Table position	0 mm
Inline Composing	Off

© 2025 Steffen Johansson R et al. JAMA Network Open.

System - Miscellaneous

Positioning mode	REF
Table position	H
Table position	0 mm
MSMA	S - C - T
Sagittal	R >> L
Coronal	A >> P
Transversal	F >> H
Coil Combine Mode	Adaptive Combine
Save uncombined	Off
Matrix Optimization	Off
Coil Focus	Flat
AutoAlign	---
Coil Select Mode	Default

System - Adjustments

B0 Shim mode	Cardiac
Adjust with body coil	On
Confirm freq. adjustment	Off
Assume Dominant Fat	Off
Assume Silicone	Off
Adjustment Tolerance	Auto

System - Adjust Volume

Position	Isocenter
Orientation	Transversal
Rotation	0.00 deg
A >> P	270 mm
R >> L	360 mm
F >> H	8 mm
Reset	Off

System - Tx/Rx

Frequency 1H	63.672141 MHz
Correction factor	1
Gain	High
Img. Scale Cor.	1.000
Reset	Off
? Ref. amplitude 1H	0.000 V

Physio - Signal1

1st Signal/Mode	ECG/Trigger
Average cycle	430 ± 159 ms
Average cycle	No Signal ms
Captured cycle	430 ± 159 ms
Acquisition window	618 ms
Trigger pulse	1
Trigger delay	324 ms
TR	293.56 ms
Concatenations	1
Segments	72
Phases	1
Adaptive Triggering	Off

Physio - Cardiac

Tagging	None
Magn. preparation	Non-sel. IR T1map
TI	193 ms
Fat suppr.	Fat sat.
Dark blood	Off
FoV read	360 mm
FoV phase	75.0 %
Phase resolution	75 %
Cine	Off
Trajectory	Cartesian

Physio - Cardiac

Dummy heartbeats	0
Motion Correction	Standard

Physio - PACE

Resp. control	Off
Concatenations	1

Inline - Common

Subtract	Off
Measurements	1
StdDev	Off
Motion Correction	Standard
Save original images	On

Inline - Cardiac

Inline Evaluation	T1 map
Magn. preparation	Non-sel. IR T1map
Num. of preps	2
Sampling duration 1	5 sec.
Sampling duration 2	3 sec.
Contrasts	1
TE	1.12 ms
TR	293.56 ms
Recovery duration 1	3 sec.
Recovery duration 2	0 sec.
Motion Correction	Standard
Save original images	On

Inline - MIP

MIP-Sag	Off
MIP-Cor	Off
MIP-Tra	Off
MIP-Time	Off
Save original images	On

Inline - Composing

Inline Composing	Off
Distortion Corr.	On
Mode	2D
Unfiltered images	Off

Sequence - Part 1

Introduction	Off
Dimension	2D
Reordering	Linear
Asymmetric echo	Weak
Contrasts	1
Optimization	Min. TE TR
Multi-slice mode	Sequential
Echo spacing	2.7 ms
Sequence type	Trufi
Bandwidth	1085 Hz/Px

Sequence - Part 2

Define	Shots
Shots per slice	1
Segments	72
Trufi delta freq.	0 Hz
RF pulse type	Fast
Gradient mode	Fast
Excitation	Slice-sel.
Flip angle mode	Constant
Cine	Off

© 2025 Steffen Johansson R et al. JAMA Network Open.

Sequence - Special

Partition coeff map	Off
ECV map	Off
Synth ECV map	Off
TI scout mode	Off
Error map	On
Synth PSIR	Off
Periods in seconds	On
16 bit images	Off
T1 sampling scheme	Native

Sequence - Assistant

Mode	Off
Allowed delay	0 s

© 2025 Steffen Johansson R et al. JAMA Network Open.

\\USER\Kliniska hjartan\Hjärtprotokollet\COVID 6m\T2map_SA
TA: 0:47 PM: FIX Voxel size: 1.4×1.4×8.0 mmPAT: 2 Rel. SNR: 1.00 : wip_tfi

Properties

Prio recon	Off
Load images to viewer	On
Inline movie	Off
Auto store images	On
Load images to stamp segments	Off
Load images to graphic segments	On
Auto open inline display	Off
Auto close inline display	Off
Start measurement without further preparation	Off
Wait for user to start	Off
Start measurements	Single measurement

Routine

Slice group	1
Slices	5
Dist. factor	80 %
Position	Isocenter
Orientation	Transversal
Phase enc. dir.	A >> P
AutoAlign	---
Phase oversampling	0 %
FoV read	360 mm
FoV phase	75.0 %
Slice thickness	8.0 mm
TR	260.68 ms
TE	1.19 ms
Averages	1
Concatenations	5
Filter	Distortion Corr.(2D)
Coil elements	BO1-3;SP2,3

Contrast - Common

TR	260.68 ms
TE	1.19 ms
Magn. preparation	T2 prep. adiab.
T2 prep. duration 1	0 ms
T2 prep. duration 2	25 ms
T2 prep. duration 3	55 ms
Flip angle	70 deg
Fat suppr.	None
Wrap-up Magn.	None

Contrast - Dynamic

Averages	1
Averaging mode	Short term
Reconstruction	Magnitude
Measurements	1
Multiple series	Off

Resolution - Common

FoV read	360 mm
FoV phase	75.0 %
Slice thickness	8.0 mm
Base resolution	256
Phase resolution	75 %
Phase partial Fourier	7/8
Trajectory	Cartesian
Interpolation	Off

Resolution - iPAT

PAT mode	GRAPPA
Accel. factor PE	2
Ref. lines PE	36
Reference scan mode	GRE/separate

Resolution - Filter Image

Image Filter	Off
Distortion Corr.	On
Mode	2D
Unfiltered images	Off
Prescan Normalize	Off
Normalize	Off
B1 filter	Off

Resolution - Filter Rawdata

Raw filter	Off
Elliptical filter	Off
POCS	Off

Geometry - Common

Slice group	1
Slices	5
Dist. factor	80 %
Position	Isocenter
Orientation	Transversal
Phase enc. dir.	A >> P
FoV read	360 mm
FoV phase	75.0 %
Slice thickness	8.0 mm
TR	260.68 ms
Multi-slice mode	Sequential
Series	Interl. in B.-h.
Concatenations	5

Geometry - AutoAlign

Slice group	1
Position	Isocenter
Orientation	Transversal
Phase enc. dir.	A >> P
AutoAlign	---
Initial Position	Isocenter
Phase	0.0 mm
Read	0.0 mm
Shift	0.0 mm
Initial Rotation	0.00 deg
Initial Orientation	Transversal

Geometry - Saturation

Fat suppr.	None
Wrap-up Magn.	None
Special sat.	None

Geometry - Navigator

Geometry - Tim Planning Suite

Set-n-Go Protocol	Off
Table position	H
Table position	0 mm
Inline Composing	Off

© 2025 Steffen Johansson R et al. JAMA Network Open.

System - Miscellaneous

Positioning mode	FIX
Table position	H
Table position	0 mm
MSMA	S - C - T
Sagittal	R >> L
Coronal	A >> P
Transversal	F >> H
Coil Combine Mode	Adaptive Combine
Save uncombined	Off
Matrix Optimization	Off
Coil Focus	Flat
AutoAlign	---
Coil Select Mode	Default

System - Adjustments

B0 Shim mode	Cardiac
Adjust with body coil	On
Confirm freq. adjustment	Off
Assume Dominant Fat	Off
Assume Silicone	Off
Adjustment Tolerance	Auto

System - Adjust Volume

Position	Isocenter
Orientation	Transversal
Rotation	0.00 deg
A >> P	270 mm
R >> L	360 mm
F >> H	66 mm
Reset	Off

System - Tx/Rx

Frequency 1H	63.672141 MHz
Correction factor	1
Gain	High
Img. Scale Cor.	1.000
Reset	Off
? Ref. amplitude 1H	0.000 V

Physio - Signal1

1st Signal/Mode	ECG/Trigger
Average cycle	430 ± 159 ms
Average cycle	No Signal ms
Captured cycle	430 ± 159 ms
Acquisition window	744 ms
Trigger pulse	1
Trigger delay	483 ms
TR	260.68 ms
Concatenations	5
Segments	72
Phases	1
Adaptive Triggering	Off

Physio - Cardiac

Tagging	None
Magn. preparation	T2 prep. adiab.
T2 prep. duration 1	0 ms
T2 prep. duration 2	25 ms
T2 prep. duration 3	55 ms
Fat suppr.	None
Dark blood	Off
FoV read	360 mm
FoV phase	75.0 %
Phase resolution	75 %

Physio - Cardiac

Cine	Off
Trajectory	Cartesian
Dummy heartbeats	0
Motion Correction	Standard

Physio - PACE

Resp. control	Breath-hold
Concatenations	5

Inline - Common

Subtract	Off
Measurements	1
StdDev	Off
Motion Correction	Standard
Save original images	On

Inline - Cardiac

Inline Evaluation	T2 map
Magn. preparation	T2 prep. adiab.
Num. of preps	3
T2 prep. duration 1	0 ms
T2 prep. duration 2	25 ms
T2 prep. duration 3	55 ms
Contrasts	1
TE	1.19 ms
TR	260.68 ms
Recovery duration	3 sec.
Motion Correction	Standard
Save original images	On

Inline - MIP

MIP-Sag	Off
MIP-Cor	Off
MIP-Tra	Off
MIP-Time	Off
Save original images	On

Inline - Composing

Inline Composing	Off
Distortion Corr.	On
Mode	2D
Unfiltered images	Off

Sequence - Part 1

Introduction	Off
Dimension	2D
Reordering	Linear
Asymmetric echo	Weak
Contrasts	1
Optimization	Min. TE TR
Multi-slice mode	Sequential
Echo spacing	2.8 ms
Sequence type	Trufi
Bandwidth	1149 Hz/Px

Sequence - Part 2

Define	Shots
Shots per slice	1
Segments	72
Trufi delta freq.	0 Hz
RF pulse type	Fast
Gradient mode	Fast
Excitation	Slice-sel.

© 2025 Steffen Johansson R et al. JAMA Network Open.

Sequence - Part 2

Flip angle mode	Constant
Cine	Off

Sequence - Special

Periods in seconds	On
16 bit images	Off

Sequence - Assistant

Mode	Off
Allowed delay	0 s

© 2025 Steffen Johansson R et al. JAMA Network Open.

\\USER\Kliniska hjartan\Hjärtprotokollet\COVID 6m\pre_MOLLI_SA mid_NoScout
TA: 0:10 PM: FIX Voxel size: 1.4×1.4×8.0 mmPAT: 2 Rel. SNR: 1.00 : wip_tfi

Properties

Prio recon	Off
Load images to viewer	On
Inline movie	Off
Auto store images	On
Load images to stamp segments	Off Load
images to graphic segments	On Auto
open inline display	Off
Auto close inline display	Off
Start measurement without further preparation	Off
Wait for user to start	Off
Start measurements	Single measurement

Routine

Slice group	1
Slices	1
Dist. factor	200 %
Position	Isocenter
Orientation	Transversal
Phase enc. dir.	A >> P
AutoAlign	---
Phase oversampling	0 %
FoV read	360 mm
FoV phase	75.0 %
Slice thickness	8.0 mm
TR	293.56 ms
TE	1.12 ms
Averages	1
Concatenations	1
Filter	Distortion Corr.(2D)
Coil elements	BO1-3;SP2,3

Contrast - Common

TR	293.56 ms
TE	1.12 ms
Magn. preparation	Non-sel. IR T1map
TI	193 ms
Flip angle	35 deg
Fat suppr.	Fat sat.
Wrap-up Magn.	None

Contrast - Dynamic

Averages	1
Averaging mode	Short term
Reconstruction	Magnitude
Measurements	1
Multiple series	Off

Resolution - Common

FoV read	360 mm
FoV phase	75.0 %
Slice thickness	8.0 mm
Base resolution	256
Phase resolution	75 %
Phase partial Fourier	7/8
Trajectory	Cartesian
Interpolation	Off

Resolution - iPAT

PAT mode	GRAPPA

Resolution - iPAT

Accel. factor PE	2
Ref. lines PE	36
Reference scan mode	GRE/separate

Resolution - Filter Image

Image Filter	Off
Distortion Corr.	On
Mode	2D
Unfiltered images	Off
Prescan Normalize	Off
Normalize	Off
B1 filter	Off

Resolution - Filter Rawdata

Raw filter	Off
Elliptical filter	Off
POCS	Off

Geometry - Common

Slice group	1
Slices	1
Dist. factor	200 %
Position	Isocenter
Orientation	Transversal
Phase enc. dir.	A >> P
FoV read	360 mm
FoV phase	75.0 %
Slice thickness	8.0 mm
TR	293.56 ms
Multi-slice mode	Sequential
Series	Interl. in B.-h.
Concatenations	1

Geometry - AutoAlign

Slice group	1
Position	Isocenter
Orientation	Transversal
Phase enc. dir.	A >> P
AutoAlign	---
Initial Position	Isocenter
Phase	0.0 mm
Read	0.0 mm
Shift	0.0 mm
Initial Rotation	0.00 deg
Initial Orientation	Transversal

Geometry - Saturation

Fat suppr.	Fat sat.
Wrap-up Magn.	None
Special sat.	None

Geometry - Navigator

Geometry - Tim Planning Suite

Set-n-Go Protocol	Off
Table position	H
Table position	0 mm
Inline Composing	Off

© 2025 Steffen Johansson R et al. JAMA Network Open.

System - Miscellaneous

Positioning mode	FIX
Table position	H
Table position	0 mm
MSMA	S - C - T
Sagittal	R >> L
Coronal	A >> P
Transversal	F >> H
Coil Combine Mode	Adaptive Combine
Save uncombined	Off
Matrix Optimization	Off
Coil Focus	Flat
AutoAlign	---
Coil Select Mode	Default

System - Adjustments

B0 Shim mode	Cardiac
Adjust with body coil	On
Confirm freq. adjustment	Off
Assume Dominant Fat	Off
Assume Silicone	Off
Adjustment Tolerance	Auto

System - Adjust Volume

Position	Isocenter
Orientation	Transversal
Rotation	0.00 deg
A >> P	270 mm
R >> L	360 mm
F >> H	8 mm
Reset	Off

System - Tx/Rx

Frequency 1H	63.672141 MHz
Correction factor	1
Gain	High
Img. Scale Cor.	1.000
Reset	Off
? Ref. amplitude 1H	0.000 V

Physio - Signal1

1st Signal/Mode	ECG/Trigger
Average cycle	430 ± 159 ms
Average cycle	No Signal ms
Captured cycle	430 ± 159 ms
Acquisition window	712 ms
Trigger pulse	1
Trigger delay	418 ms
TR	293.56 ms
Concatenations	1
Segments	72
Phases	1
Adaptive Triggering	Off

Physio - Cardiac

Tagging	None
Magn. preparation	Non-sel. IR T1map
TI	193 ms
Fat suppr.	Fat sat.
Dark blood	Off
FoV read	360 mm
FoV phase	75.0 %
Phase resolution	75 %
Cine	Off
Trajectory	Cartesian

Physio - Cardiac

Dummy heartbeats	0
Motion Correction	Standard

Physio - PACE

Resp. control	Breath-hold
Concatenations	1

Inline - Common

Subtract	Off
Measurements	1
StdDev	Off
Motion Correction	Standard
Save original images	On

Inline - Cardiac

Inline Evaluation	T1 map
Magn. preparation	Non-sel. IR T1map
Num. of preps	2
Sampling duration 1	5 sec.
Sampling duration 2	3 sec.
Contrasts	1
TE	1.12 ms
TR	293.56 ms
Recovery duration 1	3 sec.
Recovery duration 2	0 sec.
Motion Correction	Standard
Save original images	On

Inline - MIP

MIP-Sag	Off
MIP-Cor	Off
MIP-Tra	Off
MIP-Time	Off
Save original images	On

Inline - Composing

Inline Composing	Off
Distortion Corr.	On
Mode	2D
Unfiltered images	Off

Sequence - Part 1

Introduction	Off
Dimension	2D
Reordering	Linear
Asymmetric echo	Weak
Contrasts	1
Optimization	Min. TE TR
Multi-slice mode	Sequential
Echo spacing	2.7 ms
Sequence type	Trufi
Bandwidth	1085 Hz/Px

Sequence - Part 2

Define	Shots
Shots per slice	1
Segments	72
Trufi delta freq.	0 Hz
RF pulse type	Fast
Gradient mode	Fast
Excitation	Slice-sel.
Flip angle mode	Constant
Cine	Off

© 2025 Steffen Johansson R et al. JAMA Network Open.

Sequence - Special

Partition coeff map	Off
ECV map	Off
Synth ECV map	Off
TI scout mode	Off
Error map	On
Synth PSIR	Off
Periods in seconds	On
16 bit images	Off
T1 sampling scheme	Native

Sequence - Assistant

Mode	Off
Allowed delay	0 s

© 2025 Steffen Johansson R et al. JAMA Network Open.

\\USER\Kliniska hjartan\Hjärtprotokollet\COVID 6m\T2map_SA mid
TA: 9.5 s PM: FIX Voxel size: 1.4×1.4×8.0 mmPAT: 2 Rel. SNR: 1.00 : wip_tfi

Properties

Prio recon	Off
Load images to viewer	On
Inline movie	Off
Auto store images	On
Load images to stamp segments	Off Load
images to graphic segments	On Auto
open inline display	Off
Auto close inline display	Off
Start measurement without further preparation	Off
Wait for user to start	Off
Start measurements	Single measurement

Routine

Slice group	1
Slices	1
Dist. factor	200 %
Position	Isocenter
Orientation	Transversal
Phase enc. dir.	A >> P
AutoAlign	---
Phase oversampling	0 %
FoV read	360 mm
FoV phase	75.0 %
Slice thickness	8.0 mm
TR	260.68 ms
TE	1.19 ms
Averages	1
Concatenations	1
Filter	Distortion Corr.(2D)
Coil elements	BO1-3;SP2,3

Contrast - Common

TR	260.68 ms
TE	1.19 ms
Magn. preparation	T2 prep. adiab.
T2 prep. duration 1	0 ms
T2 prep. duration 2	25 ms
T2 prep. duration 3	55 ms
Flip angle	70 deg
Fat suppr.	None
Wrap-up Magn.	None

Contrast - Dynamic

Averages	1
Averaging mode	Short term
Reconstruction	Magnitude
Measurements	1
Multiple series	Off

Resolution - Common

FoV read	360 mm
FoV phase	75.0 %
Slice thickness	8.0 mm
Base resolution	256
Phase resolution	75 %
Phase partial Fourier	7/8
Trajectory	Cartesian
Interpolation	Off

Resolution - iPAT

PAT mode	GRAPPA
Accel. factor PE	2
Ref. lines PE	36
Reference scan mode	GRE/separate

Resolution - Filter Image

Image Filter	Off
Distortion Corr.	On
Mode	2D
Unfiltered images	Off
Prescan Normalize	Off
Normalize	Off
B1 filter	Off

Resolution - Filter Rawdata

Raw filter	Off
Elliptical filter	Off
POCS	Off

Geometry - Common

Slice group	1
Slices	1
Dist. factor	200 %
Position	Isocenter
Orientation	Transversal
Phase enc. dir.	A >> P
FoV read	360 mm
FoV phase	75.0 %
Slice thickness	8.0 mm
TR	260.68 ms
Multi-slice mode	Sequential
Series	Interl. in B.-h.
Concatenations	1

Geometry - AutoAlign

Slice group	1
Position	Isocenter
Orientation	Transversal
Phase enc. dir.	A >> P
AutoAlign	---
Initial Position	Isocenter
Phase	0.0 mm
Read	0.0 mm
Shift	0.0 mm
Initial Rotation	0.00 deg
Initial Orientation	Transversal

Geometry - Saturation

Fat suppr.	None
Wrap-up Magn.	None
Special sat.	None

Geometry - Navigator

Geometry - Tim Planning Suite

Set-n-Go Protocol	Off
Table position	H
Table position	0 mm
Inline Composing	Off

© 2025 Steffen Johansson R et al. JAMA Network Open.

System - Miscellaneous

Positioning mode	FIX
Table position	H
Table position	0 mm
MSMA	S - C - T
Sagittal	R >> L
Coronal	A >> P
Transversal	F >> H
Coil Combine Mode	Adaptive Combine
Save uncombined	Off
Matrix Optimization	Off
Coil Focus	Flat
AutoAlign	---
Coil Select Mode	Default

System - Adjustments

B0 Shim mode	Cardiac
Adjust with body coil	On
Confirm freq. adjustment	Off
Assume Dominant Fat	Off
Assume Silicone	Off
Adjustment Tolerance	Auto

System - Adjust Volume

Position	Isocenter
Orientation	Transversal
Rotation	0.00 deg
A >> P	270 mm
R >> L	360 mm
F >> H	8 mm
Reset	Off

System - Tx/Rx

Frequency 1H	63.672141 MHz
Correction factor	1
Gain	High
Img. Scale Cor.	1.000
Reset	Off
? Ref. amplitude 1H	0.000 V

Physio - Signal1

1st Signal/Mode	ECG/Trigger
Average cycle	430 ± 159 ms
Average cycle	No Signal ms
Captured cycle	430 ± 159 ms
Acquisition window	744 ms
Trigger pulse	1
Trigger delay	483 ms
TR	260.68 ms
Concatenations	1
Segments	72
Phases	1
Adaptive Triggering	Off

Physio - Cardiac

Tagging	None
Magn. preparation	T2 prep. adiab.
T2 prep. duration 1	0 ms
T2 prep. duration 2	25 ms
T2 prep. duration 3	55 ms
Fat suppr.	None
Dark blood	Off
FoV read	360 mm
FoV phase	75.0 %
Phase resolution	75 %

Physio - Cardiac

Cine	Off
Trajectory	Cartesian
Dummy heartbeats	0
Motion Correction	Standard

Physio - PACE

Resp. control	Breath-hold
Concatenations	1

Inline - Common

Subtract	Off
Measurements	1
StdDev	Off
Motion Correction	Standard
Save original images	On

Inline - Cardiac

Inline Evaluation	T2 map
Magn. preparation	T2 prep. adiab.
Num. of preps	3
T2 prep. duration 1	0 ms
T2 prep. duration 2	25 ms
T2 prep. duration 3	55 ms
Contrasts	1
TE	1.19 ms
TR	260.68 ms
Recovery duration	3 sec.
Motion Correction	Standard
Save original images	On

Inline - MIP

MIP-Sag	Off
MIP-Cor	Off
MIP-Tra	Off
MIP-Time	Off
Save original images	On

Inline - Composing

Inline Composing	Off
Distortion Corr.	On
Mode	2D
Unfiltered images	Off

Sequence - Part 1

Introduction	Off
Dimension	2D
Reordering	Linear
Asymmetric echo	Weak
Contrasts	1
Optimization	Min. TE TR
Multi-slice mode	Sequential
Echo spacing	2.8 ms
Sequence type	Trufi
Bandwidth	1149 Hz/Px

Sequence - Part 2

Define	Shots
Shots per slice	1
Segments	72
Trufi delta freq.	0 Hz
RF pulse type	Fast
Gradient mode	Fast
Excitation	Slice-sel.

© 2025 Steffen Johansson R et al. JAMA Network Open.

Sequence - Part 2

Flip angle mode	Constant
Cine	Off

Sequence - Special

Periods in seconds	On
16 bit images	Off

Sequence - Assistant

Mode	Off
Allowed delay	0 s

© 2025 Steffen Johansson R et al. JAMA Network Open.

\\USER\Kliniska hjartan\Hjärtprotokollet\COVID 6m\SSFP_Perf_MBF_AI STRESS
TA: 0:52 PM: FIX Voxel size: 1.9×1.9×8.0 mmPAT: 3 Rel. SNR: 1.00 : tfi

Properties

Prio recon	Off
Load images to viewer	Off
Inline movie	Off
Auto store images	On
Load images to stamp segments	Off Load
images to graphic segments	On Auto
open inline display	Off
Auto close inline display	Off
Start measurement without further preparation	Off
Wait for user to start	Off
Start measurements	Single measurement

Routine

Slice group	1
Slices	3
Dist. factor	100 %
Position	Isocenter
Orientation	Transversal
Phase enc. dir.	A >> P
AutoAlign	---
Phase oversampling	0 %
FoV read	360 mm
FoV phase	75.0 %
Slice thickness	8.0 mm
TR	142.00 ms
TE	1.04 ms
Averages	1
Concatenations	1
Filter	Distortion Corr.(2D)
Coil elements	BO1-3;SP3,4

Contrast - Common

TR	142.00 ms
TE	1.04 ms
Magn. preparation	Non-sel. SR perf
TI	105 ms
Flip angle	50 deg
Fat suppr.	Fat sat.
Wrap-up Magn.	None

Contrast - Dynamic

Averages	1
Averaging mode	Long term
Reconstruction	Magnitude
Measurements	60
Pause after meas. 1	0.0 s
Pause after meas. 2	0.0 s
Pause after meas. 3	0.0 s
Pause after meas. 4	0.0 s
Pause after meas. 5	0.0 s
Pause after meas. 6	0.0 s
Pause after meas. 7	0.0 s
Pause after meas. 8	0.0 s
Pause after meas. 9	0.0 s
Pause after meas. 10	0.0 s
Pause after meas. 11	0.0 s
Pause after meas. 12	0.0 s
Pause after meas. 13	0.0 s
Pause after meas. 14	0.0 s

Contrast - Dynamic

Pause after meas. 15	0.0 s
Pause after meas. 16	0.0 s
Pause after meas. 17	0.0 s
Pause after meas. 18	0.0 s
Pause after meas. 19	0.0 s
Pause after meas. 20	0.0 s
Pause after meas. 21	0.0 s
Pause after meas. 22	0.0 s
Pause after meas. 23	0.0 s
Pause after meas. 24	0.0 s
Pause after meas. 25	0.0 s
Pause after meas. 26	0.0 s
Pause after meas. 27	0.0 s
Pause after meas. 28	0.0 s
Pause after meas. 29	0.0 s
Pause after meas. 30	0.0 s
Pause after meas. 31	0.0 s
Pause after meas. 32	0.0 s
Pause after meas. 33	0.0 s
Pause after meas. 34	0.0 s
Pause after meas. 35	0.0 s
Pause after meas. 36	0.0 s
Pause after meas. 37	0.0 s
Pause after meas. 38	0.0 s
Pause after meas. 39	0.0 s
Pause after meas. 40	0.0 s
Pause after meas. 41	0.0 s
Pause after meas. 42	0.0 s
Pause after meas. 43	0.0 s
Pause after meas. 44	0.0 s
Pause after meas. 45	0.0 s
Pause after meas. 46	0.0 s
Pause after meas. 47	0.0 s
Pause after meas. 48	0.0 s
Pause after meas. 49	0.0 s
Pause after meas. 50	0.0 s
Pause after meas. 51	0.0 s
Pause after meas. 52	0.0 s
Pause after meas. 53	0.0 s
Pause after meas. 54	0.0 s
Pause after meas. 55	0.0 s
Pause after meas. 56	0.0 s
Pause after meas. 57	0.0 s
Pause after meas. 58	0.0 s
Pause after meas. 59	0.0 s
Proton Dens. Maps	3
Multiple series	Off

Resolution - Common

FoV read	360 mm
FoV phase	75.0 %
Slice thickness	8.0 mm
Base resolution	192
Phase resolution	77 %
Phase partial Fourier	6/8
Trajectory	Cartesian
Interpolation	Off

Resolution - iPAT

PAT mode	GRAPPA

© 2025 Steffen Johansson R et al. JAMA Network Open.

Resolution - iPAT

Accel. factor PE	3
Reference scan mode	T-PAT

Resolution - Filter Image

Image Filter	Off
Distortion Corr.	On
Mode	2D
Unfiltered images	Off
Prescan Normalize	Off
Normalize	Off
B1 filter	Off

Resolution - Filter Rawdata

Raw filter	Off
Elliptical filter	Off
POCS	Off

Geometry - Common

Slice group	1
Slices	3
Dist. factor	100 %
Position	Isocenter
Orientation	Transversal
Phase enc. dir.	A >> P
FoV read	360 mm
FoV phase	75.0 %
Slice thickness	8.0 mm
TR	142.00 ms
Multi-slice mode	Single shot
Series	Ascending
Concatenations	1

Geometry - AutoAlign

Slice group	1
Position	Isocenter
Orientation	Transversal
Phase enc. dir.	A >> P
AutoAlign	---
Initial Position	Isocenter
Phase	0.0 mm
Read	0.0 mm
Shift	0.0 mm
Initial Rotation	0.00 deg
Initial Orientation	Transversal

Geometry - Saturation

Fat suppr.	Fat sat.
Wrap-up Magn.	None
Special sat.	None

Geometry - Navigator

Geometry - Tim Planning Suite

Set-n-Go Protocol	Off
Table position	H
Table position	0 mm
Inline Composing	Off

System - Miscellaneous

Positioning mode	FIX
Table position	H
Table position	0 mm
MSMA	S - C - T

System - Miscellaneous

Sagittal	R >> L
Coronal	A >> P
Transversal	F >> H
Coil Combine Mode	Sum of Squares
Save uncombined	Off
Matrix Optimization	Off
Coil Focus	Flat
AutoAlign	---
Coil Select Mode	Default

System - Adjustments

B0 Shim mode	Cardiac
Adjust with body coil	On
Confirm freq. adjustment	Off
Assume Dominant Fat	Off
Assume Silicone	Off
Adjustment Tolerance	Auto

System - Adjust Volume

Position	Isocenter
Orientation	Transversal
Rotation	0.00 deg
A >> P	270 mm
R >> L	360 mm
F >> H	40 mm
Reset	Off

System - Tx/Rx

Frequency 1H	63.672141 MHz
Correction factor	1
Gain	High
Img. Scale Cor.	1.000
Reset	Off
? Ref. amplitude 1H	0.000 V

Physio - Signal1

1st Signal/Mode	ECG/Trigger
Average cycle	430 ± 159 ms
Average cycle	No Signal ms
Captured cycle	430 ± 159 ms
Acquisition window	491 ms
Trigger pulse	1
Trigger delay	0 ms
TR	142.00 ms
Concatenations	1
Segments	37
Phases	1
Adaptive Triggering	Off

Physio - Cardiac

Tagging	None
Magn. preparation	Non-sel. SR perf
TI	105 ms
Fat suppr.	Fat sat.
Dark blood	Off
FoV read	360 mm
FoV phase	75.0 %
Phase resolution	77 %
Cine	Off
Trajectory	Cartesian
Dummy heartbeats	0
Motion Correction	None

© 2025 Steffen Johansson R et al. JAMA Network Open.

Physio - PACE

Resp. control	Off
Concatenations	1

Inline - Common

Subtract	Off
Measurements	60
StdDev	Off
Motion Correction	None
Save original images	On

Inline - Cardiac

Inline Evaluation	Off
Magn. preparation	Non-sel. SR perf
TE	1.04 ms
TR	142.00 ms
Motion Correction	None
Save original images	On

Inline - MIP

MIP-Sag	Off
MIP-Cor	Off
MIP-Tra	Off
MIP-Time	Off
Save original images	On

Inline - Composing

Inline Composing	Off
Distortion Corr.	On
Mode	2D
Unfiltered images	Off

Sequence - Part 1

Introduction	Off
Dimension	2D
Reordering	Linear
Asymmetric echo	Allowed
Optimization	Min. TE
Multi-slice mode	Single shot
Echo spacing	2.5 ms
Sequence type	Trufi
Bandwidth	1085 Hz/Px

Sequence - Part 2

Define	Shots
Shots per slice	1
EPI factor	1
Segments	37
Trufi delta freq.	0 Hz
RF pulse type	Fast
Gradient mode	Fast
Excitation	Slice-sel.
Flip angle mode	Constant
Cine	Off

Sequence - Special

AIF Images	On
AIF SR Preparation	SR_PERF
Scan	Test
Gadgetron IPR	PROTO2
Temporal Filter	On
Filter Method	Gaussian
Filter Strength	Medium

Sequence - Assistant

Mode	Min flip angle
Min flip angle	45 deg
Allowed delay	100 s

© 2025 Steffen Johansson R et al. JAMA Network Open.

\\USER\Kliniska hjartan\Hjärtprotokollet\COVID 6m\flow_Q aorta venc 150 NBH

TA: 0:46 PM: ISO Voxel size: 1.3×1.3×5.0 mmPAT: 2 Rel. SNR: 1.00 : fl_r

Properties

Prio recon	Off
Load images to viewer	On
Inline movie	On
Auto store images	On
Load images to stamp segments	On Load
images to graphic segments	On Auto
open inline display	Off
Auto close inline display	Off
Start measurement without further preparation	Off
Wait for user to start	Off
Start measurements	Single measurement

Routine

Slice group	1
Slices	1
Dist. factor	20 %
Position	L5.6 P35.5 H85.1 mm
Orientation	T > S0.1
Phase enc. dir.	A >> P
AutoAlign	---
Phase oversampling	0 %
FoV read	340 mm
FoV phase	68.8 %
Slice thickness	5.0 mm
TR	30.54 ms
TE	2.81 ms
Averages	3
Concatenations	1
Filter	Distortion Corr.(2D)
Coil elements	BO1,2;SP1

Contrast - Common

TR	30.54 ms
TE	2.81 ms
TD	0 ms
Flip angle	20 deg
Wrap-up Magn.	None

Contrast - Dynamic

Averages	3
Averaging mode	Long term
Reconstruction	Magnitude
Measurements	1
Multiple series	Off

Resolution - Common

FoV read	340 mm
FoV phase	68.8 %
Slice thickness	5.0 mm
Base resolution	256
Phase resolution	100 %
Phase partial Fourier	Off
Trajectory	Cartesian
View sharing	Off
Interpolation	Off

Resolution - iPAT

PAT mode	GRAPPA
Accel. factor PE	2

Resolution - iPAT

Ref. lines PE	32
Reference scan mode	Integrated

Resolution - Filter Image

Image Filter	Off
Distortion Corr.	On
Mode	2D
Unfiltered images	Off
Prescan Normalize	Off
Normalize	Off
B1 filter	Off

Resolution - Filter Rawdata

Raw filter	Off
Elliptical filter	Off
POCS	Off

Geometry - Common

Slice group	1
Slices	1
Dist. factor	20 %
Position	L5.6 P35.5 H85.1 mm
Orientation	T > S0.1
Phase enc. dir.	A >> P
FoV read	340 mm
FoV phase	68.8 %
Slice thickness	5.0 mm
TR	30.54 ms
Multi-slice mode	Sequential
Series	Ascending
Concatenations	1

Geometry - AutoAlign

Slice group	1
Position	L5.6 P35.5 H85.1 mm
Orientation	T > S0.1
Phase enc. dir.	A >> P
AutoAlign	---
Initial Position	L5.6 P35.5 H85.1
Phase	35.5 mm
Read	-5.8 mm
Shift	85.0 mm
Initial Rotation	0.00 deg
Initial Orientation	T > S
T > S	0.1
> C	0.0

Geometry - Saturation

Wrap-up Magn.	None
Special sat.	None

Geometry - Navigator

Geometry - Tim Planning Suite

Set-n-Go Protocol	Off
Table position	H
Table position	85 mm
Inline Composing	Off

© 2025 Steffen Johansson R et al. JAMA Network Open.

System - Miscellaneous

Positioning mode	ISO
Table position	H
Table position	85 mm
MSMA	S - C - T
Sagittal	R >> L
Coronal	A >> P
Transversal	F >> H
Coil Combine Mode	Sum of Squares
Matrix Optimization	Off
Coil Focus	Flat
AutoAlign	---
Coil Select Mode	Default

System - Adjustments

B0 Shim mode	Tune up
Adjust with body coil	Off
Confirm freq. adjustment	Off
Assume Dominant Fat	Off
Assume Silicone	Off
Adjustment Tolerance	Auto

System - Adjust Volume

Position	Isocenter
Orientation	Transversal
Rotation	0.00 deg
A >> P	263 mm
R >> L	350 mm
F >> H	350 mm
Reset	Off

System - Tx/Rx

Frequency 1H	63.672141 MHz
Correction factor	1
Gain	High
Img. Scale Cor.	1.000
Reset	Off
? Ref. amplitude 1H	0.000 V

Physio - Signal1

1st Signal/Mode	ECG/Retro
Average cycle	430 ± 159 ms
Average cycle	No Signal ms
Calculated phases	35
TR	30.54 ms
Concatenations	1
Segments	3
Arrhythmia detection	None

Physio - PACE

Resp. control	Off
Concatenations	1

Angio - Common

Flow mode	Single dir.
Encodings	1
Velocity enc.	150 cm/s
Direction	Through plane
Rephased images	On
Magnitude images	On
Magnitude sum	Off
Phase images	On

Angio - Inline

Subtract	Off
Measurements	1
StdDev	Off
Save original images	On

Angio - Cardiac

Inline Evaluation	Off
TE	2.81 ms
TR	30.54 ms
Save original images	On

Angio - MIP

MIP-Sag	Off
MIP-Cor	Off
MIP-Tra	Off
MIP-Time	Off
Save original images	On

Angio - Composing

Inline Composing	Off
Distortion Corr.	On
Mode	2D
Unfiltered images	Off

Sequence - Part 1

Introduction	Off
Dimension	2D
Reordering	Linear
Asymmetric echo	Strong
Flow comp.	Yes
Optimization	Min. TE TR
Multi-slice mode	Sequential
Echo spacing	5.1 ms
Sequence type	Gre
Bandwidth	454 Hz/Px

Sequence - Part 2

Define	Segments
Segments	3
RF pulse type	Fast
Gradient mode	Normal
Excitation	Slice-sel.
Flip angle mode	Constant
RF spoiling	On
Phase Enc. Rewinder	On
Cine	On

Sequence - Assistant

Mode	Off
Allowed delay	0 s

© 2025 Steffen Johansson R et al. JAMA Network Open.

\\USER\Kliniska hjartan\Hjärtprotokollet\COVID 6m\flow_Q pulm venc 100 NBH
TA: 0:49 PM: ISO Voxel size: 1.3×1.3×5.0 mmPAT: 2 Rel. SNR: 1.00 : fl_r

Properties

Prio recon	Off
Load images to viewer	On
Inline movie	On
Auto store images	On
Load images to stamp segments	On Load
images to graphic segments	On Auto
open inline display	Off
Auto close inline display	Off
Start measurement without further preparation	Off
Wait for user to start	Off
Start measurements	Single measurement

Routine

Slice group	1
Slices	1
Dist. factor	20 %
Position	L5.6 P35.5 H85.1 mm
Orientation	T > S0.1
Phase enc. dir.	A >> P
AutoAlign	---
Phase oversampling	0 %
FoV read	340 mm
FoV phase	75.0 %
Slice thickness	5.0 mm
TR	30.78 ms
TE	2.83 ms
Averages	3
Concatenations	1
Filter	Distortion Corr.(2D)
Coil elements	BO1,2;SP1

Contrast - Common

TR	30.78 ms
TE	2.83 ms
TD	0 ms
Flip angle	20 deg
Wrap-up Magn.	None

Contrast - Dynamic

Averages	3
Averaging mode	Long term
Reconstruction	Magnitude
Measurements	1
Multiple series	Off

Resolution - Common

FoV read	340 mm
FoV phase	75.0 %
Slice thickness	5.0 mm
Base resolution	256
Phase resolution	100 %
Phase partial Fourier	Off
Trajectory	Cartesian
View sharing	Off
Interpolation	Off

Resolution - iPAT

PAT mode	GRAPPA
Accel. factor PE	2

Resolution - iPAT

Ref. lines PE	32
Reference scan mode	Integrated

Resolution - Filter Image

Image Filter	Off
Distortion Corr.	On
Mode	2D
Unfiltered images	Off
Prescan Normalize	Off
Normalize	Off
B1 filter	Off

Resolution - Filter Rawdata

Raw filter	Off
Elliptical filter	Off
POCS	Off

Geometry - Common

Slice group	1
Slices	1
Dist. factor	20 %
Position	L5.6 P35.5 H85.1 mm
Orientation	T > S0.1
Phase enc. dir.	A >> P
FoV read	340 mm
FoV phase	75.0 %
Slice thickness	5.0 mm
TR	30.78 ms
Multi-slice mode	Sequential
Series	Ascending
Concatenations	1

Geometry - AutoAlign

Slice group	1
Position	L5.6 P35.5 H85.1 mm
Orientation	T > S0.1
Phase enc. dir.	A >> P
AutoAlign	---
Initial Position	L5.6 P35.5 H85.1
Phase	35.5 mm
Read	-5.8 mm
Shift	85.0 mm
Initial Rotation	0.00 deg
Initial Orientation	T > S
T > S	0.1
> C	0.0

Geometry - Saturation

Wrap-up Magn.	None
Special sat.	None

Geometry - Navigator

Geometry - Tim Planning Suite

Set-n-Go Protocol	Off
Table position	H
Table position	85 mm
Inline Composing	Off

© 2025 Steffen Johansson R et al. JAMA Network Open.

System - Miscellaneous

Positioning mode	ISO
Table position	H
Table position	85 mm
MSMA	S - C - T
Sagittal	R >> L
Coronal	A >> P
Transversal	F >> H
Coil Combine Mode	Sum of Squares
Matrix Optimization	Off
Coil Focus	Flat
AutoAlign	---
Coil Select Mode	Default

System - Adjustments

B0 Shim mode	Tune up
Adjust with body coil	Off
Confirm freq. adjustment	Off
Assume Dominant Fat	Off
Assume Silicone	Off
Adjustment Tolerance	Auto

System - Adjust Volume

Position	Isocenter
Orientation	Transversal
Rotation	0.00 deg
A >> P	263 mm
R >> L	350 mm
F >> H	350 mm
Reset	Off

System - Tx/Rx

Frequency 1H	63.672141 MHz
Correction factor	1
Gain	High
Img. Scale Cor.	1.000
Reset	Off
? Ref. amplitude 1H	0.000 V

Physio - Signal1

1st Signal/Mode	ECG/Retro
Average cycle	430 ± 159 ms
Average cycle	No Signal ms
Calculated phases	35
TR	30.78 ms
Concatenations	1
Segments	3
Arrhythmia detection	None

Physio - PACE

Resp. control	Off
Concatenations	1

Angio - Common

Flow mode	Single dir.
Encodings	1
Velocity enc.	100 cm/s
Direction	Through plane
Rephased images	On
Magnitude images	On
Magnitude sum	Off
Phase images	On

Angio - Inline

Subtract	Off
Measurements	1
StdDev	Off
Save original images	On

Angio - Cardiac

Inline Evaluation	Off
TE	2.83 ms
TR	30.78 ms
Save original images	On

Angio - MIP

MIP-Sag	Off
MIP-Cor	Off
MIP-Tra	Off
MIP-Time	Off
Save original images	On

Angio - Composing

Inline Composing	Off
Distortion Corr.	On
Mode	2D
Unfiltered images	Off

Sequence - Part 1

Introduction	Off
Dimension	2D
Reordering	Linear
Asymmetric echo	Strong
Flow comp.	Yes
Optimization	Min. TE TR
Multi-slice mode	Sequential
Echo spacing	5.1 ms
Sequence type	Gre
Bandwidth	454 Hz/Px

Sequence - Part 2

Define	Segments
Segments	3
RF pulse type	Fast
Gradient mode	Normal
Excitation	Slice-sel.
Flip angle mode	Constant
RF spoiling	On
Phase Enc. Rewinder	On
Cine	On

Sequence - Assistant

Mode	Off
Allowed delay	0 s

© 2025 Steffen Johansson R et al. JAMA Network Open.

\\USER\Kliniska hjartan\Hjärtprotokollet\COVID 6m\2D_Flow_mPAP_CS_FB
TA: 1:22 PM: ISO Voxel size: 1.8×1.8×6.0 mmPAT: 6.3 Rel. SNR: 1.00 : flWIP_r

Properties

Prio recon	Off
Load images to viewer	On
Inline movie	Off
Auto store images	On
Load images to stamp segments	Off Load
images to graphic segments	Off Auto
open inline display	Off
Auto close inline display	Off
Start measurement without further preparation	Off
Wait for user to start	Off
Start measurements	Single measurement

Routine

Slice group	1
Slices	10
Dist. factor	0 %
Position	Isocenter
Orientation	Sagittal
Phase enc. dir.	A >> P
AutoAlign	---
Phase oversampling	0 %
FoV read	340 mm
FoV phase	81.3 %
Slice thickness	6.0 mm
TR	75.24 ms
TE	4.10 ms
Averages	3
Concatenations	10
Filter	Distortion Corr.(2D), Prescan Normalize
Coil elements	BO1-3;SP1-3

Contrast - Common

TR	75.24 ms
TE	4.10 ms
TD	0 ms
Flip angle	15 deg
Wrap-up Magn.	None

Contrast - Dynamic

Averages	3
Averaging mode	Long term
Reconstruction	Magnitude
Measurements	1
Multiple series	Off

Resolution - Common

FoV read	340 mm
FoV phase	81.3 %
Slice thickness	6.0 mm
Base resolution	192
Phase resolution	72 %
Phase partial Fourier	Off
Trajectory	Cartesian
Interpolation	Off

Resolution - iPAT

PAT mode	None

Resolution - Filter Image

Image Filter	Off
Distortion Corr.	On
Mode	2D
Unfiltered images	Off
Prescan Normalize	On
Unfiltered images	Off
Normalize	Off
B1 filter	Off

Resolution - Filter Rawdata

Raw filter	Off
Elliptical filter	Off
POCS	Off

Geometry - Common

Slice group	1
Slices	10
Dist. factor	0 %
Position	Isocenter
Orientation	Sagittal
Phase enc. dir.	A >> P
FoV read	340 mm
FoV phase	81.3 %
Slice thickness	6.0 mm
TR	75.24 ms
Multi-slice mode	Sequential
Series	Ascending
Concatenations	10

Geometry - AutoAlign

Slice group	1
Position	Isocenter
Orientation	Sagittal
Phase enc. dir.	A >> P
AutoAlign	---
Initial Position	Isocenter
Phase	0.0 mm
Read	0.0 mm
Shift	0.0 mm
Initial Rotation	0.00 deg
Initial Orientation	Sagittal

Geometry - Saturation

Wrap-up Magn.	None
Special sat.	None

Geometry - Navigator

Geometry - Tim Planning Suite

Set-n-Go Protocol	Off
Table position	H
Table position	0 mm
Inline Composing	Off

System - Miscellaneous

Positioning mode	ISO
Table position	H
Table position	0 mm
MSMA	S - C - T
Sagittal	R >> L

© 2025 Steffen Johansson R et al. JAMA Network Open.

System - Miscellaneous

Coronal	A >> P
Transversal	F >> H
Coil Combine Mode	Adaptive Combine
Matrix Optimization	Off
Coil Focus	Flat
AutoAlign	---
Coil Select Mode	Default

System - Adjustments

B0 Shim mode	Cardiac
Adjust with body coil	Off
Confirm freq. adjustment	Off
Assume Dominant Fat	Off
Assume Silicone	Off
Adjustment Tolerance	Auto

System - Adjust Volume

Position	Isocenter
Orientation	Sagittal
Rotation	0.00 deg
A >> P	277 mm
F >> H	340 mm
R >> L	60 mm
Reset	Off

System - Tx/Rx

Frequency 1H	63.672141 MHz
Correction factor	1
Gain	High
Img. Scale Cor.	1.000
Reset	Off
? Ref. amplitude 1H	0.000 V

Physio - Signal1

1st Signal/Mode	ECG/Retro
Average cycle	430 ± 159 ms
Average cycle	No Signal ms
Calculated phases	20
TR	75.24 ms
Concatenations	10
Segments	3
Arrhythmia detection	None

Physio - PACE

Resp. control	Off
Concatenations	10

Angio - Common

Flow mode	Single vel.
Encodings	3
Velocity enc.	90 cm/s
Direction 1	Through plane
Direction 2	A >> P
Direction 3	F >> H
Rephased images	On
Magnitude images	Off
Magnitude sum	Off
Phase images	On

Angio - Inline

Subtract	Off
Measurements	1
StdDev	Off

Angio - Inline

Save original images	On

Angio - Cardiac

Inline Evaluation	Off
Contrasts	1
TE	4.10 ms
TR	75.24 ms
Save original images	On

Angio - MIP

MIP-Sag	Off
MIP-Cor	Off
MIP-Tra	Off
MIP-Time	Off
Save original images	On

Angio - Composing

Inline Composing	Off
Distortion Corr.	On
Mode	2D
Unfiltered images	Off

Sequence - Part 1

Introduction	Off
Dimension	2D
Reordering	Linear
Asymmetric echo	Off
Contrasts	1
Flow comp.	Yes
Optimization	Min. TR
Multi-slice mode	Sequential
Echo spacing	6.3 ms
Sequence type	Gre
Bandwidth	449 Hz/Px

Sequence - Part 2

Define	Segments
Segments	3
RF pulse type	Normal
Gradient mode	Fast
Excitation	Slice-sel.
Flip angle mode	Constant
RF spoiling	On
Phase Enc. Rewinder	On
Cine	On

Sequence - Special

Unfold Multi-Venc	Off
#k-center interp.	-1
ICE Recon. Method	IceCSensing
ReconMode	3
Iterations	40
Acc. Factor A	3
Acc. Factor B	11
Ref. Scans	0
Part. Fourier	0
n.a.	0
CSM Mode	3
Pairing	1
Spatial Reg.	0.0003
Temporal Reg.	0.0010
ICE Recon. Device	Automatic
Shared-Vel. Enc	Off

© 2025 Steffen Johansson R et al. JAMA Network Open.

Sequence - Assistant

Mode	Off
Allowed delay	5 s

© 2025 Steffen Johansson R et al. JAMA Network Open.

\\USER\Kliniska hjartan\Hjärtprotokollet\COVID 6m\SSFP_Perf_MBF_AI REST
TA: 0:52 PM: FIX Voxel size: 1.9×1.9×8.0 mmPAT: 3 Rel. SNR: 1.00 : tfi

Properties

Prio recon	Off
Load images to viewer	Off
Inline movie	Off
Auto store images	On
Load images to stamp segments	Off Load
images to graphic segments	On Auto
open inline display	Off
Auto close inline display	Off
Start measurement without further preparation	Off
Wait for user to start	Off
Start measurements	Single measurement

Routine

Slice group	1
Slices	3
Dist. factor	100 %
Position	Isocenter
Orientation	Transversal
Phase enc. dir.	A >> P
AutoAlign	---
Phase oversampling	0 %
FoV read	360 mm
FoV phase	75.0 %
Slice thickness	8.0 mm
TR	142.00 ms
TE	1.04 ms
Averages	1
Concatenations	1
Filter	Distortion Corr.(2D)
Coil elements	BO1-3;SP3,4

Contrast - Common

TR	142.00 ms
TE	1.04 ms
Magn. preparation	Non-sel. SR perf
TI	105 ms
Flip angle	50 deg
Fat suppr.	Fat sat.
Wrap-up Magn.	None

Contrast - Dynamic

Averages	1
Averaging mode	Long term
Reconstruction	Magnitude
Measurements	60
Pause after meas. 1	0.0 s
Pause after meas. 2	0.0 s
Pause after meas. 3	0.0 s
Pause after meas. 4	0.0 s
Pause after meas. 5	0.0 s
Pause after meas. 6	0.0 s
Pause after meas. 7	0.0 s
Pause after meas. 8	0.0 s
Pause after meas. 9	0.0 s
Pause after meas. 10	0.0 s
Pause after meas. 11	0.0 s
Pause after meas. 12	0.0 s
Pause after meas. 13	0.0 s
Pause after meas. 14	0.0 s

Contrast - Dynamic

Pause after meas. 15	0.0 s
Pause after meas. 16	0.0 s
Pause after meas. 17	0.0 s
Pause after meas. 18	0.0 s
Pause after meas. 19	0.0 s
Pause after meas. 20	0.0 s
Pause after meas. 21	0.0 s
Pause after meas. 22	0.0 s
Pause after meas. 23	0.0 s
Pause after meas. 24	0.0 s
Pause after meas. 25	0.0 s
Pause after meas. 26	0.0 s
Pause after meas. 27	0.0 s
Pause after meas. 28	0.0 s
Pause after meas. 29	0.0 s
Pause after meas. 30	0.0 s
Pause after meas. 31	0.0 s
Pause after meas. 32	0.0 s
Pause after meas. 33	0.0 s
Pause after meas. 34	0.0 s
Pause after meas. 35	0.0 s
Pause after meas. 36	0.0 s
Pause after meas. 37	0.0 s
Pause after meas. 38	0.0 s
Pause after meas. 39	0.0 s
Pause after meas. 40	0.0 s
Pause after meas. 41	0.0 s
Pause after meas. 42	0.0 s
Pause after meas. 43	0.0 s
Pause after meas. 44	0.0 s
Pause after meas. 45	0.0 s
Pause after meas. 46	0.0 s
Pause after meas. 47	0.0 s
Pause after meas. 48	0.0 s
Pause after meas. 49	0.0 s
Pause after meas. 50	0.0 s
Pause after meas. 51	0.0 s
Pause after meas. 52	0.0 s
Pause after meas. 53	0.0 s
Pause after meas. 54	0.0 s
Pause after meas. 55	0.0 s
Pause after meas. 56	0.0 s
Pause after meas. 57	0.0 s
Pause after meas. 58	0.0 s
Pause after meas. 59	0.0 s
Proton Dens. Maps	3
Multiple series	Off

Resolution - Common

FoV read	360 mm
FoV phase	75.0 %
Slice thickness	8.0 mm
Base resolution	192
Phase resolution	77 %
Phase partial Fourier	6/8
Trajectory	Cartesian
Interpolation	Off

Resolution - iPAT

PAT mode	GRAPPA

© 2025 Steffen Johansson R et al. JAMA Network Open.

Resolution - iPAT

Accel. factor PE	3
Reference scan mode	T-PAT

Resolution - Filter Image

Image Filter	Off
Distortion Corr.	On
Mode	2D
Unfiltered images	Off
Prescan Normalize	Off
Normalize	Off
B1 filter	Off

Resolution - Filter Rawdata

Raw filter	Off
Elliptical filter	Off
POCS	Off

Geometry - Common

Slice group	1
Slices	3
Dist. factor	100 %
Position	Isocenter
Orientation	Transversal
Phase enc. dir.	A >> P
FoV read	360 mm
FoV phase	75.0 %
Slice thickness	8.0 mm
TR	142.00 ms
Multi-slice mode	Single shot
Series	Ascending
Concatenations	1

Geometry - AutoAlign

Slice group	1
Position	Isocenter
Orientation	Transversal
Phase enc. dir.	A >> P
AutoAlign	---
Initial Position	Isocenter
Phase	0.0 mm
Read	0.0 mm
Shift	0.0 mm
Initial Rotation	0.00 deg
Initial Orientation	Transversal

Geometry - Saturation

Fat suppr.	Fat sat.
Wrap-up Magn.	None
Special sat.	None

Geometry - Navigator

Geometry - Tim Planning Suite

Set-n-Go Protocol	Off
Table position	H
Table position	0 mm
Inline Composing	Off

System - Miscellaneous

Positioning mode	FIX
Table position	H
Table position	0 mm
MSMA	S - C - T

System - Miscellaneous

Sagittal	R >> L
Coronal	A >> P
Transversal	F >> H
Coil Combine Mode	Sum of Squares
Save uncombined	Off
Matrix Optimization	Off
Coil Focus	Flat
AutoAlign	---
Coil Select Mode	Default

System - Adjustments

B0 Shim mode	Cardiac
Adjust with body coil	On
Confirm freq. adjustment	Off
Assume Dominant Fat	Off
Assume Silicone	Off
Adjustment Tolerance	Auto

System - Adjust Volume

Position	Isocenter
Orientation	Transversal
Rotation	0.00 deg
A >> P	270 mm
R >> L	360 mm
F >> H	40 mm
Reset	Off

System - Tx/Rx

Frequency 1H	63.672141 MHz
Correction factor	1
Gain	High
Img. Scale Cor.	1.000
Reset	Off
? Ref. amplitude 1H	0.000 V

Physio - Signal1

1st Signal/Mode	ECG/Trigger
Average cycle	430 ± 159 ms
Average cycle	No Signal ms
Captured cycle	430 ± 159 ms
Acquisition window	491 ms
Trigger pulse	1
Trigger delay	0 ms
TR	142.00 ms
Concatenations	1
Segments	37
Phases	1
Adaptive Triggering	Off

Physio - Cardiac

Tagging	None
Magn. preparation	Non-sel. SR perf
TI	105 ms
Fat suppr.	Fat sat.
Dark blood	Off
FoV read	360 mm
FoV phase	75.0 %
Phase resolution	77 %
Cine	Off
Trajectory	Cartesian
Dummy heartbeats	0
Motion Correction	None

© 2025 Steffen Johansson R et al. JAMA Network Open.

Physio - PACE

Resp. control	Off
Concatenations	1

Inline - Common

Subtract	Off
Measurements	60
StdDev	Off
Motion Correction	None
Save original images	On

Inline - Cardiac

Inline Evaluation	Off
Magn. preparation	Non-sel. SR perf
TE	1.04 ms
TR	142.00 ms
Motion Correction	None
Save original images	On

Inline - MIP

MIP-Sag	Off
MIP-Cor	Off
MIP-Tra	Off
MIP-Time	Off
Save original images	On

Inline - Composing

Inline Composing	Off
Distortion Corr.	On
Mode	2D
Unfiltered images	Off

Sequence - Part 1

Introduction	Off
Dimension	2D
Reordering	Linear
Asymmetric echo	Allowed
Optimization	Min. TE
Multi-slice mode	Single shot
Echo spacing	2.5 ms
Sequence type	Trufi
Bandwidth	1085 Hz/Px

Sequence - Part 2

Define	Shots
Shots per slice	1
EPI factor	1
Segments	37
Trufi delta freq.	0 Hz
RF pulse type	Fast
Gradient mode	Fast
Excitation	Slice-sel.
Flip angle mode	Constant
Cine	Off

Sequence - Special

AIF Images	On
AIF SR Preparation	SR_PERF
Scan	Test
Gadgetron IPR	PROTO2
Temporal Filter	On
Filter Method	Gaussian
Filter Strength	Medium

Sequence - Assistant

Mode	Min flip angle
Min flip angle	45 deg
Allowed delay	100 s

© 2025 Steffen Johansson R et al. JAMA Network Open.

\\USER\Kliniska hjartan\Hjärtprotokollet\COVID 6m\cine_2-chamber
TA: 3.9 s PM: FIX Voxel size: 1.4×1.4×8.0 mmPAT: 2 Rel. SNR: 1.00 : tfi

Properties

Prio recon	On
Load images to viewer	On
Inline movie	On
Auto store images	On
Load images to stamp segments On Load	
images to graphic segments On Auto	
open inline display	Off
Auto close inline display	Off
Start measurement without further preparation	Off
Wait for user to start	Off
Start measurements	Single measurement

Routine

Slice group	1
Slices	1
Dist. factor	20 %
Position	Isocenter
Orientation	Transversal
Phase enc. dir.	A >> P
AutoAlign	---
Phase oversampling	0 %
FoV read	360 mm
FoV phase	75.0 %
Slice thickness	8.0 mm
TR	37.05 ms
TE	1.19 ms
Averages	1
Concatenations	1
Filter	Distortion Corr.(2D), Prescan Normalize
Coil elements	BO1-3;SP2,3

Contrast - Common

TR	37.05 ms
TE	1.19 ms
Magn. preparation	None
Flip angle	68 deg
Fat suppr.	None
Wrap-up Magn.	Restore

Contrast - Dynamic

Averages	1
Averaging mode	Short term
Reconstruction	Magnitude
Measurements	1
Multiple series	Off

Resolution - Common

FoV read	360 mm
FoV phase	75.0 %
Slice thickness	8.0 mm
Base resolution	256
Phase resolution	75 %
Phase partial Fourier	Off
Trajectory	Cartesian
View sharing	Off
Interpolation	Off

Resolution - iPAT

PAT mode	GRAPPA
Accel. factor PE	2
Ref. lines PE	44
Reference scan mode	Integrated

Resolution - Filter Image

Image Filter	Off
Distortion Corr.	On
Mode	2D
Unfiltered images	Off
Prescan Normalize	On
Unfiltered images	Off
Normalize	Off
B1 filter	Off

Resolution - Filter Rawdata

Raw filter	Off
Elliptical filter	Off
POCS	Off

Geometry - Common

Slice group	1
Slices	1
Dist. factor	20 %
Position	Isocenter
Orientation	Transversal
Phase enc. dir.	A >> P
FoV read	360 mm
FoV phase	75.0 %
Slice thickness	8.0 mm
TR	37.05 ms
Multi-slice mode	Sequential
Series	Base To Apex
Concatenations	1

Geometry - AutoAlign

Slice group	1
Position	Isocenter
Orientation	Transversal
Phase enc. dir.	A >> P
AutoAlign	---
Initial Position	Isocenter
Phase	0.0 mm
Read	0.0 mm
Shift	0.0 mm
Initial Rotation	0.00 deg
Initial Orientation	Transversal

Geometry - Saturation

Fat suppr.	None
Wrap-up Magn.	Restore
Special sat.	None

Geometry - Navigator

Geometry - Tim Planning Suite

Set-n-Go Protocol	Off
Table position	H
Table position	0 mm
Inline Composing	Off

© 2025 Steffen Johansson R et al. JAMA Network Open.

System - Miscellaneous

Positioning mode	FIX
Table position	H
Table position	0 mm
MSMA	S - C - T
Sagittal	R >> L
Coronal	A >> P
Transversal	F >> H
Coil Combine Mode	Sum of Squares
Save uncombined	Off
Matrix Optimization	Off
Coil Focus	Flat
AutoAlign	---
Coil Select Mode	Default

System - Adjustments

B0 Shim mode	Tune up
Adjust with body coil	Off
Confirm freq. adjustment	Off
Assume Dominant Fat	Off
Assume Silicone	Off
Adjustment Tolerance	Auto

System - Adjust Volume

Position	Isocenter
Orientation	Transversal
Rotation	0.00 deg
A >> P	263 mm
R >> L	350 mm
F >> H	350 mm
Reset	Off

System - Tx/Rx

Frequency 1H	63.672141 MHz
Correction factor	1
Gain	High
Img. Scale Cor.	1.000
Reset	Off
? Ref. amplitude 1H	0.000 V

Physio - Signal1

1st Signal/Mode	ECG/Retro
Average cycle	430 ± 159 ms
Average cycle	No Signal ms
Calculated phases	25
TR	37.05 ms
Concatenations	1
Segments	13
Arrhythmia detection	None

Physio - Cardiac

Tagging	None
Magn. preparation	None
Fat suppr.	None
Dark blood	Off
FoV read	360 mm
FoV phase	75.0 %
Phase resolution	75 %
Cine	On
Trajectory	Cartesian
View sharing	Off
Dummy heartbeats	1

Physio - PACE

Physio - PACE

Concatenations	1

Inline - Common

Subtract	Off
Measurements	1
StdDev	Off
Save original images	On

Inline - Cardiac

Inline Evaluation	Ventricular Function
Magn. preparation	None
Contrasts	1
TE	1.19 ms
TR	37.05 ms
Save original images	On

Inline - MIP

MIP-Sag	Off
MIP-Cor	Off
MIP-Tra	Off
MIP-Time	Off
Save original images	On

Inline - Composing

Inline Composing	Off
Distortion Corr.	On
Mode	2D
Unfiltered images	Off

Sequence - Part 1

Introduction	Off
Dimension	2D
Reordering	Linear
Asymmetric echo	Weak
Contrasts	1
Optimization	Min. TE TR
Multi-slice mode	Sequential
Echo spacing	2.9 ms
Sequence type	Trufi
Bandwidth	930 Hz/Px

Sequence - Part 2

Define	Segments
Segments	13
Trufi delta freq.	0 Hz
RF pulse type	Fast
Gradient mode	Fast
Excitation	Slice-sel.
Flip angle mode	Constant
Cine	On

Sequence - Assistant

Mode	Min flip angle
Min flip angle	50 deg
Allowed delay	0 s

© 2025 Steffen Johansson R et al. JAMA Network Open.

Resp. control	Off

© 2025 Steffen Johansson R et al. JAMA Network Open.

\\USER\Kliniska hjartan\Hjärtprotokollet\COVID 6m\cine_3-chamber
TA: 3.9 s PM: FIX Voxel size: 1.4×1.4×8.0 mmPAT: 2 Rel. SNR: 1.00 : tfi

Properties

Prio recon	On
Load images to viewer	On
Inline movie	On
Auto store images	On
Load images to stamp segments	On Load
images to graphic segments	On Auto
open inline display	Off
Auto close inline display	Off
Start measurement without further preparation	Off
Wait for user to start	Off
Start measurements	Single measurement

Routine

Slice group	1
Slices	1
Dist. factor	20 %
Position	Isocenter
Orientation	Transversal
Phase enc. dir.	A >> P
AutoAlign	---
Phase oversampling	0 %
FoV read	360 mm
FoV phase	75.0 %
Slice thickness	8.0 mm
TR	37.05 ms
TE	1.19 ms
Averages	1
Concatenations	1
Filter	Distortion Corr.(2D), Prescan Normalize
Coil elements	BO1-3;SP2,3

Contrast - Common

TR	37.05 ms
TE	1.19 ms
Magn. preparation	None
Flip angle	68 deg
Fat suppr.	None
Wrap-up Magn.	Restore

Contrast - Dynamic

Averages	1
Averaging mode	Short term
Reconstruction	Magnitude
Measurements	1
Multiple series	Off

Resolution - Common

FoV read	360 mm
FoV phase	75.0 %
Slice thickness	8.0 mm
Base resolution	256
Phase resolution	75 %
Phase partial Fourier	Off
Trajectory	Cartesian
View sharing	Off
Interpolation	Off

Resolution - iPAT

PAT mode	GRAPPA
Accel. factor PE	2
Ref. lines PE	44
Reference scan mode	Integrated

Resolution - Filter Image

Image Filter	Off
Distortion Corr.	On
Mode	2D
Unfiltered images	Off
Prescan Normalize	On
Unfiltered images	Off
Normalize	Off
B1 filter	Off

Resolution - Filter Rawdata

Raw filter	Off
Elliptical filter	Off
POCS	Off

Geometry - Common

Slice group	1
Slices	1
Dist. factor	20 %
Position	Isocenter
Orientation	Transversal
Phase enc. dir.	A >> P
FoV read	360 mm
FoV phase	75.0 %
Slice thickness	8.0 mm
TR	37.05 ms
Multi-slice mode	Sequential
Series	Base To Apex
Concatenations	1

Geometry - AutoAlign

Slice group	1
Position	Isocenter
Orientation	Transversal
Phase enc. dir.	A >> P
AutoAlign	---
Initial Position	Isocenter
Phase	0.0 mm
Read	0.0 mm
Shift	0.0 mm
Initial Rotation	0.00 deg
Initial Orientation	Transversal

Geometry - Saturation

Fat suppr.	None
Wrap-up Magn.	Restore
Special sat.	None

Geometry - Navigator

Geometry - Tim Planning Suite

Set-n-Go Protocol	Off
Table position	H
Table position	0 mm
Inline Composing	Off

© 2025 Steffen Johansson R et al. JAMA Network Open.

System - Miscellaneous

Positioning mode	FIX
Table position	H
Table position	0 mm
MSMA	S - C - T
Sagittal	R >> L
Coronal	A >> P
Transversal	F >> H
Coil Combine Mode	Sum of Squares
Save uncombined	Off
Matrix Optimization	Off
Coil Focus	Flat
AutoAlign	---
Coil Select Mode	Default

System - Adjustments

B0 Shim mode	Tune up
Adjust with body coil	Off
Confirm freq. adjustment	Off
Assume Dominant Fat	Off
Assume Silicone	Off
Adjustment Tolerance	Auto

System - Adjust Volume

Position	Isocenter
Orientation	Transversal
Rotation	0.00 deg
A >> P	263 mm
R >> L	350 mm
F >> H	350 mm
Reset	Off

System - Tx/Rx

Frequency 1H	63.672141 MHz
Correction factor	1
Gain	High
Img. Scale Cor.	1.000
Reset	Off
? Ref. amplitude 1H	0.000 V

Physio - Signal1

1st Signal/Mode	ECG/Retro
Average cycle	430 ± 159 ms
Average cycle	No Signal ms
Calculated phases	25
TR	37.05 ms
Concatenations	1
Segments	13
Arrhythmia detection	None

Physio - Cardiac

Tagging	None
Magn. preparation	None
Fat suppr.	None
Dark blood	Off
FoV read	360 mm
FoV phase	75.0 %
Phase resolution	75 %
Cine	On
Trajectory	Cartesian
View sharing	Off
Dummy heartbeats	1

Physio - PACE

Physio - PACE

Concatenations	1

Inline - Common

Subtract	Off
Measurements	1
StdDev	Off
Save original images	On

Inline - Cardiac

Inline Evaluation	Ventricular Function
Magn. preparation	None
Contrasts	1
TE	1.19 ms
TR	37.05 ms
Save original images	On

Inline - MIP

MIP-Sag	Off
MIP-Cor	Off
MIP-Tra	Off
MIP-Time	Off
Save original images	On

Inline - Composing

Inline Composing	Off
Distortion Corr.	On
Mode	2D
Unfiltered images	Off

Sequence - Part 1

Introduction	Off
Dimension	2D
Reordering	Linear
Asymmetric echo	Weak
Contrasts	1
Optimization	Min. TE TR
Multi-slice mode	Sequential
Echo spacing	2.9 ms
Sequence type	Trufi
Bandwidth	930 Hz/Px

Sequence - Part 2

Define	Segments
Segments	13
Trufi delta freq.	0 Hz
RF pulse type	Fast
Gradient mode	Fast
Excitation	Slice-sel.
Flip angle mode	Constant
Cine	On

Sequence - Assistant

Mode	Min flip angle
Min flip angle	50 deg
Allowed delay	0 s

© 2025 Steffen Johansson R et al. JAMA Network Open.

Resp. control	Off

© 2025 Steffen Johansson R et al. JAMA Network Open.

\\USER\Kliniska hjartan\Hjärtprotokollet\COVID 6m\cine_4-chamber
TA: 3.9 s PM: FIX Voxel size: 1.5×1.5×8.0 mmPAT: 2 Rel. SNR: 1.00 : tfi

Properties

Prio recon	On
Load images to viewer	On
Inline movie	On
Auto store images	On
Load images to stamp segments	On Load
images to graphic segments	On Auto
open inline display	Off
Auto close inline display	Off
Start measurement without further preparation	Off
Wait for user to start	Off
Start measurements	Single measurement

Routine

Slice group	1
Slices	1
Dist. factor	20 %
Position	Isocenter
Orientation	Transversal
Phase enc. dir.	A >> P
AutoAlign	---
Phase oversampling	0 %
FoV read	380 mm
FoV phase	84.4 %
Slice thickness	8.0 mm
TR	36.14 ms
TE	1.16 ms
Averages	1
Concatenations	1
Filter	Distortion Corr.(2D), Prescan Normalize
Coil elements	BO1-3;SP1-4

Contrast - Common

TR	36.14 ms
TE	1.16 ms
Magn. preparation	None
Flip angle	68 deg
Fat suppr.	None
Wrap-up Magn.	Restore

Contrast - Dynamic

Averages	1
Averaging mode	Short term
Reconstruction	Magnitude
Measurements	1
Multiple series	Off

Resolution - Common

FoV read	380 mm
FoV phase	84.4 %
Slice thickness	8.0 mm
Base resolution	256
Phase resolution	66 %
Phase partial Fourier	Off
Trajectory	Cartesian
View sharing	Off
Interpolation	Off

Resolution - iPAT

PAT mode	GRAPPA
Accel. factor PE	2
Ref. lines PE	44
Reference scan mode	Integrated

Resolution - Filter Image

Image Filter	Off
Distortion Corr.	On
Mode	2D
Unfiltered images	Off
Prescan Normalize	On
Unfiltered images	Off
Normalize	Off
B1 filter	Off

Resolution - Filter Rawdata

Raw filter	Off
Elliptical filter	Off
POCS	Off

Geometry - Common

Slice group	1
Slices	1
Dist. factor	20 %
Position	Isocenter
Orientation	Transversal
Phase enc. dir.	A >> P
FoV read	380 mm
FoV phase	84.4 %
Slice thickness	8.0 mm
TR	36.14 ms
Multi-slice mode	Sequential
Series	Base To Apex
Concatenations	1

Geometry - AutoAlign

Slice group	1
Position	Isocenter
Orientation	Transversal
Phase enc. dir.	A >> P
AutoAlign	---
Initial Position	Isocenter
Phase	0.0 mm
Read	0.0 mm
Shift	0.0 mm
Initial Rotation	0.00 deg
Initial Orientation	Transversal

Geometry - Saturation

Fat suppr.	None
Wrap-up Magn.	Restore
Special sat.	None

Geometry - Navigator

Geometry - Tim Planning Suite

Set-n-Go Protocol	Off
Table position	H
Table position	0 mm
Inline Composing	Off

© 2025 Steffen Johansson R et al. JAMA Network Open.

System - Miscellaneous

Positioning mode	FIX
Table position	H
Table position	0 mm
MSMA	S - C - T
Sagittal	R >> L
Coronal	A >> P
Transversal	F >> H
Coil Combine Mode	Sum of Squares
Save uncombined	Off
Matrix Optimization	Off
Coil Focus	Flat
AutoAlign	---
Coil Select Mode	Default

System - Adjustments

B0 Shim mode	Cardiac
Adjust with body coil	Off
Confirm freq. adjustment	Off
Assume Dominant Fat	Off
Assume Silicone	Off
Adjustment Tolerance	Auto

System - Adjust Volume

! Position	Isocenter
! Orientation	Transversal
! Rotation	0.00 deg
! A >> P	270 mm
! R >> L	360 mm
! F >> H	8 mm
Reset	Off

System - Tx/Rx

Frequency 1H	63.672141 MHz
Correction factor	1
Gain	High
Img. Scale Cor.	1.000
Reset	Off
? Ref. amplitude 1H	0.000 V

Physio - Signal1

1st Signal/Mode	ECG/Retro
Average cycle	430 ± 159 ms
Average cycle	No Signal ms
Calculated phases	25
TR	36.14 ms
Concatenations	1
Segments	13
Arrhythmia detection	None

Physio - Cardiac

Tagging	None
Magn. preparation	None
Fat suppr.	None
Dark blood	Off
FoV read	380 mm
FoV phase	84.4 %
Phase resolution	66 %
Cine	On
Trajectory	Cartesian
View sharing	Off
Dummy heartbeats	1

Physio - PACE

Physio - PACE

Concatenations	1

Inline - Common

Subtract	Off
Measurements	1
StdDev	Off
Save original images	On

Inline - Cardiac

Inline Evaluation	Ventricular Function
Magn. preparation	None
Contrasts	1
TE	1.16 ms
TR	36.14 ms
Save original images	On

Inline - MIP

MIP-Sag	Off
MIP-Cor	Off
MIP-Tra	Off
MIP-Time	Off
Save original images	On

Inline - Composing

Inline Composing	Off
Distortion Corr.	On
Mode	2D
Unfiltered images	Off

Sequence - Part 1

Introduction	Off
Dimension	2D
Reordering	Linear
Asymmetric echo	Weak
Contrasts	1
Optimization	Min. TE TR
Multi-slice mode	Sequential
Echo spacing	2.8 ms
Sequence type	Trufi
Bandwidth	930 Hz/Px

Sequence - Part 2

Define	Segments
Segments	13
Trufi delta freq.	0 Hz
RF pulse type	Fast
Gradient mode	Fast
Excitation	Slice-sel.
Flip angle mode	Constant
Cine	On

Sequence - Assistant

Mode	Min flip angle
Min flip angle	50 deg
Allowed delay	0 s

© 2025 Steffen Johansson R et al. JAMA Network Open.

Resp. control	Off

© 2025 Steffen Johansson R et al. JAMA Network Open.

\\USER\Kliniska hjartan\Hjärtprotokollet\COVID 6m\cine_short-axis
TA: 1:18 PM: FIX Voxel size: 1.4×1.4×8.0 mmPAT: 2 Rel. SNR: 1.00 : tfi

Properties

Prio recon	Off
Load images to viewer	On
Inline movie	On
Auto store images	On
Load images to stamp segments	On Load
images to graphic segments	On Auto
open inline display	Off
Auto close inline display	Off
Start measurement without further preparation	Off
Wait for user to start	Off
Start measurements	Single measurement

Routine

Slice group	1
Slices	11
Dist. factor	20 %
Position	Isocenter
Orientation	Transversal
Phase enc. dir.	A >> P
AutoAlign	---
Phase oversampling	0 %
FoV read	360 mm
FoV phase	75.0 %
Slice thickness	8.0 mm
TR	42.75 ms
TE	1.19 ms
Averages	1
Concatenations	11
Filter	Distortion Corr.(2D), Prescan Normalize
Coil elements	BO1-3;SP1-3

Contrast - Common

TR	42.75 ms
TE	1.19 ms
Magn. preparation	None
Flip angle	67 deg
Fat suppr.	None
Wrap-up Magn.	Restore

Contrast - Dynamic

Averages	1
Averaging mode	Short term
Reconstruction	Magnitude
Measurements	1
Multiple series	Off

Resolution - Common

FoV read	360 mm
FoV phase	75.0 %
Slice thickness	8.0 mm
Base resolution	256
Phase resolution	75 %
Phase partial Fourier	Off
Trajectory	Cartesian
View sharing	Off
Interpolation	Off

Resolution - iPAT

PAT mode	GRAPPA
Accel. factor PE	2
Ref. lines PE	36
Reference scan mode	Integrated

Resolution - Filter Image

Image Filter	Off
Distortion Corr.	On
Mode	2D
Unfiltered images	Off
Prescan Normalize	On
Unfiltered images	Off
Normalize	Off
B1 filter	Off

Resolution - Filter Rawdata

Raw filter	Off
Elliptical filter	Off
POCS	Off

Geometry - Common

Slice group	1
Slices	11
Dist. factor	20 %
Position	Isocenter
Orientation	Transversal
Phase enc. dir.	A >> P
FoV read	360 mm
FoV phase	75.0 %
Slice thickness	8.0 mm
TR	42.75 ms
Multi-slice mode	Sequential
Series	Base To Apex
Concatenations	11

Geometry - AutoAlign

Slice group	1
Position	Isocenter
Orientation	Transversal
Phase enc. dir.	A >> P
AutoAlign	---
Initial Position	Isocenter
Phase	0.0 mm
Read	0.0 mm
Shift	0.0 mm
Initial Rotation	0.00 deg
Initial Orientation	Transversal

Geometry - Saturation

Fat suppr.	None
Wrap-up Magn.	Restore
Special sat.	None

Geometry - Navigator

Geometry - Tim Planning Suite

Set-n-Go Protocol	Off
Table position	H
Table position	0 mm
Inline Composing	Off

© 2025 Steffen Johansson R et al. JAMA Network Open.

System - Miscellaneous

Positioning mode	FIX
Table position	H
Table position	0 mm
MSMA	S - C - T
Sagittal	R >> L
Coronal	A >> P
Transversal	F >> H
Coil Combine Mode	Sum of Squares
Save uncombined	Off
Matrix Optimization	Off
Coil Focus	Flat
AutoAlign	---
Coil Select Mode	Default

System - Adjustments

B0 Shim mode	Cardiac
Adjust with body coil	Off
Confirm freq. adjustment	Off
Assume Dominant Fat	Off
Assume Silicone	Off
Adjustment Tolerance	Auto

System - Adjust Volume

! Position	Isocenter
! Orientation	Transversal
! Rotation	0.00 deg
! A >> P	293 mm
! R >> L	390 mm
! F >> H	48 mm
Reset	Off

System - Tx/Rx

Frequency 1H	63.672141 MHz
Correction factor	1
Gain	High
Img. Scale Cor.	1.000
Reset	Off
? Ref. amplitude 1H	0.000 V

Physio - Signal1

1st Signal/Mode	ECG/Retro
Average cycle	430 ± 159 ms
Average cycle	No Signal ms
Calculated phases	25
TR	42.75 ms
Concatenations	11
Segments	15
Arrhythmia detection	None

Physio - Cardiac

Tagging	None
Magn. preparation	None
Fat suppr.	None
Dark blood	Off
FoV read	360 mm
FoV phase	75.0 %
Phase resolution	75 %
Cine	On
Trajectory	Cartesian
View sharing	Off
Dummy heartbeats	1

Physio - PACE

Physio - PACE

Concatenations	11

Inline - Common

Subtract	Off
Measurements	1
StdDev	Off
Save original images	On

Inline - Cardiac

Inline Evaluation	Ventricular Function
Magn. preparation	None
Contrasts	1
TE	1.19 ms
TR	42.75 ms
Save original images	On

Inline - MIP

MIP-Sag	Off
MIP-Cor	Off
MIP-Tra	Off
MIP-Time	Off
Save original images	On

Inline - Composing

Inline Composing	Off
Distortion Corr.	On
Mode	2D
Unfiltered images	Off

Sequence - Part 1

Introduction	Off
Dimension	2D
Reordering	Linear
Asymmetric echo	Weak
Contrasts	1
Optimization	Min. TE TR
Multi-slice mode	Sequential
Echo spacing	2.9 ms
Sequence type	Trufi
Bandwidth	930 Hz/Px

Sequence - Part 2

Define	Segments
Segments	15
Trufi delta freq.	0 Hz
RF pulse type	Fast
Gradient mode	Fast
Excitation	Slice-sel.
Flip angle mode	Constant
Cine	On

Sequence - Assistant

Mode	Min flip angle
Min flip angle	50 deg
Allowed delay	0 s

© 2025 Steffen Johansson R et al. JAMA Network Open.

Resp. control	Breath-hold

© 2025 Steffen Johansson R et al. JAMA Network Open.

\\USER\Kliniska hjartan\Hjärtprotokollet\COVID 6m\BEAT_FQ_SWIG_MITRAL_TP_100
TA: 5.6 s PM: ISO Voxel size: 2.7×2.7×6.0 mmPAT: Off Rel. SNR: 1.00 : fl_r

Properties

Prio recon	Off
Load images to viewer	On
Inline movie	Off
Auto store images	On
Load images to stamp segments	Off Load
images to graphic segments	Off Auto
open inline display	Off
Auto close inline display	Off
Start measurement without further preparation	Off
Wait for user to start	On
Start measurements	Single measurement

Routine

Slice group	1
Slices	1
Dist. factor	20 %
Position	Isocenter
Orientation	Transversal
Phase enc. dir.	A >> P
AutoAlign	---
Phase oversampling	0 %
FoV read	300 mm
FoV phase	100.0 %
Slice thickness	6.0 mm
TR	118.36 ms
TE	3.37 ms
Averages	1
Concatenations	1
Filter	Distortion Corr.(2D)
Coil elements	BO1-3;SP1-3

Contrast - Common

TR	118.36 ms
TE	3.37 ms
TD	0 ms
Flip angle	40 deg
Wrap-up Magn.	None

Contrast - Dynamic

Averages	1
Averaging mode	Short term
Reconstruction	Magnitude
Measurements	1
Multiple series	Off

Resolution - Common

FoV read	300 mm
FoV phase	100.0 %
Slice thickness	6.0 mm
Base resolution	112
Radial views	132
Radial interleaves	1
Trajectory	Radial
View sharing	Off
Interpolation	Off

Resolution - iPAT

PAT mode	None

Resolution - Filter Image

Image Filter	Off
Distortion Corr.	On
Mode	2D
Unfiltered images	Off
Prescan Normalize	Off
Normalize	Off
B1 filter	Off

Resolution - Filter Rawdata

Raw filter	Off
Elliptical filter	Off
POCS	Off

Geometry - Common

Slice group	1
Slices	1
Dist. factor	20 %
Position	Isocenter
Orientation	Transversal
Phase enc. dir.	A >> P
FoV read	300 mm
FoV phase	100.0 %
Slice thickness	6.0 mm
TR	118.36 ms
Multi-slice mode	Sequential
Series	Interleaved
Concatenations	1

Geometry - AutoAlign

Slice group	1
Position	Isocenter
Orientation	Transversal
Phase enc. dir.	A >> P
AutoAlign	---
Initial Position	Isocenter
Phase	0.0 mm
Read	0.0 mm
Shift	0.0 mm
Initial Rotation	0.00 deg
Initial Orientation	Transversal

Geometry - Saturation

Wrap-up Magn.	None
Special sat.	None

Geometry - Navigator

Geometry - Tim Planning Suite

Set-n-Go Protocol	Off
Table position	H
Table position	0 mm
Inline Composing	Off

System - Miscellaneous

Positioning mode	ISO
Table position	H
Table position	0 mm
MSMA	S - C - T
Sagittal	R >> L
Coronal	A >> P

© 2025 Steffen Johansson R et al. JAMA Network Open.

System - Miscellaneous

Transversal	F >> H
Coil Combine Mode	Sum of Squares
Matrix Optimization	Off
Coil Focus	Flat
AutoAlign	---
Coil Select Mode	Default

System - Adjustments

B0 Shim mode	Cardiac
Adjust with body coil	Off
Confirm freq. adjustment	Off
Assume Dominant Fat	Off
Assume Silicone	Off
Adjustment Tolerance	Auto

System - Adjust Volume

! Position	Isocenter
! Orientation	Transversal
! Rotation	0.00 deg
! A >> P	500 mm
! R >> L	500 mm
! F >> H	6 mm
Reset	Off

System - Tx/Rx

Frequency 1H	63.672141 MHz
Correction factor	1
Gain	High
Img. Scale Cor.	1.000
Reset	Off
? Ref. amplitude 1H	0.000 V

Physio - Signal1

1st Signal/Mode	ECG/Retro
Average cycle	430 ± 159 ms
Average cycle	No Signal ms
Calculated phases	20
TR	118.36 ms
Concatenations	1
Segments	11
Arrhythmia detection	None

Physio - PACE

Resp. control	Off
Concatenations	1

Angio - Common

Flow mode	Single dir.
Encodings	1
Velocity enc.	100 cm/s
Direction	Through plane
Rephased images	On
Magnitude images	On
Magnitude sum	Off
Phase images	On

Angio - Inline

Subtract	Off
Measurements	1
StdDev	Off
Save original images	On

Angio - Cardiac

Inline Evaluation	Off
TE	3.37 ms
TR	118.36 ms
Save original images	On

Angio - MIP

MIP-Sag	Off
MIP-Cor	Off
MIP-Tra	Off
MIP-Time	Off
Save original images	On

Angio - Composing

Inline Composing	Off
Distortion Corr.	On
Mode	2D
Unfiltered images	Off

Sequence - Part 1

Introduction	Off
Dimension	2D
Asymmetric echo	Off
Flow comp.	Slice/Read
Optimization	Min. TE TR
Multi-slice mode	Sequential
Echo spacing	5.4 ms
Sequence type	Gre
Bandwidth	558 Hz/Px

Sequence - Part 2

Define	Segments
Shots per slice	12
Segments	11
RF pulse type	Normal
Gradient mode	Fast
Excitation	Slice-sel.
Flip angle mode	Constant
RF spoiling	On
Phase Enc. Rewinder	On
Cine	On

Sequence - Special

Vel Encoding	Symmetric
Radial ice program	IceProgramFire_SWIG_F(

Sequence - Assistant

Mode	Min flip angle
Min flip angle	15 deg
Allowed delay	5 s

© 2025 Steffen Johansson R et al. JAMA Network Open.

\\USER\Kliniska hjartan\Hjärtprotokollet\COVID 6m\BEAT_FQ_SWIG_MYO_TP_20
TA: 5.6 s PM: ISO Voxel size: 2.7×2.7×6.0 mmPAT: Off Rel. SNR: 1.00 : fl_r

Properties

Prio recon	Off
Load images to viewer	On
Inline movie	Off
Auto store images	On
Load images to stamp segments	Off Load
images to graphic segments	Off Auto
open inline display	Off
Auto close inline display	Off
Start measurement without further preparation	Off
Wait for user to start	Off
Start measurements	Single measurement

Routine

Slice group	1
Slices	1
Dist. factor	20 %
Position	Isocenter
Orientation	Transversal
Phase enc. dir.	A >> P
AutoAlign	---
Phase oversampling	0 %
FoV read	300 mm
FoV phase	100.0 %
Slice thickness	6.0 mm
TR	143.66 ms
TE	4.52 ms
Averages	1
Concatenations	1
Filter	Distortion Corr.(2D)
Coil elements	BO1-3;SP1-3

Contrast - Common

TR	143.66 ms
TE	4.52 ms
TD	0 ms
Flip angle	9 deg
Wrap-up Magn.	None

Contrast - Dynamic

Averages	1
Averaging mode	Short term
Reconstruction	Magnitude
Measurements	1
Multiple series	Off

Resolution - Common

FoV read	300 mm
FoV phase	100.0 %
Slice thickness	6.0 mm
Base resolution	112
Radial views	132
Radial interleaves	1
Trajectory	Radial
View sharing	Off
Interpolation	Off

Resolution - iPAT

PAT mode	None

Resolution - Filter Image

Image Filter	Off
Distortion Corr.	On
Mode	2D
Unfiltered images	Off
Prescan Normalize	Off
Normalize	Off
B1 filter	Off

Resolution - Filter Rawdata

Raw filter	Off
Elliptical filter	Off
POCS	Off

Geometry - Common

Slice group	1
Slices	1
Dist. factor	20 %
Position	Isocenter
Orientation	Transversal
Phase enc. dir.	A >> P
FoV read	300 mm
FoV phase	100.0 %
Slice thickness	6.0 mm
TR	143.66 ms
Multi-slice mode	Sequential
Series	Interleaved
Concatenations	1

Geometry - AutoAlign

Slice group	1
Position	Isocenter
Orientation	Transversal
Phase enc. dir.	A >> P
AutoAlign	---
Initial Position	Isocenter
Phase	0.0 mm
Read	0.0 mm
Shift	0.0 mm
Initial Rotation	0.00 deg
Initial Orientation	Transversal

Geometry - Saturation

Wrap-up Magn.	None
Special sat.	None

Geometry - Navigator

Geometry - Tim Planning Suite

Set-n-Go Protocol	Off
Table position	H
Table position	0 mm
Inline Composing	Off

System - Miscellaneous

Positioning mode	ISO
Table position	H
Table position	0 mm
MSMA	S - C - T
Sagittal	R >> L
Coronal	A >> P

© 2025 Steffen Johansson R et al. JAMA Network Open.

System - Miscellaneous

Transversal	F >> H
Coil Combine Mode	Sum of Squares
Matrix Optimization	Off
Coil Focus	Flat
AutoAlign	---
Coil Select Mode	Default

System - Adjustments

B0 Shim mode	Cardiac
Adjust with body coil	Off
Confirm freq. adjustment	Off
Assume Dominant Fat	Off
Assume Silicone	Off
Adjustment Tolerance	Auto

System - Adjust Volume

! Position	Isocenter
! Orientation	Transversal
! Rotation	0.00 deg
! A >> P	500 mm
! R >> L	500 mm
! F >> H	6 mm
Reset	Off

System - Tx/Rx

Frequency 1H	63.672141 MHz
Correction factor	1
Gain	High
Img. Scale Cor.	1.000
Reset	Off
? Ref. amplitude 1H	0.000 V

Physio - Signal1

1st Signal/Mode	ECG/Retro
Average cycle	430 ± 159 ms
Average cycle	No Signal ms
Calculated phases	20
TR	143.66 ms
Concatenations	1
Segments	11
Arrhythmia detection	None

Physio - PACE

Resp. control	Off
Concatenations	1

Angio - Common

Flow mode	Single dir.
Encodings	1
Velocity enc.	20 cm/s
Direction	Through plane
Rephased images	On
Magnitude images	On
Magnitude sum	Off
Phase images	On

Angio - Inline

Subtract	Off
Measurements	1
StdDev	Off
Save original images	On

Angio - Cardiac

Inline Evaluation	Off
TE	4.52 ms
TR	143.66 ms
Save original images	On

Angio - MIP

MIP-Sag	Off
MIP-Cor	Off
MIP-Tra	Off
MIP-Time	Off
Save original images	On

Angio - Composing

Inline Composing	Off
Distortion Corr.	On
Mode	2D
Unfiltered images	Off

Sequence - Part 1

Introduction	Off
Dimension	2D
Asymmetric echo	Off
Flow comp.	Slice/Read
Optimization	Min. TE TR
Multi-slice mode	Sequential
Echo spacing	6.5 ms
Sequence type	Gre
Bandwidth	558 Hz/Px

Sequence - Part 2

Define	Segments
Shots per slice	12
Segments	11
RF pulse type	Normal
Gradient mode	Fast
Excitation	Slice-sel.
Flip angle mode	Constant
RF spoiling	On
Phase Enc. Rewinder	On
Cine	On

Sequence - Special

Vel Encoding	Symmetric
Radial ice program	IceProgramFire_SWIG_F[illegible]

Sequence - Assistant

Mode	Min flip angle
Min flip angle	15 deg
Allowed delay	5 s

© 2025 Steffen Johansson R et al. JAMA Network Open.

\\USER\Kliniska hjartan\Hjärtprotokollet\COVID 6m\BEAT_FQ_SWIG_MITRAL_IP_100
TA: 5.6 s PM: ISO Voxel size: 2.7×2.7×6.0 mmPAT: Off Rel. SNR: 1.00 : fl_r

Properties

Prio recon	Off
Load images to viewer	On
Inline movie	Off
Auto store images	On
Load images to stamp segments	Off Load
images to graphic segments	Off Auto
open inline display	Off
Auto close inline display	Off
Start measurement without further preparation	Off
Wait for user to start	Off
Start measurements	Single measurement

Routine

Slice group	1
Slices	1
Dist. factor	20 %
Position	Isocenter
Orientation	Transversal
Phase enc. dir.	A >> P
AutoAlign	---
Phase oversampling	0 %
FoV read	300 mm
FoV phase	100.0 %
Slice thickness	6.0 mm
TR	118.36 ms
TE	3.37 ms
Averages	1
Concatenations	1
Filter	Distortion Corr.(2D)
Coil elements	BO1-3;SP1-4

Contrast - Common

TR	118.36 ms
TE	3.37 ms
TD	0 ms
Flip angle	40 deg
Wrap-up Magn.	None

Contrast - Dynamic

Averages	1
Averaging mode	Short term
Reconstruction	Magnitude
Measurements	1
Multiple series	Off

Resolution - Common

FoV read	300 mm
FoV phase	100.0 %
Slice thickness	6.0 mm
Base resolution	112
Radial views	132
Radial interleaves	1
Trajectory	Radial
View sharing	Off
Interpolation	Off

Resolution - iPAT

PAT mode	None

Resolution - Filter Image

Image Filter	Off
Distortion Corr.	On
Mode	2D
Unfiltered images	Off
Prescan Normalize	Off
Normalize	Off
B1 filter	Off

Resolution - Filter Rawdata

Raw filter	Off
Elliptical filter	Off
POCS	Off

Geometry - Common

Slice group	1
Slices	1
Dist. factor	20 %
Position	Isocenter
Orientation	Transversal
Phase enc. dir.	A >> P
FoV read	300 mm
FoV phase	100.0 %
Slice thickness	6.0 mm
TR	118.36 ms
Multi-slice mode	Sequential
Series	Interleaved
Concatenations	1

Geometry - AutoAlign

Slice group	1
Position	Isocenter
Orientation	Transversal
Phase enc. dir.	A >> P
AutoAlign	---
Initial Position	Isocenter
Phase	0.0 mm
Read	0.0 mm
Shift	0.0 mm
Initial Rotation	0.00 deg
Initial Orientation	Transversal

Geometry - Saturation

Wrap-up Magn.	None
Special sat.	None

Geometry - Navigator

Geometry - Tim Planning Suite

Set-n-Go Protocol	Off
Table position	H
Table position	0 mm
Inline Composing	Off

System - Miscellaneous

Positioning mode	ISO
Table position	H
Table position	0 mm
MSMA	S - C - T
Sagittal	R >> L
Coronal	A >> P

© 2025 Steffen Johansson R et al. JAMA Network Open.

System - Miscellaneous

Transversal	F >> H
Coil Combine Mode	Sum of Squares
Matrix Optimization	Off
Coil Focus	Flat
AutoAlign	---
Coil Select Mode	Default

System - Adjustments

B0 Shim mode	Cardiac
Adjust with body coil	Off
Confirm freq. adjustment	Off
Assume Dominant Fat	Off
Assume Silicone	Off
Adjustment Tolerance	Auto

System - Adjust Volume

! Position	Isocenter
! Orientation	Transversal
! Rotation	0.00 deg
! A >> P	500 mm
! R >> L	500 mm
! F >> H	6 mm
Reset	Off

System - Tx/Rx

Frequency 1H	63.672141 MHz
Correction factor	1
Gain	High
Img. Scale Cor.	1.000
Reset	Off
? Ref. amplitude 1H	0.000 V

Physio - Signal1

1st Signal/Mode	ECG/Retro
Average cycle	430 ± 159 ms
Average cycle	No Signal ms
Calculated phases	20
TR	118.36 ms
Concatenations	1
Segments	11
Arrhythmia detection	None

Physio - PACE

Resp. control	Off
Concatenations	1

Angio - Common

Flow mode	Single dir.
Encodings	1
Velocity enc.	100 cm/s
Direction	R >> L
Rephased images	On
Magnitude images	On
Magnitude sum	Off
Phase images	On

Angio - Inline

Subtract	Off
Measurements	1
StdDev	Off
Save original images	On

Angio - Cardiac

Inline Evaluation	Off
TE	3.37 ms
TR	118.36 ms
Save original images	On

Angio - MIP

MIP-Sag	Off
MIP-Cor	Off
MIP-Tra	Off
MIP-Time	Off
Save original images	On

Angio - Composing

Inline Composing	Off
Distortion Corr.	On
Mode	2D
Unfiltered images	Off

Sequence - Part 1

Introduction	Off
Dimension	2D
Asymmetric echo	Off
Flow comp.	Slice/Read
Optimization	Min. TE TR
Multi-slice mode	Sequential
Echo spacing	5.4 ms
Sequence type	Gre
Bandwidth	558 Hz/Px

Sequence - Part 2

Define	Segments
Shots per slice	12
Segments	11
RF pulse type	Normal
Gradient mode	Fast
Excitation	Slice-sel.
Flip angle mode	Constant
RF spoiling	On
Phase Enc. Rewinder	On
Cine	On

Sequence - Special

Vel Encoding	Symmetric
Radial ice program	IceProgramFire_SWIG_F(

Sequence - Assistant

Mode	Min flip angle
Min flip angle	15 deg
Allowed delay	5 s

© 2025 Steffen Johansson R et al. JAMA Network Open.

\\USER\Kliniska hjartan\Hjärtprotokollet\COVID 6m\BEAT_FQ_SWIG_MYO_IP_20
TA: 5.6 s PM: ISO Voxel size: 2.7×2.7×6.0 mmPAT: Off Rel. SNR: 1.00 : fl_r

Properties

Prio recon	Off
Load images to viewer	On
Inline movie	Off
Auto store images	On
Load images to stamp segments	Off Load
images to graphic segments	Off Auto
open inline display	Off
Auto close inline display	Off
Start measurement without further preparation	Off
Wait for user to start	Off
Start measurements	Single measurement

Routine

Slice group	1
Slices	1
Dist. factor	20 %
Position	Isocenter
Orientation	Transversal
Phase enc. dir.	A >> P
AutoAlign	---
Phase oversampling	0 %
FoV read	300 mm
FoV phase	100.0 %
Slice thickness	6.0 mm
TR	151.80 ms
TE	4.89 ms
Averages	1
Concatenations	1
Filter	Distortion Corr.(2D)
Coil elements	BO1-3;SP1-4

Contrast - Common

TR	151.80 ms
TE	4.89 ms
TD	0 ms
Flip angle	9 deg
Wrap-up Magn.	None

Contrast - Dynamic

Averages	1
Averaging mode	Short term
Reconstruction	Magnitude
Measurements	1
Multiple series	Off

Resolution - Common

FoV read	300 mm
FoV phase	100.0 %
Slice thickness	6.0 mm
Base resolution	112
Radial views	132
Radial interleaves	1
Trajectory	Radial
View sharing	Off
Interpolation	Off

Resolution - iPAT

PAT mode	None

Resolution - Filter Image

Image Filter	Off
Distortion Corr.	On
Mode	2D
Unfiltered images	Off
Prescan Normalize	Off
Normalize	Off
B1 filter	Off

Resolution - Filter Rawdata

Raw filter	Off
Elliptical filter	Off
POCS	Off

Geometry - Common

Slice group	1
Slices	1
Dist. factor	20 %
Position	Isocenter
Orientation	Transversal
Phase enc. dir.	A >> P
FoV read	300 mm
FoV phase	100.0 %
Slice thickness	6.0 mm
TR	151.80 ms
Multi-slice mode	Sequential
Series	Interleaved
Concatenations	1

Geometry - AutoAlign

Slice group	1
Position	Isocenter
Orientation	Transversal
Phase enc. dir.	A >> P
AutoAlign	---
Initial Position	Isocenter
Phase	0.0 mm
Read	0.0 mm
Shift	0.0 mm
Initial Rotation	0.00 deg
Initial Orientation	Transversal

Geometry - Saturation

Wrap-up Magn.	None
Special sat.	None

Geometry - Navigator

Geometry - Tim Planning Suite

Set-n-Go Protocol	Off
Table position	H
Table position	0 mm
Inline Composing	Off

System - Miscellaneous

Positioning mode	ISO
Table position	H
Table position	0 mm
MSMA	S - C - T
Sagittal	R >> L
Coronal	A >> P

© 2025 Steffen Johansson R et al. JAMA Network Open.

System - Miscellaneous

Transversal	F >> H
Coil Combine Mode	Sum of Squares
Matrix Optimization	Off
Coil Focus	Flat
AutoAlign	---
Coil Select Mode	Default

System - Adjustments

B0 Shim mode	Cardiac
Adjust with body coil	Off
Confirm freq. adjustment	Off
Assume Dominant Fat	Off
Assume Silicone	Off
Adjustment Tolerance	Auto

System - Adjust Volume

! Position	Isocenter
! Orientation	Transversal
! Rotation	0.00 deg
! A >> P	500 mm
! R >> L	500 mm
! F >> H	6 mm
Reset	Off

System - Tx/Rx

Frequency 1H	63.672141 MHz
Correction factor	1
Gain	High
Img. Scale Cor.	1.000
Reset	Off
? Ref. amplitude 1H	0.000 V

Physio - Signal1

1st Signal/Mode	ECG/Retro
Average cycle	430 ± 159 ms
Average cycle	No Signal ms
Calculated phases	20
TR	151.80 ms
Concatenations	1
Segments	11
Arrhythmia detection	None

Physio - PACE

Resp. control	Off
Concatenations	1

Angio - Common

Flow mode	Single dir.
Encodings	1
Velocity enc.	20 cm/s
Direction	R >> L
Rephased images	On
Magnitude images	On
Magnitude sum	Off
Phase images	On

Angio - Inline

Subtract	Off
Measurements	1
StdDev	Off
Save original images	On

Angio - Cardiac

Inline Evaluation	Off
TE	4.89 ms
TR	151.80 ms
Save original images	On

Angio - MIP

MIP-Sag	Off
MIP-Cor	Off
MIP-Tra	Off
MIP-Time	Off
Save original images	On

Angio - Composing

Inline Composing	Off
Distortion Corr.	On
Mode	2D
Unfiltered images	Off

Sequence - Part 1

Introduction	Off
Dimension	2D
Asymmetric echo	Off
Flow comp.	Slice/Read
Optimization	Min. TE TR
Multi-slice mode	Sequential
Echo spacing	6.9 ms
Sequence type	Gre
Bandwidth	558 Hz/Px

Sequence - Part 2

Define	Segments
Shots per slice	12
Segments	11
RF pulse type	Normal
Gradient mode	Fast
Excitation	Slice-sel.
Flip angle mode	Constant
RF spoiling	On
Phase Enc. Rewinder	On
Cine	On

Sequence - Special

Vel Encoding	Symmetric
Radial ice program	IceProgramFire_SWIG_F(

Sequence - Assistant

Mode	Min flip angle
Min flip angle	15 deg
Allowed delay	5 s

© 2025 Steffen Johansson R et al. JAMA Network Open.

\\USER\Kliniska hjartan\Hjärtprotokollet\COVID 6m\MOLLI_TI_scout
TA: 9.5 s PM: FIX Voxel size: 1.9×1.9×8.0 mmPAT: 2 Rel. SNR: 1.00 : wip_tfi

Properties

Prio recon	Off
Load images to viewer	On
Inline movie	Off
Auto store images	On
Load images to stamp segments	Off Load
images to graphic segments	On Auto
open inline display	Off
Auto close inline display	Off
Start measurement without further preparation	Off
Wait for user to start	On
Start measurements	Single measurement

Routine

Slice group	1
Slices	1
Dist. factor	20 %
Position	Isocenter
Orientation	Transversal
Phase enc. dir.	A >> P
AutoAlign	---
Phase oversampling	0 %
FoV read	360 mm
FoV phase	75.0 %
Slice thickness	8.0 mm
TR	336.56 ms
TE	1.01 ms
Averages	1
Concatenations	1
Filter	Distortion Corr.(2D)
Coil elements	BO1-3;SP2,3

Contrast - Common

TR	336.56 ms
TE	1.01 ms
Magn. preparation	Non-sel. IR T1map
TI	260 ms
Flip angle	35 deg
Fat suppr.	None
Wrap-up Magn.	None

Contrast - Dynamic

Averages	1
Averaging mode	Short term
Reconstruction	Magnitude
Measurements	1
Multiple series	Off

Resolution - Common

FoV read	360 mm
FoV phase	75.0 %
Slice thickness	8.0 mm
Base resolution	192
Phase resolution	83 %
Phase partial Fourier	7/8
Trajectory	Cartesian
Interpolation	Off

Resolution - iPAT

PAT mode	GRAPPA

Resolution - iPAT

Accel. factor PE	2
Ref. lines PE	36
Reference scan mode	GRE/separate

Resolution - Filter Image

Image Filter	Off
Distortion Corr.	On
Mode	2D
Unfiltered images	Off
Prescan Normalize	Off
Normalize	Off
B1 filter	Off

Resolution - Filter Rawdata

Raw filter	Off
Elliptical filter	Off
POCS	Off

Geometry - Common

Slice group	1
Slices	1
Dist. factor	20 %
Position	Isocenter
Orientation	Transversal
Phase enc. dir.	A >> P
FoV read	360 mm
FoV phase	75.0 %
Slice thickness	8.0 mm
TR	336.56 ms
Multi-slice mode	Sequential
Series	Interl. in B.-h.
Concatenations	1

Geometry - AutoAlign

Slice group	1
Position	Isocenter
Orientation	Transversal
Phase enc. dir.	A >> P
AutoAlign	---
Initial Position	Isocenter
Phase	0.0 mm
Read	0.0 mm
Shift	0.0 mm
Initial Rotation	0.00 deg
Initial Orientation	Transversal

Geometry - Saturation

Fat suppr.	None
Wrap-up Magn.	None
Special sat.	None

Geometry - Navigator

Geometry - Tim Planning Suite

Set-n-Go Protocol	Off
Table position	H
Table position	0 mm
Inline Composing	Off

© 2025 Steffen Johansson R et al. JAMA Network Open.

System - Miscellaneous

Positioning mode	FIX
Table position	H
Table position	0 mm
MSMA	S - C - T
Sagittal	R >> L
Coronal	A >> P
Transversal	F >> H
Coil Combine Mode	Adaptive Combine
Save uncombined	Off
Matrix Optimization	Off
Coil Focus	Flat
AutoAlign	---
Coil Select Mode	Default

System - Adjustments

B0 Shim mode	Cardiac
Adjust with body coil	On
Confirm freq. adjustment	Off
Assume Dominant Fat	Off
Assume Silicone	Off
Adjustment Tolerance	Auto

System - Adjust Volume

Position	Isocenter
Orientation	Transversal
Rotation	0.00 deg
A >> P	270 mm
R >> L	360 mm
F >> H	8 mm
Reset	Off

System - Tx/Rx

Frequency 1H	63.672141 MHz
Correction factor	1
Gain	High
Img. Scale Cor.	1.000
Reset	Off
? Ref. amplitude 1H	0.000 V

Physio - Signal1

1st Signal/Mode	ECG/Trigger
Average cycle	430 ± 159 ms
Average cycle	No Signal ms
Captured cycle	430 ± 159 ms
Acquisition window	779 ms
Trigger pulse	1
Trigger delay	442 ms
TR	336.56 ms
Concatenations	1
Segments	60
Phases	1
Adaptive Triggering	Off

Physio - Cardiac

Tagging	None
Magn. preparation	Non-sel. IR T1map
TI	260 ms
Fat suppr.	None
Dark blood	Off
FoV read	360 mm
FoV phase	75.0 %
Phase resolution	83 %
Cine	Off
Trajectory	Cartesian

Physio - Cardiac

Dummy heartbeats	0
Motion Correction	Standard

Physio - PACE

Resp. control	Breath-hold
Concatenations	1

Inline - Common

Subtract	Off
Measurements	1
StdDev	Off
Motion Correction	Standard
Save original images	On

Inline - Cardiac

Inline Evaluation	T1 map
Magn. preparation	Non-sel. IR T1map
Num. of preps	3
Sampling duration 1	4 beats
Sampling duration 2	3 beats
Sampling duration 3	2 beats
Contrasts	1
TE	1.01 ms
TR	336.56 ms
Recovery duration 1	1 beats
Recovery duration 2	1 beats
Recovery duration 3	0 beats
Motion Correction	Standard
Save original images	On

Inline - MIP

MIP-Sag	Off
MIP-Cor	Off
MIP-Tra	Off
MIP-Time	Off
Save original images	On

Inline - Composing

Inline Composing	Off
Distortion Corr.	On
Mode	2D
Unfiltered images	Off

Sequence - Part 1

Introduction	Off
Dimension	2D
Reordering	Linear
Asymmetric echo	Weak
Contrasts	1
Optimization	Min. TE TR
Multi-slice mode	Sequential
Echo spacing	2.4 ms
Sequence type	Trufi
Bandwidth	1085 Hz/Px

Sequence - Part 2

Define	Shots
Shots per slice	1
Segments	60
Trufi delta freq.	0 Hz
RF pulse type	Fast
Gradient mode	Fast
Excitation	Slice-sel.

© 2025 Steffen Johansson R et al. JAMA Network Open.

Sequence - Part 2

Flip angle mode	Constant
Cine	Off

Sequence - Special

Partition coeff map	Off
ECV map	Off
Synth ECV map	Off
TI scout mode	On
Error map	Off
Synth PSIR	Off
Periods in seconds	Off
16 bit images	Off
T1 sampling scheme	Post Gd

Sequence - Assistant

Mode	Off
Allowed delay	0 s

© 2025 Steffen Johansson R et al. JAMA Network Open.

\\USER\Kliniska hjartan\Hjärtprotokollet\COVID 6m\PSIR SA MOCO_NoScout
TA: 2:33 PM: FIX Voxel size: 1.4×1.4×8.0 mmPAT: 2 Rel. SNR: 1.00 : tfi

Properties

Prio recon	Off
Load images to viewer	Off
Inline movie	Off
Auto store images	On
Load images to stamp segments	Off Load
images to graphic segments	On Auto
open inline display	Off
Auto close inline display	Off
Start measurement without further preparation	Off
Wait for user to start	Off
Start measurements	Single measurement

Routine

Slice group	1
Slices	11
Dist. factor	20 %
Position	Isocenter
Orientation	Transversal
Phase enc. dir.	A >> P
AutoAlign	---
Phase oversampling	0 %
FoV read	360 mm
FoV phase	75.0 %
Slice thickness	8.0 mm
TR	904.00 ms
TE	1.19 ms
Averages	8
Concatenations	11
Filter	Distortion Corr.(2D)
Coil elements	BO1-3;SP1-3

Contrast - Common

TR	904.00 ms
TE	1.19 ms
TD	0 ms
Magn. preparation	Non-sel. IR
TI	300 ms
Flip angle	50 deg
Fat suppr.	None
Wrap-up Magn.	None

Contrast - Dynamic

Averages	8
Averaging mode	Short term
Reconstruction	Magnitude/Real
Measurements	1
Multiple series	Off

Resolution - Common

FoV read	360 mm
FoV phase	75.0 %
Slice thickness	8.0 mm
Base resolution	256
Phase resolution	75 %
Phase partial Fourier	Off
Trajectory	Cartesian
Interpolation	Off

Resolution - iPAT

PAT mode	GRAPPA
Accel. factor PE	2
Ref. lines PE	32
Reference scan mode	GRE/separate

Resolution - Filter Image

Image Filter	Off
Distortion Corr.	On
Mode	2D
Unfiltered images	Off
Prescan Normalize	Off
Normalize	Off
B1 filter	Off

Resolution - Filter Rawdata

Raw filter	Off
Elliptical filter	Off
POCS	Off

Geometry - Common

Slice group	1
Slices	11
Dist. factor	20 %
Position	Isocenter
Orientation	Transversal
Phase enc. dir.	A >> P
FoV read	360 mm
FoV phase	75.0 %
Slice thickness	8.0 mm
TR	904.00 ms
Multi-slice mode	Sequential
Series	Interleaved
Concatenations	11

Geometry - AutoAlign

Slice group	1
Position	Isocenter
Orientation	Transversal
Phase enc. dir.	A >> P
AutoAlign	---
Initial Position	Isocenter
Phase	0.0 mm
Read	0.0 mm
Shift	0.0 mm
Initial Rotation	0.00 deg
Initial Orientation	Transversal

Geometry - Saturation

Fat suppr.	None
Wrap-up Magn.	None
Special sat.	None

Geometry - Navigator

Geometry - Tim Planning Suite

Set-n-Go Protocol	Off
Table position	H
Table position	0 mm
Inline Composing	Off

© 2025 Steffen Johansson R et al. JAMA Network Open.

System - Miscellaneous

Positioning mode	FIX
Table position	H
Table position	0 mm
MSMA	S - C - T
Sagittal	R >> L
Coronal	A >> P
Transversal	F >> H
Coil Combine Mode	Adaptive Combine
Save uncombined	Off
Matrix Optimization	Off
Coil Focus	Flat
AutoAlign	---
Coil Select Mode	Default

System - Adjustments

B0 Shim mode	Cardiac
Adjust with body coil	On
Confirm freq. adjustment	Off
Assume Dominant Fat	Off
Assume Silicone	Off
Adjustment Tolerance	Auto

System - Adjust Volume

Position	Isocenter
Orientation	Transversal
Rotation	0.00 deg
A >> P	270 mm
R >> L	360 mm
F >> H	104 mm
Reset	Off

System - Tx/Rx

Frequency 1H	63.672141 MHz
Correction factor	1
Gain	High
Img. Scale Cor.	1.000
Reset	Off
? Ref. amplitude 1H	0.000 V

Physio - Signal1

1st Signal/Mode	ECG/Trigger
Average cycle	430 ± 159 ms
Average cycle	No Signal ms
Captured cycle	430 ± 159 ms
Acquisition window	930 ms
Trigger pulse	2
Trigger delay	0 ms
TR	904.00 ms
Concatenations	11
Segments	72
Phases	1
Adaptive Triggering	Off

Physio - Cardiac

Tagging	None
Magn. preparation	Non-sel. IR
TI	300 ms
Fat suppr.	None
Dark blood	Off
FoV read	360 mm
FoV phase	75.0 %
Phase resolution	75 %
Cine	Off
Trajectory	Cartesian

Physio - Cardiac

Dummy heartbeats	1

Physio - PACE

Resp. control	Off
Concatenations	11

Inline - Common

Subtract	Off
Measurements	1
StdDev	Off
Save original images	On

Inline - Cardiac

Inline Evaluation	Off
Magn. preparation	Non-sel. IR
Contrasts	1
TE	1.19 ms
TR	904.00 ms
Save original images	On

Inline - MIP

MIP-Sag	Off
MIP-Cor	Off
MIP-Tra	Off
MIP-Time	Off
Save original images	On

Inline - Composing

Inline Composing	Off
Distortion Corr.	On
Mode	2D
Unfiltered images	Off

Sequence - Part 1

Introduction	Off
Dimension	2D
Reordering	Linear
Asymmetric echo	Weak
Contrasts	1
Optimization	Min. TE
Multi-slice mode	Sequential
Echo spacing	2.8 ms
Sequence type	Trufi
Bandwidth	977 Hz/Px

Sequence - Part 2

Define	Shots
Shots per slice	1
Segments	72
Trufi delta freq.	0 Hz
RF pulse type	Fast
Gradient mode	Fast
Excitation	Slice-sel.
Flip angle mode	Constant
Cine	Off

Sequence - Special

Gadgetron IPR	PSIR

Sequence - Assistant

Mode	Min flip angle
Min flip angle	30 deg
Allowed delay	5 s

© 2025 Steffen Johansson R et al. JAMA Network Open.

\\USER\Kliniska hjartan\Hjärtprotokollet\COVID 6m\PSIR 4ch MOCO_NoScout
TA: 0:15 PM: FIX Voxel size: 1.4×1.4×8.0 mmPAT: 2 Rel. SNR: 1.00 : tfi

Properties

Prio recon	Off
Load images to viewer	Off
Inline movie	Off
Auto store images	On
Load images to stamp segments	Off Load
images to graphic segments	On Auto
open inline display	Off
Auto close inline display	Off
Start measurement without further preparation	Off
Wait for user to start	Off
Start measurements	Single measurement

Routine

Slice group	1
Slices	1
Dist. factor	20 %
Position	Isocenter
Orientation	Transversal
Phase enc. dir.	A >> P
AutoAlign	---
Phase oversampling	0 %
FoV read	360 mm
FoV phase	75.0 %
Slice thickness	8.0 mm
TR	904.00 ms
TE	1.19 ms
Averages	8
Concatenations	1
Filter	Distortion Corr.(2D)
Coil elements	BO1-3;SP2,3

Contrast - Common

TR	904.00 ms
TE	1.19 ms
Magn. preparation	Non-sel. IR
TI	300 ms
Flip angle	50 deg
Fat suppr.	None
Wrap-up Magn.	None

Contrast - Dynamic

Averages	8
Averaging mode	Short term
Reconstruction	Magnitude/Real
Measurements	1
Multiple series	Off

Resolution - Common

FoV read	360 mm
FoV phase	75.0 %
Slice thickness	8.0 mm
Base resolution	256
Phase resolution	75 %
Phase partial Fourier	Off
Trajectory	Cartesian
Interpolation	Off

Resolution - iPAT

PAT mode	GRAPPA

© 2025 Steffen Johansson R et al. JAMA Network Open.

Resolution - iPAT

Accel. factor PE	2
Ref. lines PE	32
Reference scan mode	GRE/separate

Resolution - Filter Image

Image Filter	Off
Distortion Corr.	On
Mode	2D
Unfiltered images	Off
Prescan Normalize	Off
Normalize	Off
B1 filter	Off

Resolution - Filter Rawdata

Raw filter	Off
Elliptical filter	Off
POCS	Off

Geometry - Common

Slice group	1
Slices	1
Dist. factor	20 %
Position	Isocenter
Orientation	Transversal
Phase enc. dir.	A >> P
FoV read	360 mm
FoV phase	75.0 %
Slice thickness	8.0 mm
TR	904.00 ms
Multi-slice mode	Sequential
Series	Interleaved
Concatenations	1

Geometry - AutoAlign

Slice group	1
Position	Isocenter
Orientation	Transversal
Phase enc. dir.	A >> P
AutoAlign	---
Initial Position	Isocenter
Phase	0.0 mm
Read	0.0 mm
Shift	0.0 mm
Initial Rotation	0.00 deg
Initial Orientation	Transversal

Geometry - Saturation

Fat suppr.	None
Wrap-up Magn.	None
Special sat.	None

Geometry - Navigator

Geometry - Tim Planning Suite

Set-n-Go Protocol	Off
Table position	H
Table position	0 mm
Inline Composing	Off

System - Miscellaneous

Positioning mode	FIX
Table position	H
Table position	0 mm
MSMA	S - C - T
Sagittal	R >> L
Coronal	A >> P
Transversal	F >> H
Coil Combine Mode	Adaptive Combine
Save uncombined	Off
Matrix Optimization	Off
Coil Focus	Flat
AutoAlign	---
Coil Select Mode	Default

System - Adjustments

B0 Shim mode	Cardiac
Adjust with body coil	On
Confirm freq. adjustment	Off
Assume Dominant Fat	Off
Assume Silicone	Off
Adjustment Tolerance	Auto

System - Adjust Volume

Position	Isocenter
Orientation	Transversal
Rotation	0.00 deg
A >> P	270 mm
R >> L	360 mm
F >> H	8 mm
Reset	Off

System - Tx/Rx

Frequency 1H	63.672141 MHz
Correction factor	1
Gain	High
Img. Scale Cor.	1.000
Reset	Off
? Ref. amplitude 1H	0.000 V

Physio - Signal1

1st Signal/Mode	ECG/Trigger
Average cycle	430 ± 159 ms
Average cycle	No Signal ms
Captured cycle	430 ± 159 ms
Acquisition window	930 ms
Trigger pulse	2
Trigger delay	0 ms
TR	904.00 ms
Concatenations	1
Segments	72
Phases	1
Adaptive Triggering	Off

Physio - Cardiac

Tagging	None
Magn. preparation	Non-sel. IR
TI	300 ms
Fat suppr.	None
Dark blood	Off
FoV read	360 mm
FoV phase	75.0 %
Phase resolution	75 %
Cine	Off
Trajectory	Cartesian

Physio - Cardiac

Dummy heartbeats	1

Physio - PACE

Resp. control	Off
Concatenations	1

Inline - Common

Subtract	Off
Measurements	1
StdDev	Off
Save original images	On

Inline - Cardiac

Inline Evaluation	Off
Magn. preparation	Non-sel. IR
Contrasts	1
TE	1.19 ms
TR	904.00 ms
Save original images	On

Inline - MIP

MIP-Sag	Off
MIP-Cor	Off
MIP-Tra	Off
MIP-Time	Off
Save original images	On

Inline - Composing

Inline Composing	Off
Distortion Corr.	On
Mode	2D
Unfiltered images	Off

Sequence - Part 1

Introduction	Off
Dimension	2D
Reordering	Linear
Asymmetric echo	Weak
Contrasts	1
Optimization	Min. TE
Multi-slice mode	Sequential
Echo spacing	2.8 ms
Sequence type	Trufi
Bandwidth	977 Hz/Px

Sequence - Part 2

Define	Shots
Shots per slice	1
Segments	72
Trufi delta freq.	0 Hz
RF pulse type	Fast
Gradient mode	Fast
Excitation	Slice-sel.
Flip angle mode	Constant
Cine	Off

Sequence - Special

Gadgetron IPR	PSIR

Sequence - Assistant

Mode	Min flip angle
Min flip angle	30 deg
Allowed delay	0 s

© 2025 Steffen Johansson R et al. JAMA Network Open.

\\USER\Kliniska hjartan\Hjärtprotokollet\COVID 6m\PSIR 3ch MOCO_NoScout
TA: 0:15 PM: FIX Voxel size: 1.4×1.4×8.0 mmPAT: 2 Rel. SNR: 1.00 : tfi

Properties

Prio recon	Off
Load images to viewer	Off
Inline movie	Off
Auto store images	On
Load images to stamp segments	Off Load
images to graphic segments	On Auto
open inline display	Off
Auto close inline display	Off
Start measurement without further preparation	Off
Wait for user to start	Off
Start measurements	Single measurement

Routine

Slice group	1
Slices	1
Dist. factor	20 %
Position	Isocenter
Orientation	Transversal
Phase enc. dir.	A >> P
AutoAlign	---
Phase oversampling	0 %
FoV read	360 mm
FoV phase	75.0 %
Slice thickness	8.0 mm
TR	904.00 ms
TE	1.19 ms
Averages	8
Concatenations	1
Filter	Distortion Corr.(2D)
Coil elements	BO1-3;SP2,3

Contrast - Common

TR	904.00 ms
TE	1.19 ms
Magn. preparation	Non-sel. IR
TI	300 ms
Flip angle	50 deg
Fat suppr.	None
Wrap-up Magn.	None

Contrast - Dynamic

Averages	8
Averaging mode	Short term
Reconstruction	Magnitude/Real
Measurements	1
Multiple series	Off

Resolution - Common

FoV read	360 mm
FoV phase	75.0 %
Slice thickness	8.0 mm
Base resolution	256
Phase resolution	75 %
Phase partial Fourier	Off
Trajectory	Cartesian
Interpolation	Off

Resolution - iPAT

PAT mode	GRAPPA

Resolution - iPAT

Accel. factor PE	2
Ref. lines PE	32
Reference scan mode	GRE/separate

Resolution - Filter Image

Image Filter	Off
Distortion Corr.	On
Mode	2D
Unfiltered images	Off
Prescan Normalize	Off
Normalize	Off
B1 filter	Off

Resolution - Filter Rawdata

Raw filter	Off
Elliptical filter	Off
POCS	Off

Geometry - Common

Slice group	1
Slices	1
Dist. factor	20 %
Position	Isocenter
Orientation	Transversal
Phase enc. dir.	A >> P
FoV read	360 mm
FoV phase	75.0 %
Slice thickness	8.0 mm
TR	904.00 ms
Multi-slice mode	Sequential
Series	Interleaved
Concatenations	1

Geometry - AutoAlign

Slice group	1
Position	Isocenter
Orientation	Transversal
Phase enc. dir.	A >> P
AutoAlign	---
Initial Position	Isocenter
Phase	0.0 mm
Read	0.0 mm
Shift	0.0 mm
Initial Rotation	0.00 deg
Initial Orientation	Transversal

Geometry - Saturation

Fat suppr.	None
Wrap-up Magn.	None
Special sat.	None

Geometry - Navigator

Geometry - Tim Planning Suite

Set-n-Go Protocol	Off
Table position	H
Table position	0 mm
Inline Composing	Off

© 2025 Steffen Johansson R et al. JAMA Network Open.

System - Miscellaneous

Positioning mode	FIX
Table position	H
Table position	0 mm
MSMA	S - C - T
Sagittal	R >> L
Coronal	A >> P
Transversal	F >> H
Coil Combine Mode	Adaptive Combine
Save uncombined	Off
Matrix Optimization	Off
Coil Focus	Flat
AutoAlign	---
Coil Select Mode	Default

System - Adjustments

B0 Shim mode	Cardiac
Adjust with body coil	On
Confirm freq. adjustment	Off
Assume Dominant Fat	Off
Assume Silicone	Off
Adjustment Tolerance	Auto

System - Adjust Volume

Position	Isocenter
Orientation	Transversal
Rotation	0.00 deg
A >> P	270 mm
R >> L	360 mm
F >> H	8 mm
Reset	Off

System - Tx/Rx

Frequency 1H	63.672141 MHz
Correction factor	1
Gain	High
Img. Scale Cor.	1.000
Reset	Off
? Ref. amplitude 1H	0.000 V

Physio - Signal1

1st Signal/Mode	ECG/Trigger
Average cycle	430 ± 159 ms
Average cycle	No Signal ms
Captured cycle	430 ± 159 ms
Acquisition window	930 ms
Trigger pulse	2
Trigger delay	0 ms
TR	904.00 ms
Concatenations	1
Segments	72
Phases	1
Adaptive Triggering	Off

Physio - Cardiac

Tagging	None
Magn. preparation	Non-sel. IR
TI	300 ms
Fat suppr.	None
Dark blood	Off
FoV read	360 mm
FoV phase	75.0 %
Phase resolution	75 %
Cine	Off
Trajectory	Cartesian

Physio - Cardiac

Dummy heartbeats	1

Physio - PACE

Resp. control	Off
Concatenations	1

Inline - Common

Subtract	Off
Measurements	1
StdDev	Off
Save original images	On

Inline - Cardiac

Inline Evaluation	Off
Magn. preparation	Non-sel. IR
Contrasts	1
TE	1.19 ms
TR	904.00 ms
Save original images	On

Inline - MIP

MIP-Sag	Off
MIP-Cor	Off
MIP-Tra	Off
MIP-Time	Off
Save original images	On

Inline - Composing

Inline Composing	Off
Distortion Corr.	On
Mode	2D
Unfiltered images	Off

Sequence - Part 1

Introduction	Off
Dimension	2D
Reordering	Linear
Asymmetric echo	Weak
Contrasts	1
Optimization	Min. TE
Multi-slice mode	Sequential
Echo spacing	2.8 ms
Sequence type	Trufi
Bandwidth	977 Hz/Px

Sequence - Part 2

Define	Shots
Shots per slice	1
Segments	72
Trufi delta freq.	0 Hz
RF pulse type	Fast
Gradient mode	Fast
Excitation	Slice-sel.
Flip angle mode	Constant
Cine	Off

Sequence - Special

Gadgetron IPR	PSIR

Sequence - Assistant

Mode	Min flip angle
Min flip angle	30 deg
Allowed delay	0 s

© 2025 Steffen Johansson R et al. JAMA Network Open.

\\USER\Kliniska hjartan\Hjärtprotokollet\COVID 6m\PSIR 2ch MOCO_NoScout
TA: 0:15 PM: FIX Voxel size: 1.4×1.4×8.0 mmPAT: 2 Rel. SNR: 1.00 : tfi

Properties

Prio recon	Off
Load images to viewer	Off
Inline movie	Off
Auto store images	On
Load images to stamp segments	Off Load
images to graphic segments	On Auto
open inline display	Off
Auto close inline display	Off
Start measurement without further preparation	Off
Wait for user to start	Off
Start measurements	Single measurement

Routine

Slice group	1
Slices	1
Dist. factor	20 %
Position	Isocenter
Orientation	Transversal
Phase enc. dir.	A >> P
AutoAlign	---
Phase oversampling	0 %
FoV read	360 mm
FoV phase	75.0 %
Slice thickness	8.0 mm
TR	904.00 ms
TE	1.19 ms
Averages	8
Concatenations	1
Filter	Distortion Corr.(2D)
Coil elements	BO1-3;SP2,3

Contrast - Common

TR	904.00 ms
TE	1.19 ms
Magn. preparation	Non-sel. IR
TI	300 ms
Flip angle	50 deg
Fat suppr.	None
Wrap-up Magn.	None

Contrast - Dynamic

Averages	8
Averaging mode	Short term
Reconstruction	Magnitude/Real
Measurements	1
Multiple series	Off

Resolution - Common

FoV read	360 mm
FoV phase	75.0 %
Slice thickness	8.0 mm
Base resolution	256
Phase resolution	75 %
Phase partial Fourier	Off
Trajectory	Cartesian
Interpolation	Off

Resolution - iPAT

PAT mode	GRAPPA

Resolution - iPAT

Accel. factor PE	2
Ref. lines PE	32
Reference scan mode	GRE/separate

Resolution - Filter Image

Image Filter	Off
Distortion Corr.	On
Mode	2D
Unfiltered images	Off
Prescan Normalize	Off
Normalize	Off
B1 filter	Off

Resolution - Filter Rawdata

Raw filter	Off
Elliptical filter	Off
POCS	Off

Geometry - Common

Slice group	1
Slices	1
Dist. factor	20 %
Position	Isocenter
Orientation	Transversal
Phase enc. dir.	A >> P
FoV read	360 mm
FoV phase	75.0 %
Slice thickness	8.0 mm
TR	904.00 ms
Multi-slice mode	Sequential
Series	Interleaved
Concatenations	1

Geometry - AutoAlign

Slice group	1
Position	Isocenter
Orientation	Transversal
Phase enc. dir.	A >> P
AutoAlign	---
Initial Position	Isocenter
Phase	0.0 mm
Read	0.0 mm
Shift	0.0 mm
Initial Rotation	0.00 deg
Initial Orientation	Transversal

Geometry - Saturation

Fat suppr.	None
Wrap-up Magn.	None
Special sat.	None

Geometry - Navigator

Geometry - Tim Planning Suite

Set-n-Go Protocol	Off
Table position	H
Table position	0 mm
Inline Composing	Off

© 2025 Steffen Johansson R et al. JAMA Network Open.

System - Miscellaneous

Positioning mode	FIX
Table position	H
Table position	0 mm
MSMA	S - C - T
Sagittal	R >> L
Coronal	A >> P
Transversal	F >> H
Coil Combine Mode	Adaptive Combine
Save uncombined	Off
Matrix Optimization	Off
Coil Focus	Flat
AutoAlign	---
Coil Select Mode	Default

System - Adjustments

B0 Shim mode	Cardiac
Adjust with body coil	On
Confirm freq. adjustment	Off
Assume Dominant Fat	Off
Assume Silicone	Off
Adjustment Tolerance	Auto

System - Adjust Volume

Position	Isocenter
Orientation	Transversal
Rotation	0.00 deg
A >> P	270 mm
R >> L	360 mm
F >> H	8 mm
Reset	Off

System - Tx/Rx

Frequency 1H	63.672141 MHz
Correction factor	1
Gain	High
Img. Scale Cor.	1.000
Reset	Off
? Ref. amplitude 1H	0.000 V

Physio - Signal1

1st Signal/Mode	ECG/Trigger
Average cycle	430 ± 159 ms
Average cycle	No Signal ms
Captured cycle	430 ± 159 ms
Acquisition window	930 ms
Trigger pulse	2
Trigger delay	0 ms
TR	904.00 ms
Concatenations	1
Segments	72
Phases	1
Adaptive Triggering	Off

Physio - Cardiac

Tagging	None
Magn. preparation	Non-sel. IR
TI	300 ms
Fat suppr.	None
Dark blood	Off
FoV read	360 mm
FoV phase	75.0 %
Phase resolution	75 %
Cine	Off
Trajectory	Cartesian

Physio - Cardiac

Dummy heartbeats	1

Physio - PACE

Resp. control	Off
Concatenations	1

Inline - Common

Subtract	Off
Measurements	1
StdDev	Off
Save original images	On

Inline - Cardiac

Inline Evaluation	Off
Magn. preparation	Non-sel. IR
Contrasts	1
TE	1.19 ms
TR	904.00 ms
Save original images	On

Inline - MIP

MIP-Sag	Off
MIP-Cor	Off
MIP-Tra	Off
MIP-Time	Off
Save original images	On

Inline - Composing

Inline Composing	Off
Distortion Corr.	On
Mode	2D
Unfiltered images	Off

Sequence - Part 1

Introduction	Off
Dimension	2D
Reordering	Linear
Asymmetric echo	Weak
Contrasts	1
Optimization	Min. TE
Multi-slice mode	Sequential
Echo spacing	2.8 ms
Sequence type	Trufi
Bandwidth	977 Hz/Px

Sequence - Part 2

Define	Shots
Shots per slice	1
Segments	72
Trufi delta freq.	0 Hz
RF pulse type	Fast
Gradient mode	Fast
Excitation	Slice-sel.
Flip angle mode	Constant
Cine	Off

Sequence - Special

Gadgetron IPR	PSIR

Sequence - Assistant

Mode	Min flip angle
Min flip angle	30 deg
Allowed delay	0 s

© 2025 Steffen Johansson R et al. JAMA Network Open.

\\USER\Kliniska hjartan\Hjärtprotokollet\COVID 6m\post_MOLLI_SA NoScout
TA: 0:52 PM: FIX Voxel size: 1.4×1.4×8.0 mmPAT: 2 Rel. SNR: 1.00 : wip_tfi

Properties

Prio recon	Off
Load images to viewer	On
Inline movie	Off
Auto store images	On
Load images to stamp segments	Off
Load images to graphic segments	On
Auto open inline display	Off
Auto close inline display	Off
Start measurement without further preparation	Off
Wait for user to start	Off
Start measurements	Single measurement

Routine

Slice group	1
Slices	5
Dist. factor	80 %
Position	Isocenter
Orientation	Transversal
Phase enc. dir.	A >> P
AutoAlign	---
Phase oversampling	0 %
FoV read	360 mm
FoV phase	75.0 %
Slice thickness	8.0 mm
TR	293.56 ms
TE	1.12 ms
Averages	1
Concatenations	5
Filter	Distortion Corr.(2D)
Coil elements	BO1-3;SP2,3

Contrast - Common

TR	293.56 ms
TE	1.12 ms
Magn. preparation	Non-sel. IR T1map
TI	193 ms
Flip angle	35 deg
Fat suppr.	Fat sat.
Wrap-up Magn.	None

Contrast - Dynamic

Averages	1
Averaging mode	Short term
Reconstruction	Magnitude
Measurements	1
Multiple series	Off

Resolution - Common

FoV read	360 mm
FoV phase	75.0 %
Slice thickness	8.0 mm
Base resolution	256
Phase resolution	75 %
Phase partial Fourier	7/8
Trajectory	Cartesian
Interpolation	Off

Resolution - iPAT

PAT mode	GRAPPA
Accel. factor PE	2
Ref. lines PE	36
Reference scan mode	GRE/separate

Resolution - Filter Image

Image Filter	Off
Distortion Corr.	On
Mode	2D
Unfiltered images	Off
Prescan Normalize	Off
Normalize	Off
B1 filter	Off

Resolution - Filter Rawdata

Raw filter	Off
Elliptical filter	Off
POCS	Off

Geometry - Common

Slice group	1
Slices	5
Dist. factor	80 %
Position	Isocenter
Orientation	Transversal
Phase enc. dir.	A >> P
FoV read	360 mm
FoV phase	75.0 %
Slice thickness	8.0 mm
TR	293.56 ms
Multi-slice mode	Sequential
Series	Interl. in B.-h.
Concatenations	5

Geometry - AutoAlign

Slice group	1
Position	Isocenter
Orientation	Transversal
Phase enc. dir.	A >> P
AutoAlign	---
Initial Position	Isocenter
Phase	0.0 mm
Read	0.0 mm
Shift	0.0 mm
Initial Rotation	0.00 deg
Initial Orientation	Transversal

Geometry - Saturation

Fat suppr.	Fat sat.
Wrap-up Magn.	None
Special sat.	None

Geometry - Navigator

Geometry - Tim Planning Suite

Set-n-Go Protocol	Off
Table position	H
Table position	0 mm
Inline Composing	Off

© 2025 Steffen Johansson R et al. JAMA Network Open.

System - Miscellaneous

Positioning mode	FIX
Table position	H
Table position	0 mm
MSMA	S - C - T
Sagittal	R >> L
Coronal	A >> P
Transversal	F >> H
Coil Combine Mode	Adaptive Combine
Save uncombined	Off
Matrix Optimization	Off
Coil Focus	Flat
AutoAlign	---
Coil Select Mode	Off - All

System - Adjustments

B0 Shim mode	Cardiac
Adjust with body coil	On
Confirm freq. adjustment	Off
Assume Dominant Fat	Off
Assume Silicone	Off
Adjustment Tolerance	Auto

System - Adjust Volume

Position	Isocenter
Orientation	Transversal
Rotation	0.00 deg
A >> P	270 mm
R >> L	360 mm
F >> H	66 mm
Reset	Off

System - Tx/Rx

Frequency 1H	63.672141 MHz
Correction factor	1
Gain	High
Img. Scale Cor.	1.000
Reset	Off
? Ref. amplitude 1H	0.000 V

Physio - Signal1

1st Signal/Mode	ECG/Trigger
Average cycle	430 ± 159 ms
Average cycle	No Signal ms
Captured cycle	430 ± 159 ms
Acquisition window	712 ms
Trigger pulse	1
Trigger delay	418 ms
TR	293.56 ms
Concatenations	5
Segments	72
Phases	1
Adaptive Triggering	Off

Physio - Cardiac

Tagging	None
Magn. preparation	Non-sel. IR T1map
TI	193 ms
Fat suppr.	Fat sat.
Dark blood	Off
FoV read	360 mm
FoV phase	75.0 %
Phase resolution	75 %
Cine	Off
Trajectory	Cartesian

Physio - Cardiac

Dummy heartbeats	0
Motion Correction	Standard

Physio - PACE

Resp. control	Breath-hold
Concatenations	5

Inline - Common

Subtract	Off
Measurements	1
StdDev	Off
Motion Correction	Standard
Save original images	On

Inline - Cardiac

Inline Evaluation	T1 map
Magn. preparation	Non-sel. IR T1map
Num. of preps	2
Sampling duration 1	5 sec.
Sampling duration 2	3 sec.
Contrasts	1
TE	1.12 ms
TR	293.56 ms
Recovery duration 1	3 sec.
Recovery duration 2	0 sec.
Motion Correction	Standard
Save original images	On

Inline - MIP

MIP-Sag	Off
MIP-Cor	Off
MIP-Tra	Off
MIP-Time	Off
Save original images	On

Inline - Composing

Inline Composing	Off
Distortion Corr.	On
Mode	2D
Unfiltered images	Off

Sequence - Part 1

Introduction	Off
Dimension	2D
Reordering	Linear
Asymmetric echo	Weak
Contrasts	1
Optimization	Min. TE TR
Multi-slice mode	Sequential
Echo spacing	2.7 ms
Sequence type	Trufi
Bandwidth	1085 Hz/Px

Sequence - Part 2

Define	Shots
Shots per slice	1
Segments	72
Trufi delta freq.	0 Hz
RF pulse type	Fast
Gradient mode	Fast
Excitation	Slice-sel.
Flip angle mode	Constant
Cine	Off

© 2025 Steffen Johansson R et al. JAMA Network Open.

Sequence - Special

Partition coeff map	Off
ECV map	Off
Synth ECV map	Off
TI scout mode	Off
Error map	On
Synth PSIR	Off
Periods in seconds	On
16 bit images	Off
T1 sampling scheme	Native

Sequence - Assistant

Mode	Off
Allowed delay	0 s

© 2025 Steffen Johansson R et al. JAMA Network Open.

\\USER\Kliniska hjartan\Hjärtprotokollet\COVID 6m\post_MOLLI_4ch_NoScout
TA: 0:13 PM: FIX Voxel size: 1.4×1.4×8.0 mmPAT: 2 Rel. SNR: 1.00 : wip_tfi

Properties

Prio recon	Off
Load images to viewer	On
Inline movie	Off
Auto store images	On
Load images to stamp segments	Off
Load images to graphic segments	On
Auto open inline display	Off
Auto close inline display	Off
Start measurement without further preparation	Off
Wait for user to start	Off
Start measurements	Single measurement

Routine

Slice group	1
Slices	1
Dist. factor	20 %
Position	Isocenter
Orientation	Transversal
Phase enc. dir.	A >> P
AutoAlign	---
Phase oversampling	0 %
FoV read	360 mm
FoV phase	75.0 %
Slice thickness	8.0 mm
TR	293.56 ms
TE	1.12 ms
Averages	1
Concatenations	1
Filter	Distortion Corr.(2D)
Coil elements	BO1-3;SP2,3

Contrast - Common

TR	293.56 ms
TE	1.12 ms
Magn. preparation	Non-sel. IR T1map
TI	193 ms
Flip angle	35 deg
Fat suppr.	Fat sat.
Wrap-up Magn.	None

Contrast - Dynamic

Averages	1
Averaging mode	Short term
Reconstruction	Magnitude
Measurements	1
Multiple series	Off

Resolution - Common

FoV read	360 mm
FoV phase	75.0 %
Slice thickness	8.0 mm
Base resolution	256
Phase resolution	75 %
Phase partial Fourier	7/8
Trajectory	Cartesian
Interpolation	Off

Resolution - iPAT

PAT mode	GRAPPA

Resolution - iPAT

Accel. factor PE	2
Ref. lines PE	36
Reference scan mode	GRE/separate

Resolution - Filter Image

Image Filter	Off
Distortion Corr.	On
Mode	2D
Unfiltered images	Off
Prescan Normalize	Off
Normalize	Off
B1 filter	Off

Resolution - Filter Rawdata

Raw filter	Off
Elliptical filter	Off
POCS	Off

Geometry - Common

Slice group	1
Slices	1
Dist. factor	20 %
Position	Isocenter
Orientation	Transversal
Phase enc. dir.	A >> P
FoV read	360 mm
FoV phase	75.0 %
Slice thickness	8.0 mm
TR	293.56 ms
Multi-slice mode	Sequential
Series	Interleaved
Concatenations	1

Geometry - AutoAlign

Slice group	1
Position	Isocenter
Orientation	Transversal
Phase enc. dir.	A >> P
AutoAlign	---
Initial Position	Isocenter
Phase	0.0 mm
Read	0.0 mm
Shift	0.0 mm
Initial Rotation	0.00 deg
Initial Orientation	Transversal

Geometry - Saturation

Fat suppr.	Fat sat.
Wrap-up Magn.	None
Special sat.	None

Geometry - Navigator

Geometry - Tim Planning Suite

Set-n-Go Protocol	Off
Table position	H
Table position	0 mm
Inline Composing	Off

© 2025 Steffen Johansson R et al. JAMA Network Open.

System - Miscellaneous

Positioning mode	FIX
Table position	H
Table position	0 mm
MSMA	S - C - T
Sagittal	R >> L
Coronal	A >> P
Transversal	F >> H
Coil Combine Mode	Adaptive Combine
Save uncombined	Off
Matrix Optimization	Off
Coil Focus	Flat
AutoAlign	---
Coil Select Mode	Off - All

System - Adjustments

B0 Shim mode	Cardiac
Adjust with body coil	On
Confirm freq. adjustment	Off
Assume Dominant Fat	Off
Assume Silicone	Off
Adjustment Tolerance	Auto

System - Adjust Volume

Position	Isocenter
Orientation	Transversal
Rotation	0.00 deg
A >> P	270 mm
R >> L	360 mm
F >> H	8 mm
Reset	Off

System - Tx/Rx

Frequency 1H	63.672141 MHz
Correction factor	1
Gain	High
Img. Scale Cor.	1.000
Reset	Off
? Ref. amplitude 1H	0.000 V

Physio - Signal1

1st Signal/Mode	ECG/Trigger
Average cycle	430 ± 159 ms
Average cycle	No Signal ms
Captured cycle	430 ± 159 ms
Acquisition window	618 ms
Trigger pulse	1
Trigger delay	324 ms
TR	293.56 ms
Concatenations	1
Segments	72
Phases	1
Adaptive Triggering	Off

Physio - Cardiac

Tagging	None
Magn. preparation	Non-sel. IR T1map
TI	193 ms
Fat suppr.	Fat sat.
Dark blood	Off
FoV read	360 mm
FoV phase	75.0 %
Phase resolution	75 %
Cine	Off
Trajectory	Cartesian

Physio - Cardiac

Dummy heartbeats	0
Motion Correction	Standard

Physio - PACE

Resp. control	Off
Concatenations	1

Inline - Common

Subtract	Off
Measurements	1
StdDev	Off
Motion Correction	Standard
Save original images	On

Inline - Cardiac

Inline Evaluation	T1 map
Magn. preparation	Non-sel. IR T1map
Num. of preps	2
Sampling duration 1	5 sec.
Sampling duration 2	3 sec.
Contrasts	1
TE	1.12 ms
TR	293.56 ms
Recovery duration 1	3 sec.
Recovery duration 2	0 sec.
Motion Correction	Standard
Save original images	On

Inline - MIP

MIP-Sag	Off
MIP-Cor	Off
MIP-Tra	Off
MIP-Time	Off
Save original images	On

Inline - Composing

Inline Composing	Off
Distortion Corr.	On
Mode	2D
Unfiltered images	Off

Sequence - Part 1

Introduction	Off
Dimension	2D
Reordering	Linear
Asymmetric echo	Weak
Contrasts	1
Optimization	Min. TE TR
Multi-slice mode	Sequential
Echo spacing	2.7 ms
Sequence type	Trufi
Bandwidth	1085 Hz/Px

Sequence - Part 2

Define	Shots
Shots per slice	1
Segments	72
Trufi delta freq.	0 Hz
RF pulse type	Fast
Gradient mode	Fast
Excitation	Slice-sel.
Flip angle mode	Constant
Cine	Off

© 2025 Steffen Johansson R et al. JAMA Network Open.

Sequence - Special

Partition coeff map	Off
ECV map	Off
Synth ECV map	Off
TI scout mode	Off
Error map	On
Synth PSIR	Off
Periods in seconds	On
16 bit images	Off
T1 sampling scheme	Native

Sequence - Assistant

Mode	Off
Allowed delay	0 s

© 2025 Steffen Johansson R et al. JAMA Network Open.

\\USER\Kliniska hjartan\Hjärtprotokollet\COVID 6m\FB_DB_PSIR
TA: 1:23 PM: FIX Voxel size: 1.4×1.4×8.0 mmPAT: 2 Rel. SNR: 1.00 : adT2

Properties

Prio recon	Off
Load images to viewer	Off
Inline movie	Off
Auto store images	On
Load images to stamp segments	Off Load
images to graphic segments	On Auto
open inline display	Off
Auto close inline display	Off
Start measurement without further preparation	Off
Wait for user to start	On
Start measurements	Single measurement

Routine

Slice group	1
Slices	3
Dist. factor	0 %
Position	Isocenter
Orientation	Transversal
Phase enc. dir.	A >> P
AutoAlign	---
Phase oversampling	0 %
FoV read	360 mm
FoV phase	75.0 %
Slice thickness	8.0 mm
TR	349.50 ms
TE	1.18 ms
Averages	16
Concatenations	3
Filter	Distortion Corr.(2D)
Coil elements	BO1-3;SP2,3

Contrast - Common

TR	349.50 ms
TE	1.18 ms
TD	0 ms
Magn. preparation	T2 prep. adiab.
T2 prep. duration	45 ms
Flip angle	50 deg
Fat suppr.	None
Wrap-up Magn.	None

Contrast - Dynamic

Averages	16
Averaging mode	Short term
Reconstruction	Magnitude/Real
Measurements	1
Multiple series	Off

Resolution - Common

FoV read	360 mm
FoV phase	75.0 %
Slice thickness	8.0 mm
Base resolution	256
Phase resolution	75 %
Phase partial Fourier	Off
Trajectory	Cartesian
Interpolation	Off

Resolution - iPAT

PAT mode	GRAPPA
Accel. factor PE	2
Ref. lines PE	32
Reference scan mode	GRE/separate

Resolution - Filter Image

Image Filter	Off
Distortion Corr.	On
Mode	2D
Unfiltered images	Off
Prescan Normalize	Off
Normalize	Off
B1 filter	Off

Resolution - Filter Rawdata

Raw filter	Off
Elliptical filter	Off
POCS	Off

Geometry - Common

Slice group	1
Slices	3
Dist. factor	0 %
Position	Isocenter
Orientation	Transversal
Phase enc. dir.	A >> P
FoV read	360 mm
FoV phase	75.0 %
Slice thickness	8.0 mm
TR	349.50 ms
Multi-slice mode	Sequential
Series	Interleaved
Concatenations	3

Geometry - AutoAlign

Slice group	1
Position	Isocenter
Orientation	Transversal
Phase enc. dir.	A >> P
AutoAlign	---
Initial Position	Isocenter
Phase	0.0 mm
Read	0.0 mm
Shift	0.0 mm
Initial Rotation	0.00 deg
Initial Orientation	Transversal

Geometry - Saturation

Fat suppr.	None
Wrap-up Magn.	None
Special sat.	None

Geometry - Navigator

Geometry - Tim Planning Suite

Set-n-Go Protocol	Off
Table position	H
Table position	0 mm
Inline Composing	Off

© 2025 Steffen Johansson R et al. JAMA Network Open.

© 2025 Steffen Johansson R et al. JAMA Network Open.

eTable. Perfusion and MPR subgroup analysis

Subgroup analysis COVID-19 patients	Stress perfusion	Rest perfusion	MPR
**Male vs female**	2.7 [2.4, 3.1] vs 3.0 [2.3, 3.7], *p*=0.51	1.0 [0.9, 1.2] vs 1.3 [0.9, 1.8], *p*=0.31*	2.79 [2.44, 3.06] vs 2.34 [1.86, 2.82], *p*=0.21
**Hypertension (yes/no)**	2.8 [1.7, 4.0] vs 2.8 [2.5, 3.1], *p*=0.94	1.1 [0.6, 1.7] vs 1.1 [1.0, 1.2], *p*=0.74*	2.62 [1.61, 3.63] vs 2.70 [2.38, 3.03], *p*=0.84
**Hyperlipidemia (yes/no)**	3.5 [2.5, 4.4] vs 2.7 [2.4, 3.0], *p*=0.08	1.6 [0.6, 2.6] vs 1.1 [0.9, 1.2], *p*=0.08*	2.39 [1.28, 3.51] vs 2.72 [2.40, 3.05], *p*=0.48
**Diabetes mellitus (yes/no)**	3.4 [-1.5, 8.2] vs 2.8 [2.5, 3.1], *p*=0.47	1.5 [-7.6, 10.6] vs 1.1 [1.0, 1.2], *p*=0.62*	2.64 [-9.81, 15.10] vs 2.69 [2.39, 2.99], *p*=0.47
**Cigarette smoking (yes/no)**	2.7 [2.4, 3.1] vs 2.9 [2.4, 3.3], *p*=0.68	1.1 [0.9, 1.3] vs 1.1 [0.9, 1.3], *p*=0.65*	2.74 [2.16, 3.32] vs 2.64 [2.35, 2.93], *p*=0.68
**Chest pressure (yes/no)**	3.1 [2.5, 3.7] vs 2.7 [2.4, 3.0], *p*=0.26	1.0 [0.8, 1.2] vs 1.1 [1.0, 1.3], *p*=0.76*	3.34 [2.27, 4.41] vs 2.53 [2.25, 2.81], *p*=0.025
**Dyspnea (yes/no)**	2.9 [2.4, 3.4] vs 2.7 [2.4, 3.1], *p*=0.65	1.0 [0.9, 1.2] vs 1.2 [1.0, 1.3], *p*=0.56*	2.89 [2.36, 3.43] vs 2.52 [2.18, 2.87], *p*=0.21
**Fatigue (yes/no)**	2.8 [2.3, 3.3] vs 2.8 [2.5, 3.2], *p*=0.99	1.1 [0.9, 1.3] vs 1.1 [0.9, 1.3], *p*=0.75*	2.62 [2.21, 3.05] vs 2.72 [2.30, 3.14], *p*=0.78

Subgroup analysis comparing COVID-19 patients based on sex, presence of hypertension, hyperlipidemia, diabetes mellitus, cigarette smoking, chest pressure, dyspnea or fatigue. Data presented as medan [95% confidence interval], p-values denotes the independent t-test, and p-values marked * denotes the Mann Whitney U test. Abbreviations: COVID-19 = Coronavirus disease 2019; MPR = myocardial perfusion reserve.

© 2025 Steffen Johansson R et al. JAMA Network Open.
