# Supplementary material for: Long-Term Coronary Microvascular and Cardiac Dysfunction After Severe COVID-19 Hospitalization
Source: JAMA Netw Open. 2025 Jun 9;8(6):e2514411. doi: 10.1001/jamanetworkopen.2025.14411 (PMC12150193; doi:10.1001/jamanetworkopen.2025.14411)
Supplement: Supplement 2. — Data Sharing Statement [file jamanetwopen-e2514411-s002.pdf]

Data Sharing Statement

Steffen Johansson. Long-Term Coronary Microvascular and Cardiac Dysfunction After Severe COVID-19 Hospitalization. *JAMA Netw Open*. Published online June 9, 2025.
doi:10.1001/jamanetworkopen.2025.14411

Data

Data available: No

Additional Information

Explanation for why data not available: The data supporting the findings are available from corresponding author upon reasonable request.
